# A new approach to assess the degree of contamination and determine sources and risks related to PTEs in an urban environment: the case study of Santiago (Chile)

**DOI:** 10.1007/s10653-021-01185-6

**Published:** 2022-01-10

**Authors:** Antonio Aruta, Stefano Albanese, Linda Daniele, Claudia Cannatelli, Jamie T. Buscher, Benedetto De Vivo, Attila Petrik, Domenico Cicchella, Annamaria Lima

**Affiliations:** 1 Department of Earth, Environmental and Resources Sciences, University of Naples Federico II, 80126 Naples, Italy; 2 Department of Geology, FCFM, Andean Geothermal Center of Excellence (CEGA) and Millenium Nucleus for Metal Tracing Along Subduction, Universidad de Chile, Plaza Ercilla 803, Santiago, Chile; 3 University of Alaska Anchorage, 3211 Providence Drive, Anchorage, AK 99508 USA; 4 Virginia Tech, Blacksburg, VA 24061 USA; 5 Pegaso On Line University, Piazza Trieste e Trento 48, 80132 Naples, Italy; 6 Eriksfiord AS, Prof. Olav Hanssensvei 7A, 4021 Stavanger, Norway; 7 Department of Science and Technology, University of Sannio, 82100 Benevento, Italy

**Keywords:** Urban geochemistry, Contamination degree assessment, Multifractal IDW, Compositional data analysis (CoDA), Proabilistic risk assessment, Monte Carlo method

## Abstract

In 2017, a geochemical survey was carried out across the Commune of Santiago, a local administrative unit located at the center of the namesake capital city of Chile, and the concentration of a number of major and trace elements (53 in total) was determined on 121 topsoil samples. Multifractal IDW (MIDW) interpolation method was applied to raw data to generate geochemical baseline maps of 15 potential toxic elements (PTEs); the concentration–area (C-A) plot was applied to MIDW grids to highlight the fractal distribution of geochemical data. Data of PTEs were elaborated to statistically determine local geochemical baselines and to assess the spatial variation of the degree of soil contamination by means of a new method taking into account both the severity of contamination and its complexity. Afterwards, to discriminate the sources of PTEs in soils, a robust Principal Component Analysis (PCA) was applied to data expressed in isometric log-ratio (ilr) coordinates. Based on PCA results, a Sequential Binary Partition (SBP) was also defined and balances were determined to generate contrasts among those elements considered as proxies of specific contamination sources (Urban traffic, productive settlements, etc.). A risk assessment was finally completed to potentially relate contamination sources to their potential effect on public health in the long term. A probabilistic approach, based on Monte Carlo method, was deemed more appropriate to include uncertainty due to spatial variation of geochemical data across the study area. Results showed how the integrated use of multivariate statistics and compositional data analysis gave the authors the chance to both discriminate between main contamination processes characterizing the soil of Santiago and to observe the existence of secondary phenomena that are normally difficult to constrain. Furthermore, it was demonstrated how a probabilistic approach in risk assessment could offer a more reliable view of the complexity of the process considering uncertainty as an integral part of the results.

**Supplementary Information:**

The online version contains supplementary material available at 10.1007/s10653-021-01185-6.

## Introduction

Anthropogenic contamination may have a negative impact on both life quality and expectancy in urbanized areas. (Albanese & Cicchella, 2012; Chambers et al., 2016; Filippelli et al., 2012; Konstantinova et al., 2019). Generally, soils can be considered passive collectors of contaminants derived from atmospheric fallout, water runoff and local spills, so they can be used as an effective tool to locally assess the level of interaction between humans and the surrounding environment.

Due to their high potential for ecological transfer, the concentration of potentially toxic elements (PTE) in urban soils is normally used as a raw indicator of environmental conditions.

In order to control the effects of human activities on the environment, many countries around the world, at different times, have introduced legislative tools to safeguard the environment and, hence, public health. Unfortunately, Chile, where 87% of the total population (ca. 18 million) lives in urban areas, is one of the countries that does not have any specific regulations to assess both the hazards associated with potentially contaminated soils and the risk posed by contaminants to human health. In 2013, the Chilean Ministry of the Environment (Ministerio del Medio Ambiente) released a guide (Resolución Exenta 406/2013) aiming at the management of soils potentially affected by the presence of contaminants (MMA, CORFO, & Fundación Chile, 2012) with reference values for different land uses (Residential, Agricultural, Industrial) retrieved from international sources. However, to be effective, environmental guidelines, especially in the case of soil, should be determined considering the compositional features of samples proceeding from places holding the same characteristics of the contaminated lands. Therefore, the definition of local geochemical background/baseline intervals (Reimann et al., 2005) for elements of concern is a key step in the challenging process toward the assessment of the degree of contamination of a specific territory. Besides, for a successful management of environmental risks, in sub-regional, urban and, in any case, non-site-specific scale studies also the discrimination of multiple sources of contamination together with the assessment of the risk in a probabilistic perspective (to consider the uncertainty generated by the spatial variability of elemental patterns) are relevant points.

In light of the above considerations, this study, using the geochemical data generated for the Commune of Santiago in Chile, proposes new approaches for the evaluation of the degree of contamination of soils. In addition, considering the current advances in the compositional data analysis (CoDA), some techniques for both the identification of potentially toxic elements (PTE) sources and the stochastic assessment of health risks in urban areas are also presented and discussed.

## Study area

**Fig. 1:**
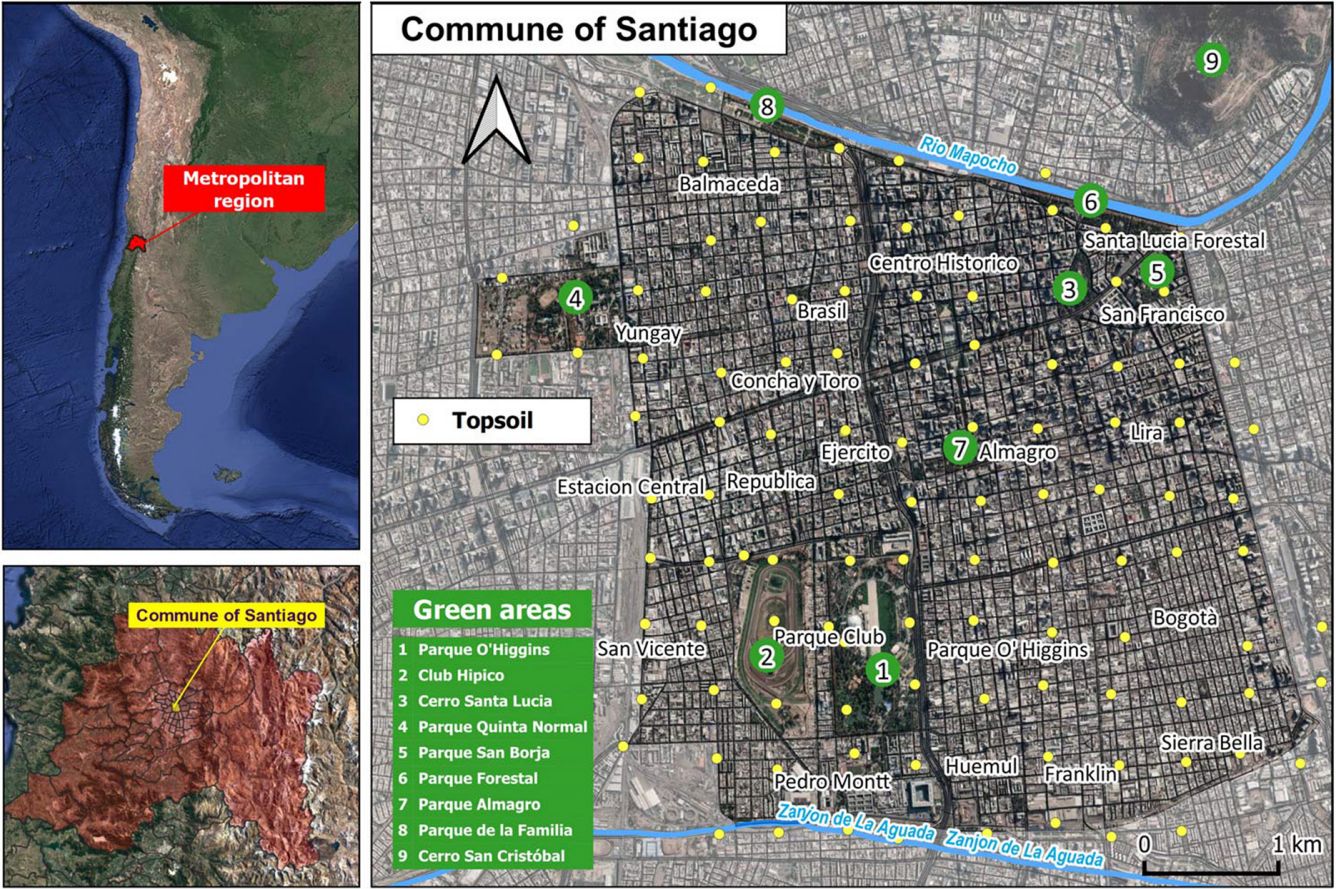
Satellite images of the western coast of South America (top left) and Santiago (bottom left) and a close-up satellite map of the study area (right) with sampling locations of collected topsoils. The basemaps are retrieved from Google Satellite through QGIS. The numbers indicate the main green areas of the Commune of Santiago

Our study area, covering 22.4 km^2^, corresponds to the Commune of Santiago (Fig. 1) which is a local administrative unit located at the center of the territory of the Chilean capital city (known also as the “Greater Santiago”) with a total population of 404,495 and an estimated population density of 17.485,2 hab/km^2^ (Istituto National de Estatistica, 2018). This latter is in the middle of a morphological depression (“Santiago Basin”) lying between two N-S striking mountain ranges: the Coastal Cordillera on the west and the Andean Cordillera on the east. Both mountain chains consist of volcanic and sedimentary sequences, along with intrusive rocks formed from the Upper Jurassic to Cretaceous (Coastal Cordillera) and in the Cenozoic (Andean Cordillera) (Charrier et al., 2002_Bibliografia:). The basin has an average elevation of about 250 m a.s.l (Yáñez et al., 2015) and since the Pleistocene, it has been filled by fluvial and fluvial-alluvial deposits originating from the Andean Cordillera and transported by the fluvial system of the Maipo river (Rauld, 2011). Fine lake sediments are also locally present in the northwest and southwest parts of the basin. Pyroclastic deposits (Ignimbrita de Pudahuel) corresponding to Pleistocene events (Stern et al., 1984) are also found in the west-central part of the basin, intercalated in places with fine alluvial sediments.

Soils of Santiago, developed on the fluvial-alluvial deposits of volcanic origin, especially in the study area, have lost their original structure and composition since they have been strongly modified by the impact of the long-lasting urbanization history, dating back to the sixteenth century. Nowadays, only 2.47 km^2^ of the Commune is occupied by green areas and parks, probably less altered in terms of composition, whereas up to 20 km^2^ are occupied by mixed residential areas where commercial activities and services coexist with private dwellings (Fig. 1).

In the Commune of Santiago, which is the main city hub for public and private transportation and the physical center of the main Chilean government functions, air pollution represents the primary environmental contamination source, although in the last two decades it has showed a strong decreasing decadal linear trend (Gallardo et al., 2018). Specifically, the environmental degradation of the area is mostly attributed to the fossil fuel combustion processes related with energy production and industrial activities (occurring, especially, in the territories surrounding the study area), to the intense inbound and outbound daily vehicular traffic and to the resuspension of soil and road dusts (Artaxo et al., 1999).

## Materials and methods

### Sampling and analytical methods

In the spring of 2017, 121 topsoil samples were collected at a depth interval ranging from 0 to 10 cm with a nominal sampling density of 1 sample per 0.25 km^2^ following the advice provided by Demetriades et Birke (2015) in the framework of the second EuroGeoSurveys' Urban Geochemistry Project (URGE II) which brought to excellent results in European countries (Johnson et al., 2011). Spatial coordinates of each soil sample were acquired with a hand-held GPS device: 85% of the samples were collected within residential areas and 15% were from parks and gardens (Fig. 1).

Specifically, at each sampling station, the soil was excavated from a 50 × 50 cm area and a representative homogenized soil sample of almost 1 kg was collected into Rilsan® bags. Stainless steel and paint-free tools, that were carefully cleaned before and after the collection of each sample, were used to collect soils. Each bag was properly labeled and tightly sealed to avoid any accidental contamination and/or loss of material. After sampling, a vertical scale was placed into the excavated soil pit and several pictures were taken and included in a field report. In the field, notes about the surrounding landscape (topography, presence of outcrops, potential sources of contamination, air quality status, land use) were taken during the sampling process; soil characteristics (presence of soil stratification, granulometry, humidity, abundance of both organic matter and clasts > 2 mm) were also assessed in a semi-quantitative way based on a direct observation of sampled materials. However, this latter information was not deemed appropriate to be used later in the data elaboration process due to their subjective nature although we are aware that they would have been a valuable tool to improve the interpretation of elemental geochemical patterns.

Collected samples were transported to the Environmental Sample Preparation Lab (ESPL) at the Geology Department at the University of Chile and air dried at room temperature (< 37 °C) to avoid loss of Hg. Stainless steel sieves were used to collect material < 2 mm from bulk samples and to prepare 30 g aliquots for shipment to the Bureau Veritas Laboratory of Vancouver (Canada). Analyses were performed by an ultratrace ICP-MS following a further grinding of material to a 0.075 mm grain size and a modified Aqua Regia (HNO_3_ + 3HCl) digestion which can be considered a method to extract “quasi-total” concentrations (Albanese, 2007) and it is used to assess human health risk (Gupta et al., 1996) assuming a 100% bioavailability (worst-case scenario) of a contaminant of concern (DEFRA, ). The concentration of 53 chemical elements (Ag, Al, As, Au, B, Ba, Be, Bi, Ca, Cd, Ce, Co, Cr, Cs, Cu, Fe, Ga, Ge, Hf, Hg, In, K, La Li, Mg, Mn, Mo, Na, Nb, Ni, P, Pb, Pd, Pt, Rb, Re, S, Sb, Sc, Se, Sn, Sr, Ta, Te, Th, Ti, Tl, U, V, W, Y, Zn and Zr) was determined for all of the collected samples.

The quality of analytical measurements for each element was assessed by calculating analytical precision using the relative percentage difference (RPD), and accuracy by the estimation of the accuracy error (HMTRI, 1997) (Supplementary material S1).

Soil pH was also measured using the pH—HQ40D Portable Multi Meter after mixing the soil with distilled water in a 1:1 ratio (20 g moist soil: 20 ml H_2_O) for 30 min (Robertson, 1984).

For the purposes of this study, only a selection of analytes corresponding to a total of 15 potentially toxic elements (PTEs) (As, Be, Cd, Co, Cr, Cu, Hg, Mo, Ni, Pb, Sb, Sn, Tl, V and Zn) plus pH were used. Data obtained from the analytical reports were associated with the relative spatial coordinates of the samples and assessed using statistical analysis and mapping.

### Geochemical patterns and potential contamination sources

#### Univariate statistics and multifractal IDW

Prior to the assessment of any potential harm that contaminants could pose to the environment and human beings, it was crucial to determine their spatial distribution and assess the nature of their potential sources. For each PTE, basic univariate statistics were calculated together with the upper baseline limit (UBL) and the coefficient of variation (CV) (Table 1).

**Table 1:** Univariate statistics, coefficient of variation (CV) and Upper Baseline Limit (UBL) of PTEs and pH in the topsoil samples of the study area

Elements	U.M	Min	Max	Mean	Median	M.A.D	St. Dev	Variance	Kurtosis	Skewness	CV (%)	UBL
As	mg/kg	2.60	23.90	13.42	14.10	3.55	4.47	19.94	−0.29	−0.28	33.27	21
Be	mg/kg	0.1	1.0	0.4	0.4	0.1	0.2	0.0	0.3	0.1	38.3	0.7
Cd	mg/kg	0.06	1.97	0.47	0.42	0.23	0.33	0.11	6.53	2.16	69.35	0.9
Co	mg/kg	6.40	23.90	13.82	14.10	2.40	3.23	10.46	1.02	0.27	23.41	19
Cr_tot_	mg/kg	9.30	71.20	25.85	21.90	8.65	11.66	2.52	2.52	1.56	45.09	39
Cu	mg/kg	26.98	1206.03	223.37	188.87	106.98	169.20	28,627.61	12.55	2.98	75.75	403
Mo	mg/kg	1.11	9.25	3.14	2.83	0.95	1.30	1.68	4.36	1.68	41.32	4.7
Ni	mg/kg	4.80	53.10	14.48	1.13	3.47	5.61	31.49	18.40	3.15	38.75	21
Pb	mg/kg	8.31	681.41	106.21	68.92	75.61	114.71	13,159.31	8.86	2.71	108.01	220
Sb	mg/kg	0.30	17.82	2.48	1.61	1.68	2.81	7.88	16.36	3.67	113.05	5
Sn	mg/kg	0.80	75.30	11.91	7.20	9.37	0.86	181.75	7.11	2.43	113.20	26
Tl	mg/kg	0.02	0.24	0.11	0.11	0.03	0.04	0.00	1.08	0.88	32.95	0.17
V	mg/kg	49.00	152.00	93.02	94.00	13.46	19.23	369.84	1.39	0.15	20.67	121
Zn	mg/kg	38.70	977.80	257.46	227.00	112.40	170.63	29,115.12	4.09	1.76	66.28	472
Hg	μg/kg	12.00	3081.00	287.98	201.00	218.28	357.94	128,123.70	31.15	4.55	124.30	0.6
Fe	%	2.08	5.18	3.72	3.78	0.48	0.64	0.41	0.31	−0.34	17.17	47,400
pH	–	5.3	9.29	7.78	7.84	−	0.56	0.31	5.00	−1.17909	0.07	−

Given the lack of previous geochemical data for soils in the study area, for each individual element, the median value ± 2 Median Absolute Deviations (MAD) was utilized (Reimann et al., 2005) to calculate the geochemical baseline interval (which is representative of the actual content of an element in the superficial environment at a given area excluding outliers) (Albanese et al., 2018); subsequently, the upper threshold of the interval was used as the UBL. The CV was determined using the ratio of the standard deviation to the average concentration of each element (Koch & Link, 1971) to define its own extent of variability within the entire geochemical dataset.

To better support the interpretation of geochemical data distribution and to easily identify existing peculiarities, EDA (Exploratory Data Analysis) plots including a combination of a histogram, a normal density function line, a density trace and a boxplot were also drawn using the “StatDA” package (https://www.rdocumentation.org/packages/StatDA/versions/1.7.4) with R software (Supplementary material S2).

**Fig. 2:**
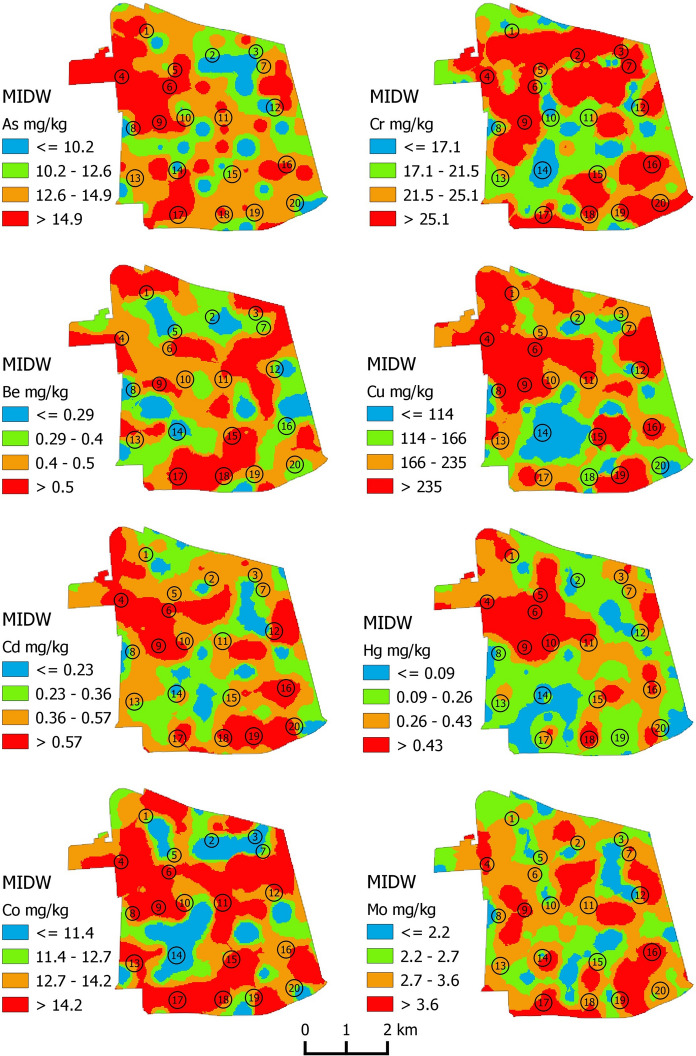
Multifractal Inverse Distance Weighted grid of As, Be, Cd, Co, Cr, Cu, Hg and Mo concentrations in the study area. Map intervals have been defined by means of a C-A plot. Numbers on maps refer to neighborhoods of the Commune: (1) Balmaceda; (2) Centro Historico, (3) Santa Lucia Forestal; (4) Yungay; (5) Brasil; (6) Concha y Toro; (7) San Francisco; (8) Estacion Central; (9) Republica; (10) Ejercito; (11) Almagro; (12) Lira; (13) San Vicente (14) Parque Club; (15) Parque O' Higgins; (16) Bogotà; (17) Pedro Montt; (18) Huemul; (19) Franklin; (20) Sierra Bella

**Fig. 3:**
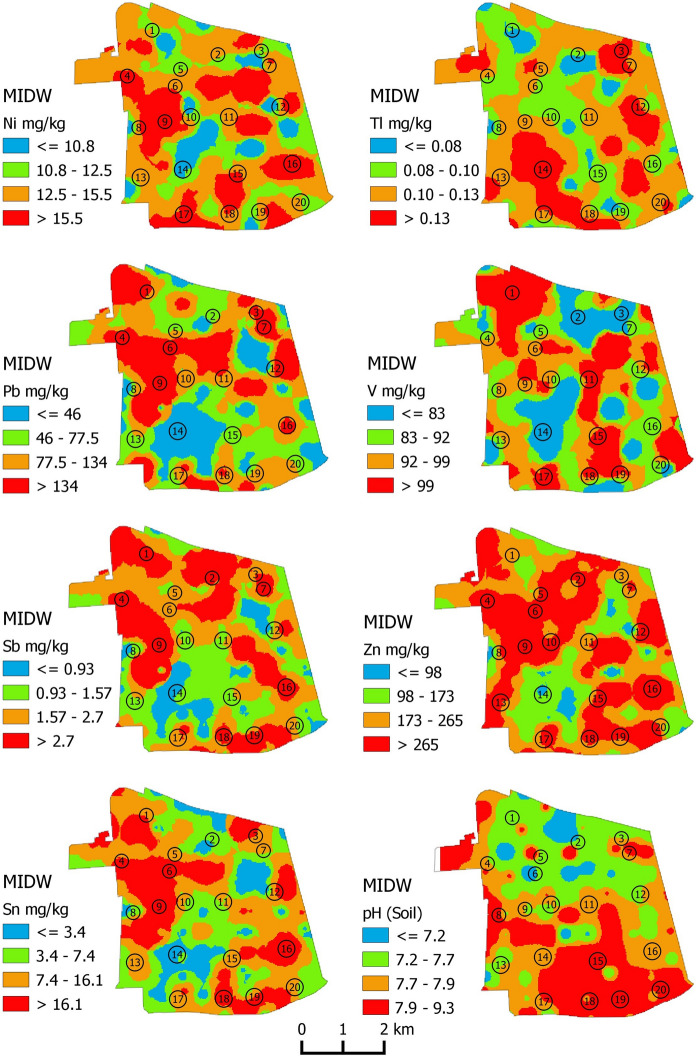
Multifractal Inverse Distance Weighted grid of Ni, Pb, Sb, Tl, V, Zn concentrations and, additionally, pH soil values in the study area. Map intervals have been defined by means of a C–A plot. Numbers on maps refer to neighborhoods of the Commune as reported in caption of Fig. 2

The entire geochemical dataset was georeferenced, and interpolated maps of the 15 selected PTEs were generated (Figs. 2 and 3). The Multifractal Inverse Distance Weighting (MIDW) interpolation technique was applied to the data using the software GeoDAS (Albanese et al., 2007; Cheng et al., 2001). To classify the MIDW grids through concentration intervals mostly reflecting the natural or anthropogenic processes underlying the data variability, the concentration–area (C-A) plot was applied using the ArcFractal add-in for ArcGIS (Zuo & Wang, 2020). In fact, for each of the MIDW grids, four concentration intervals were shown on the map based on the selection of the main marked inflexion points found along the relative C-A curve (Supplementary material S3).

#### Contamination assessment

For each sample in the dataset relative to each single PTE, the value of the individual contamination index (ICI) was calculated by applying Eq. 1 to the raw geochemical data:

1 $$ICI_{i} = \frac{{\left( {\frac{{C_{i} }}{{N_{i} }}} \right)}}{{\left( {\frac{{C - UBL_{i} }}{{N - UBL_{i} }}} \right)}}$$

where

- *C*_*i*_ and *N*_*i*_ are the concentrations for the i-esim PTE element and a normalizing element, respectively
- *C-UBL*_*i*_ and *N-UBL*_*i*_ are the UBL values of the same PTE and the normalizing element, respectively.

In Eq. 1, Fe was chosen as a normalizing element on the basis of its low variability according to its CV in topsoils (Table 1).

**Fig. 4:**
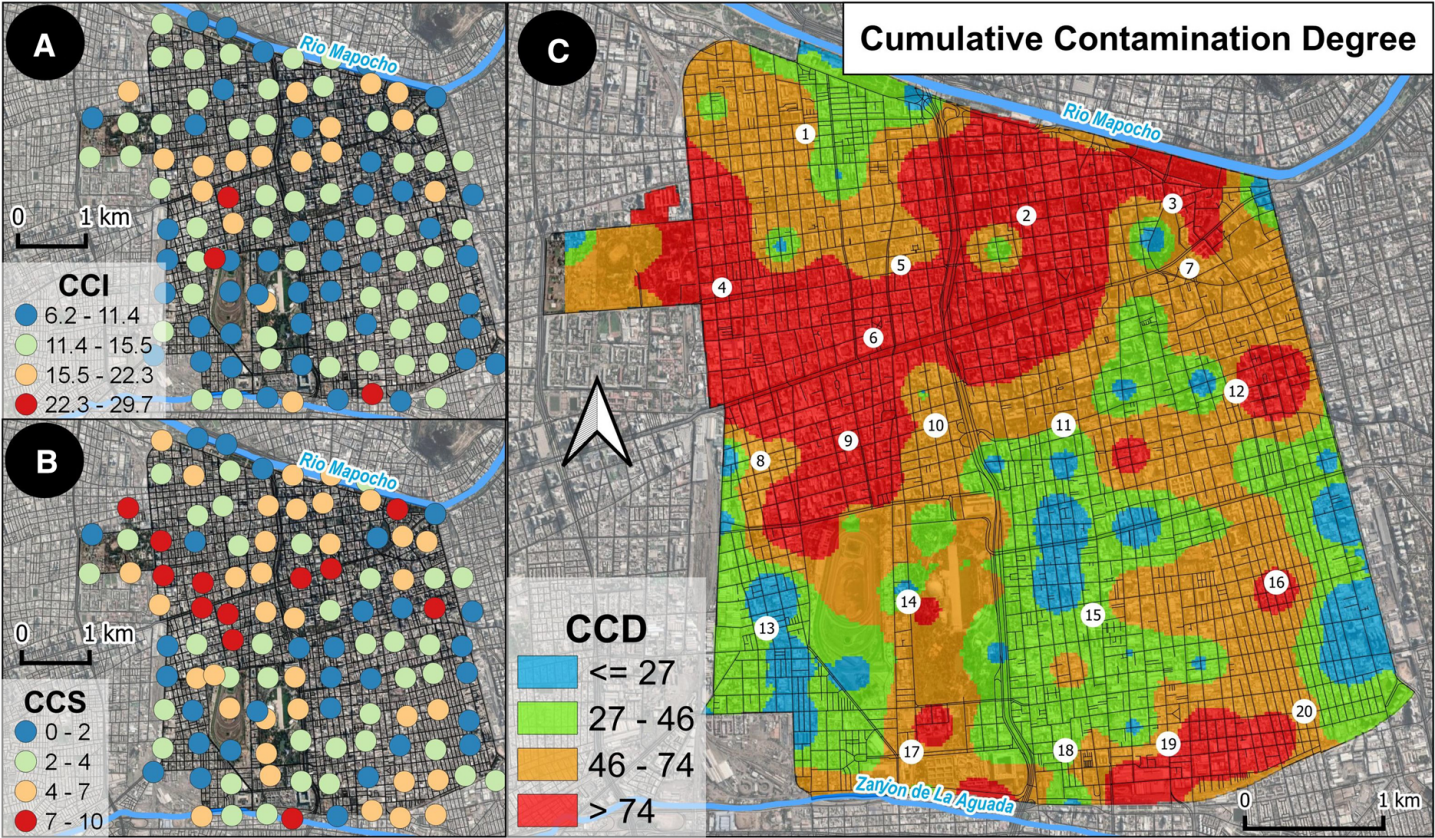
Dot maps of the CCI (**A**) and CCS (**B**) values across the study area. Interpolated distribution of CCD values (**C**). Vector layers representing the official borders of neighborhoods (Barrios) and the road network within the Commune of Santiago superimposed on the CCD raster map. Numbers on CCD map refer to neighborhoods of the Commune as reported in caption of Fig. 2

A cumulative contamination index (CCI) was then calculated for each sample in the database by adding together the ICIs of the 15 PTEs considered; the resulting values were plotted on a dot map by using the natural breaks (Jenks) as a classification method for the CCI values (Fig. 4A).

A cumulative contamination score (CCS), representing the total number of PTEs exceeding their relative UBL at each sampling point, was also assigned to each sample by following a two-step procedure:

1. reclassifying individual ICI values on a boolean basis, assigning the “0” value to ICI < 1 and the “1” value to ICI ≥ 1.
2. adding up all of the reclassified ICI values according to Eq. 2:
  2 $$CCS = \sum {\text{Re}} cICI_{i}$$

where

- Is the reclassified ICI value of the i-esim PTE in the dataset.

The CCS dot map was generated and is shown in Fig. 4B as well.

Finally, with the purpose of generating a comprehensive value to represent the degree of contamination at each sampling point, considering both the severity of contamination (possibly represented by CCI) and its complexity (possibly represented by the CCS), a cumulative contamination degree (CCD) was determined by Eq. 3:

3 $$CCD = CCI*CCS$$

A MIDW interpolated map of CCD values (Fig. 4C) was generated, and the C-A plot technique was used to separate values into intervals potentially highlighting underlying contamination processes occurring at specific areas in the study area.

#### Robust multivariate analysis

With the purpose of identifying the main sources of PTEs in the soils of the Commune of Santiago, a multivariate statistical technique, Principal Component Analysis (PCA), was chosen as the tool to explain the correlation structure of the elements through a reduced number of “components”. However, it cannot be ignored that geochemical data (in raw format) represent a specific case of compositional data in which positive vectors only carry information of a part relative to the whole (Aitchison, 1982; Pawlowsky-Glahn & Buccianti, 2011; Pawlowsky-Glahn et al., 2015; Tolosana-Delgado & Boogaart, 2013), implying that they are affected by a so-called closure problem. In fact, the increase of the concentration of one element in the dataset leads to a forced decrease of the value of the other elements (Chayes, 1960), and these constraints could have strong implications on the statistical treatment of geochemical data, especially from a multivariate perspective (McKinley et al., 2016).

Aitchison (1986) and Egozcue et al. (2003) elaborated several log-ratio transformations to “open” the data structure and analyze them in real Euclidean space. All transformations, including pairwise-, additive-logratio (alr), centred-logratio (clr) and isometric-logratio (ilr), have limitations as summarized by McKinley et al. (2016), but they can be applied to compositional data to overcome the closure effect prior to analyses.

In light of the above consideration, Principal Component Analysis (PCA) was performed on geochemical data following the procedure of Filzmoser et al. (2009). To robustify the analysis, covariance was determined after the application of an ilr transformation to geochemical data; the transformation was applied with the purpose of minimizing the presence of both outliers and spurious correlations (Pawlowsky-Glahn & Buccianti, 2011). PCA was completed after the ilr data (including loading, scores and eigenvalues) were back-transformed to the centered log-ratio (clr) space. The pH value of soils was also included in the process as an external (non-compositional) variable.

The robust PCA was performed by means of the “pcaCoDa” function available in the “RobComposition” package (https://www.rdocumentation.org/packages/robCompositions/versions/2.0.0/topics/pcaCoDa) within the R software framework. Results of the PCA were extracted and visually explored by means of the “Factoextra” package (https://www.rdocumentation.org/packages/factoextra/versions/1.0.7) also available for R.

**Fig. 5:**
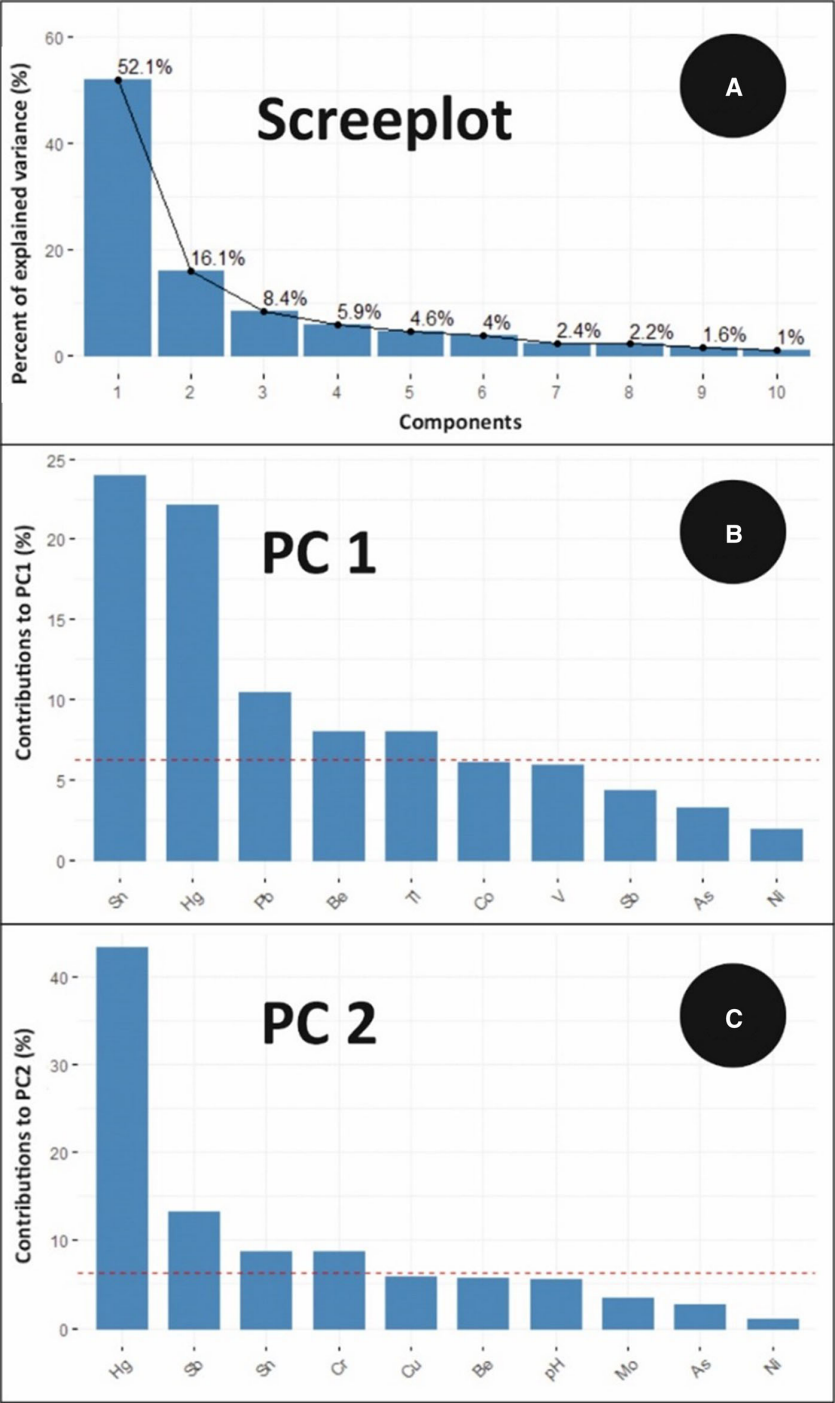
PCA scree plot (**A**) and contribution of single variables to PC1 (**B**) and PC2 (**C**). The red dashed lines represent the cutoff applied to discriminate variables significantly contributing to each component. Details on how the cutoffs have been established are reported in the text

Based both on the percent variance explained by each of the 15 components extracted and the observation of the scree plot (Fig. 5A), two PCs (accounting together for 68.2% of the total variance) were considered reliable for the purpose of the identification of the main sources. Specifically, PC1 and PC2 accounted for 52.1% and 16.1% of the total explained variance, respectively.

The contribution of each variable to individual components was determined and a reference cutoff value was calculated based on the inverse of the total number of involved variables (15) expressed as a percentage (i.e. 6%) (Fig. 5B, C). As a consequence, Sn, Hg, Pb, Be and Tl on one side, and Hg, Sb, Sn and Cr on the other side are considered as relevant contributors to PC1 and PC2, respectively.

**Fig. 6:**
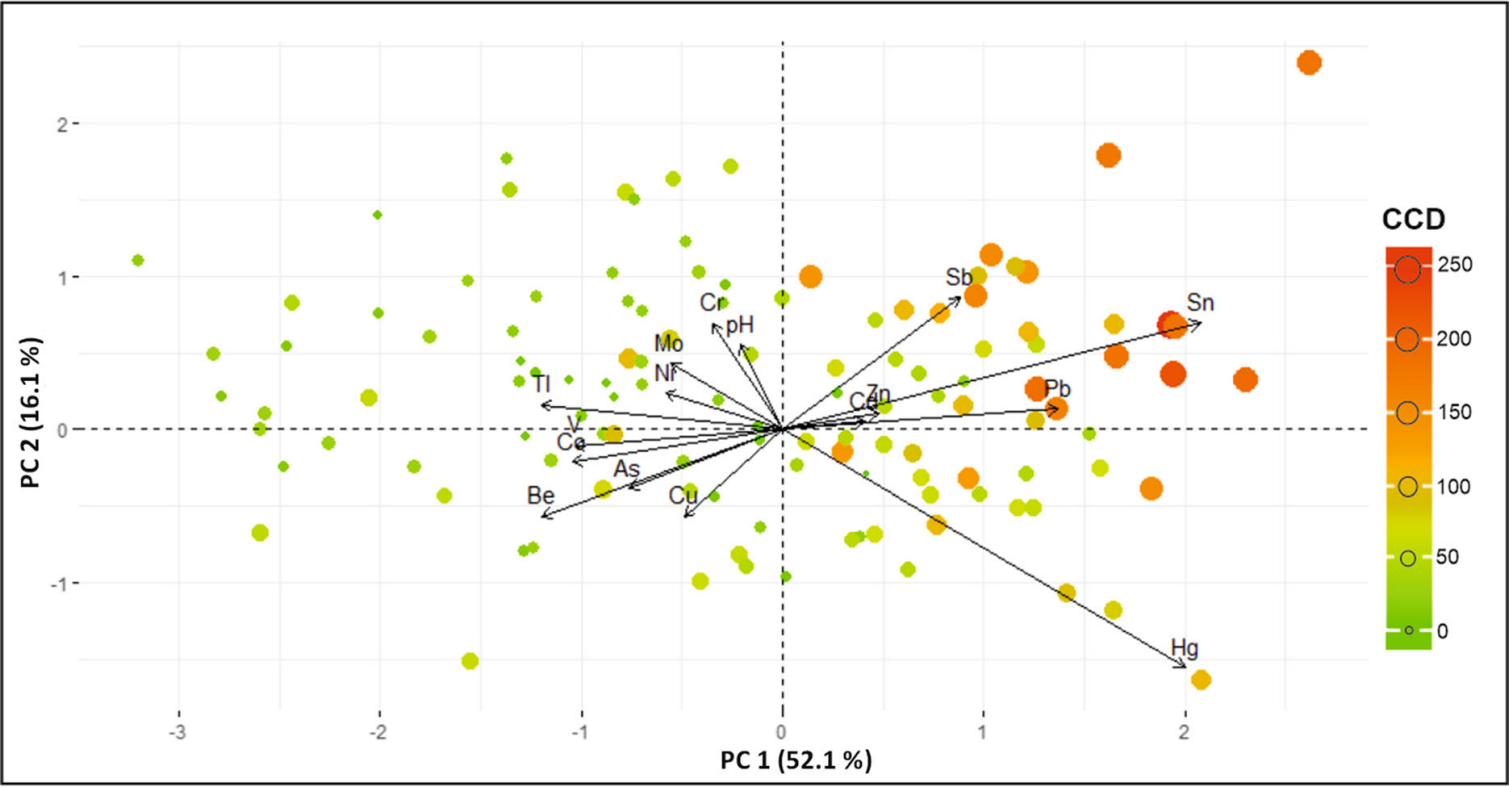
Biplots of PC1 vs. PC2. Observations (samples) are reported as single dots; dimensions and colors of each dot vary according to the CCD value of the related sample

A comprehensive view of the PCA results was obtained by generating a biplot of PC1 and PC2 (Fig. 6). A biplot integrates both variables (PTEs) and observations (samples). Observations were reported in the plot as single dots whose dimension and color varied according to the corresponding CCD value as calculated using Eq. 3.

**Fig. 7:**
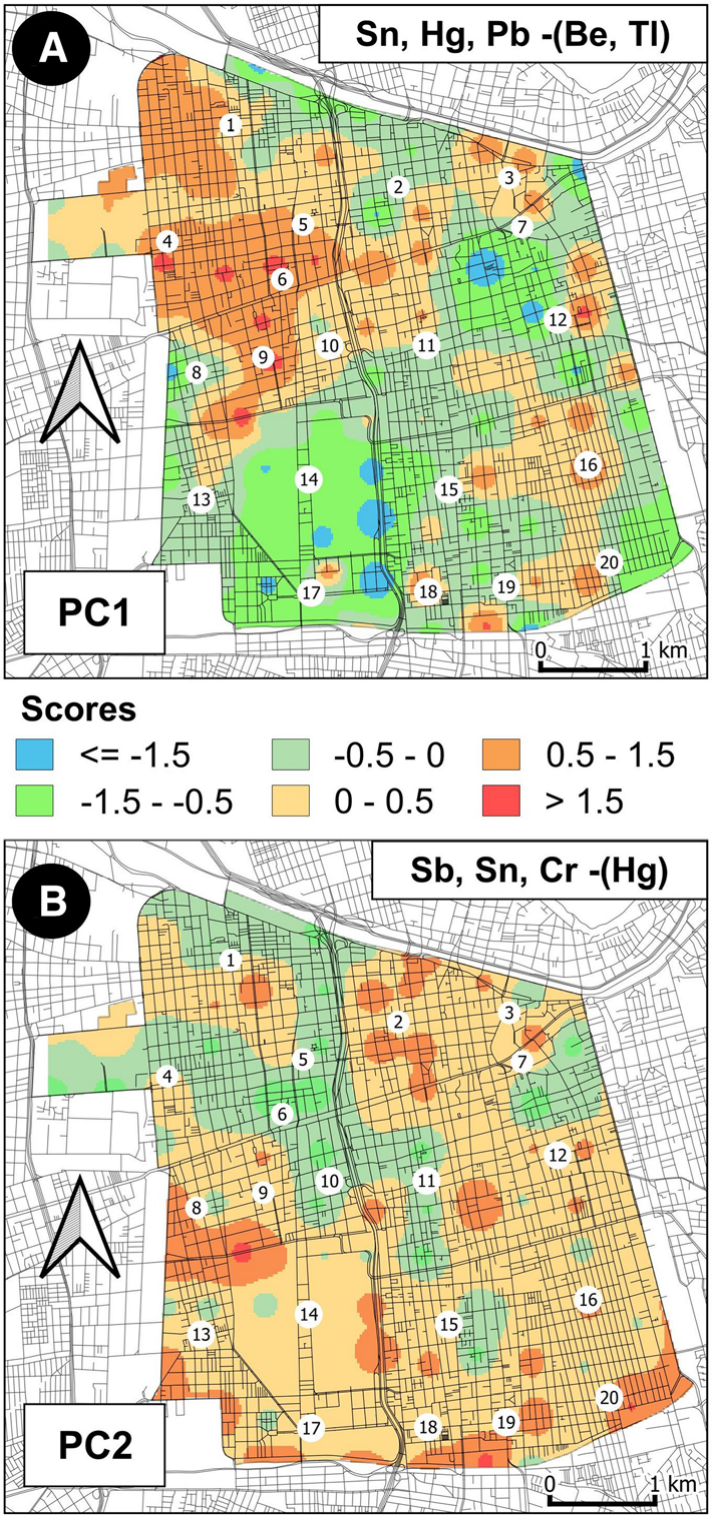
MIDW interpolated maps of scores assigned to observations (samples) related to PC1 (**A**) and PC2 (**B**). Numbers on maps refer to neighborhoods of the Commune as reported in caption of Fig. 2

For each sample in the dataset, the scores relative to both PC1 and PC2 were extracted from the PCA results. Scores were subsequently assigned to their sample coordinates and MIDW interpolated maps were generated (Fig. 7A, B). Intervals on the maps were assigned based on a symmetrical principle, taking into consideration that the contribution of the sample to a component becomes irrelevant when its score is close to a value of 0.

#### Sequential binary partition and balances

Based on the robust PCA results, the PTEs contributing to the first components (i.e. Sn, Hg, Pb, Be and Tl) (Figs. Fig. 5B, 7A) were used as input for a sequential binary partition (SBP) (Table 2).

**Table 2:** The SBP matrix used to develop the coordinates of the 4 balances

Order	SBP					Balance	Mean	Variance
	Be	Tl	Hg	Pb	Sn			
#1	−1	−1	1	1	1	b1	3.33	1.06
#2	0	0	1	−1	−1	b2	−3.98	0.32
#3	0	0	0	1	−1	b3	1.61	0.17
#4	1	−1	0	0	0	b4	0.94	0.19

For the SBP, variables were assigned different signs (+ 1 or −1) and different orders of partition considering:

1. The sign of their loadings on the PC1 axis (Order #1);
2. The degree of correlation between vectors (variables) as criteria for grouping or contrasting variables into higher orders (Order #2 to #5).

The SBP was used as the orthonormal basis for developing balances between elements, splitting the considered composition into non-overlapping groups.

Balances have a peculiar type of ilr coordinates based on the contrast between groups of elements and are calculated using Eq. 4:

4 $$b_{i} = \sqrt {\frac{{\left| i \right|\left| {i_{n} } \right|}}{{\left| {i_{p} } \right| + \left| {i_{n} } \right|}}} \log \left[ {\frac{{g(i_{p} )}}{{g(i_{n} )}}} \right]$$

where

- *b*_*i*_*,* with *i* varying among 1 and D-1, is the i-esim balance
- | i_p_ | and | i_n_| are the norms (or lengths) of the sub-compositions of positively-balanced and negatively-balanced components, respectively.
- *g(i*_*p*_*)* and *g(i*_*n*_*)* are the geometric mean of the sub-compositions.

Following the SPB matrix, four balances were determined and the corresponding ilr coordinates were calculated for each sample in the dataset (Table 2).

Specifically,

- The first balance (b1) was based on the log-contrast between Hg, Sn, Pb, Be and Tl;
- The second balance (b2) was based on the log-contrast between Hg, Sn and Pb;
- The third balance (b3) was based on the log-contrast between Pb and Sn;
- The fourth balance (b4) was based on the log-contrast between Be and Tl.

In general, the ilr coordinates assume that values range from negative to positive; the more positive a value, the more predominant the numerator variables are over the denominator variables and vice versa. However, if all of the signs are positive for the ilr coordinates of a balance, then the eventual decrease of the values reflects a progressive increase in the weight of the variables in the denominator. Similarly, the progression of values to zero for negative ilr coordinates highlight a relative increase in the relevance of the numerator variables versus the denominator variables.

**Fig. 8:**
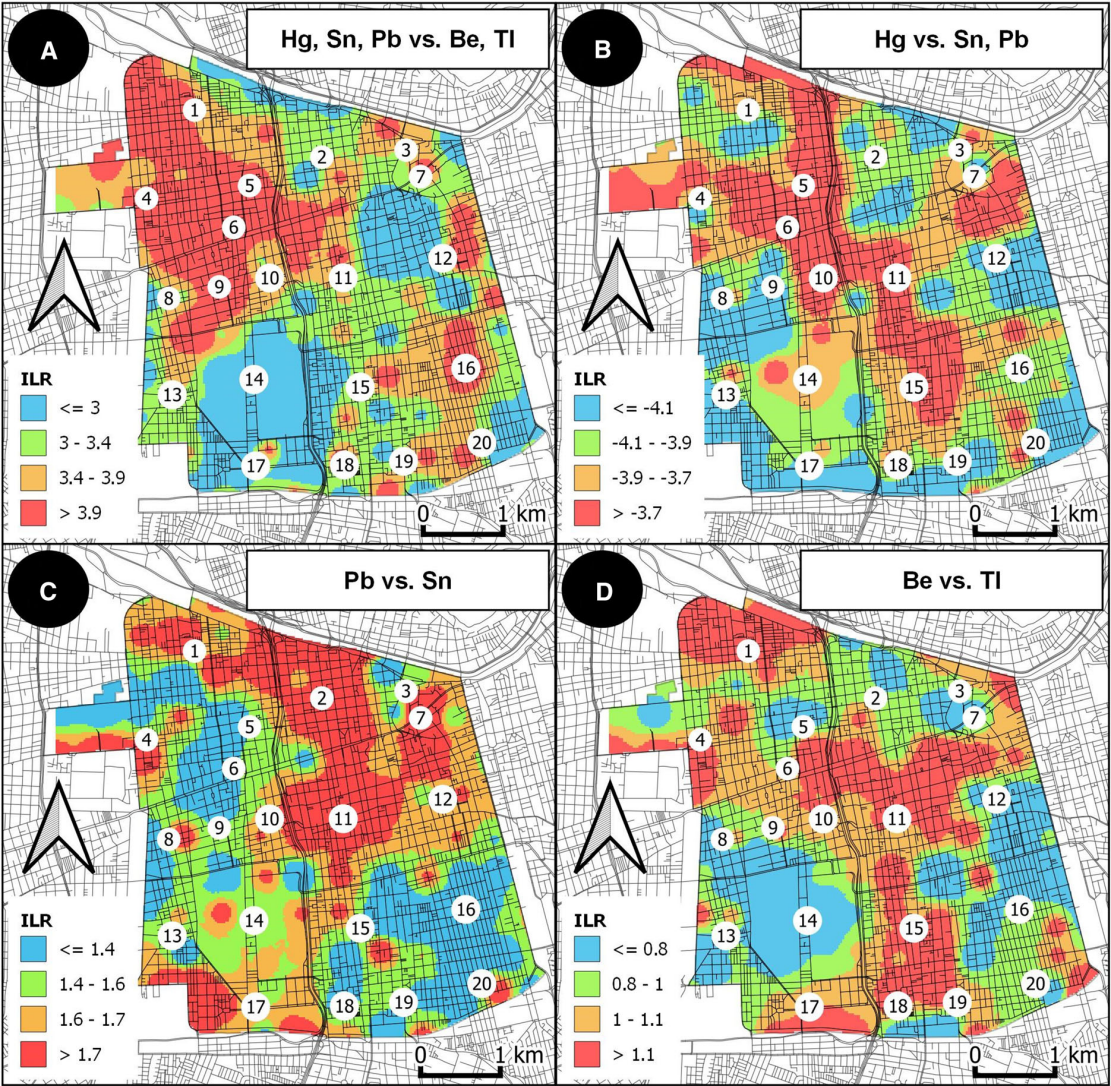
MIDW interpolated maps of ILR coordinates of balances determined on the basis of the SBP. Numbers on maps refer to neighborhoods of the Commune as reported in caption of Fig. 2

MIDW maps were generated for each of the four balances using ilr coordinates to visualize the spatial distribution of the log-contrasts (Fig. 8).

### Stochastic risk assessment

The intensity of the adverse effects of human exposure to chemicals in contaminated environmental matrices is usually determined according to a widely established human health risk assessment (RA) (National Research Council–NCR, 1983). This method takes into account the potential daily uptake of hazardous chemicals by a human receptor (defined as “total daily intake” or “dose”) and the toxicological characteristics of the same substance. Toxicity is normally expressed using a reference dose (RfD) and/or slope factor (SF), depending on the non-carcinogenic and/or carcinogenic effects that can be induced by individual contaminants and potentially absorbed by human receptors.

Risk assessment is usually performed for different exposure pathways (e.g., inhalation, ingestion, skin contact, etc.) chosen in accordance with a previously established conceptual model depicting the way contaminants reach receptors.

The general equation to assess the dose (D) (expressed as mg kg^−1^ day^−1^) from a generic medium and generic exposure pathway for a single pollutant is:

5 $$D = \frac{C \times IR \times AF \times EF}{{BW}}$$

where

- C = Contaminant concentration (Expressed as mg kg^−1^)
- IR = Intake rate of contaminated medium (soil, water, etc.) (Expressed as kg day^−1^)
- AF = Bioavailability factor
- EF = Exposure factor
- BW = Body weight (Expressed as kg^−1^)

The bioavailability factor (AF), which represents the effective fraction of the total amount of contaminant able to enter the bloodstream (i.e. the bioaccessible fraction), is normally set to 1 (100%) for screening purposes—i.e., all of the substance that a person is exposed to is assumed to be absorbed. In detailed studies, AF can be assessed trough in vitro (or in vivo*)* tests based on the simulation of the action of digestion (saliva, gastric and intestinal juice) or pulmonary fluids on samples of a contaminated matrix (for example, soil). The use of AF based on tests could, obviously, bring to results less conservative but more representative of the real risks to which human beings are exposed (Zingaretti & Baciocchi, 2021).

The exposure factor (EF) represents the fraction of the entire exposure period that effectively corresponds to the time spent in contact with the contaminated medium by the receptor. It can be calculated as an average dose over the exposure interval through the equation:

6 $$EF = \frac{F \times ED}{{AT}}$$

where

- F = Frequency of exposure (Expressed as days year^−1^)
- ED = Exposure duration (Expressed as years)
- AT = Averaging time (given by ED × 365 days year^−1^)

Depending on the specific conceptual model developed for a given area, Eq. 5 can be adapted to determine the daily intake by receptors through selected exposure pathways (Supplementary material S4).

Finally, the risk due to exposure to contaminants with non-carcinogenic and/or carcinogenic effects (USDOE, 2011; USEPA, 1989, 1997, 2001), expressed as the hazard quotient (HQ) and incremental lifetime cancer risk (ILCR), respectively, is assessed through the general equations:

7 $$HQ = \frac{D}{RfD}$$

and

8 $$ILCR = D \times SF$$

where

- RfD = Reference dose for the onset of non-carcinogenic effects (Expressed as mg kg^−1^ day^−1^) for a specific contaminant
- SF = Cancer slope factor (expressed as mg kg^−1^ day^−1^) for a specific contaminant

In the case of a risk assessment of the inhalation of contaminated dusts, RfD in Eq. 7 is replaced by the reference concentration (RfC) (Expressed as mg m^−3^) and SF in Eq. 8 is replaced by the inhalation unit risk (IUR) factor (Expressed as m^3^ μg^−1^).

Supplementary material S5 includes the toxicological values (where available) for PTEs of interest for both ingestion and inhalation pathways.

For an individual pathway, each single non-carcinogenic contaminant with HQ < 0.2 (USEPA, 1989, 2005) is considered to not be relevant for risk assessment.

As for the same pathway, the HQs of contaminants (with values ≥ 0.2) producing potentially adverse effects on the same target organ (Supplementary material S6) have to be added together to calculate the hazard index (HI) (USEPA, 1989, 2005) as follows:

9 $$HI = \sum HQ_{i}$$

where

- HQ_i_ is the hazard quotient of the i-esim contaminant (with values of HQ ≥ 0.2)

An HI value ≥ 1 (USEPA, 1989, 2005) implies that the risk cannot be considered acceptable for the target organ.

In the case that only one contaminant is characterized for a specific target organ with HQ ≥ 0.2, the HQ will be assimilated with the HI and the target organ will only be considered at risk if HQ ≥ 1. A graphical summary of the most likely scenarios is shown in supplementary material S7.

Obviously, for a target organ that can potentially be affected by one or more contaminants, we can have as many HIs as potential exposure pathways considered.

ILCR values between 10^–6^ and 10^–4^ are considered tolerable (USEPA, 2011). Values below 10^–6^ indicate no risk, while values above this threshold are considered unacceptable by most of the international regulatory agencies and health organizations (USEPA, 1989; WHO, 2001).

For carcinogenic effects, the risk is assessed for each contaminant and pathway. Target organs are not considered in any assessment procedure and knowledge about them only provides additional information about the potential effects of contaminants on a population.

In general, RA procedures are designed for site-specific purposes and are based on a deterministic approach, where a statistical-based representative value is used (e.g., upper or lower confidence limit established from available observations) for each single variable involved in the calculations.

However, in a broader context such as an urban area, the need to consider the uncertainty related to the spatial variability of population characteristics, exposure timing, and pollutant concentrations warrants a stochastic approach. Basically, a probabilistic (stochastic) risk assessment should rely on the same equations used by deterministic procedures but variables should be expressed as statistical distributions.

Based on the considerations above, the Monte Carlo simulation approach has been chosen as the computational method to run a probabilistic assessment of human health risk for the Commune of Santiago for both non-carcinogenic and carcinogenic effects of PTEs (Burmaster & Anderson, 1994; U.S. EPA, 1997). The simulation was performed using the Oracle Crystal Ball software. The assessment was performed by grouping receptors into two separate age ranges (i.e. children and adults) and two specific pathways of exposure (i.e. accidental or non-accidental ingestion of soil and inhalation of dust rising from the ground) in a residential setting (Supplementary material S3). The ingestion pathway was considered to assess the risks posed only by children due to their widely recognized predisposition for geophagy (Moya & Phillips, 2014).

For each geochemical variable (i.e. PTEs concentration data), the method generated a series of simulated dose (D) values following a probability distribution that the variable itself was expected to have according to available observations. As a consequence, the risk assessment results were returned not as unique values of risk but as distributions of HI or ILCR values following a significant number of iterations (e.g., 50,000 with a confidence level of 95%) applied to Eqs. 7, 8 and 9 (Supplementary material S8).

In the case of non-carcinogenic adverse effects, the results of the assessment are expressed as a certainty (probability) that HQ could exceed (or at least equal) the threshold value of 0.2 when considering a specific age group (children or adults), pathway or element (Table 4). Elements with the probability (even if minimal) to overcome (or be equal to) the HQ threshold were included in a further estimate of the certainty of overcoming (or equaling) the HI threshold of 1 for the targeted organ/system (Table 5).

For carcinogenic effects, the risk is also reported as a percentage of certainty for a person belonging to a specific age group who could be assigned a non-acceptable value of risk (ILCR > 10^–6^) based both on a specific pathway and specific contaminant (Table 6).

## Results and discussion

### Geochemical patterns

When comparing the median concentration values of PTEs in topsoils from the study area with values from other highly urbanized cities around the world (Table 3), Cu, Mo, and, to a lesser extent, Zn tend to be more strongly enriched in Santiago. Since Cu concentrations are an order of magnitude higher than those recorded in other cities, it is plausible that Cu enrichment (and subordinately Mo) could be associated with the deposition of dust carried by atmospheric currents flowing from exploitation areas containing porphyry copper deposits not far from the city (El Teniente, Los Bronces, Andina, etc.) (Camus, 2005; Crespo et al., 2018). It is also worth noting that concentrations of As, V, and Zn are higher than most of the considered world cities. On the other hand, our data show that concentrations of Cd, Co, Cr, and Ni are in line with world averages while concentrations of Be, Hg, and Tl are below average.

**Table 3:** Comparison of median concentrations of potentially toxic elements in some world cities with a long history of urbanization

City	As	Be	Cd	Co	Cr	Cu	Hg	Mo	Ni
Mexico City	–	–	–	–	116	54	–	–	39
Hong Kong (China)	–	–	0.52	3.02	21.6	16	–	–	11.2
Sicily (Italy)	–	–	0.68	5.2	34	63	0.68	–	17.8
Chicago (USA)	13.2	2	–	11	65	59	0.19	5	31
Lisbona (Portugal)	4.4		–	6.8	16	29	0.18	0.6	20
Napoli (Italy)	11.9		0.37	6.3	11.2	74	0.18	1.17	8.9
Beijing (China)	–	–	0.11	–	60	26.1	–	–	23.8
Bristol (UK)	21.7	–	1.1	–	23.1	60.1	–	–	21
Athens (Greece)	24	–	0.3	16	141	39	–	–	102
Oslo (Norway)	4.5	5.54	0.34	9.74	28.5	25.5	0.06	1.31	24.1
Sevilla (Spain)	–	–	–	–	42	41.7	–	–	23.1
Annaba (Algeria)	–	–	0.3	22.6	23.8	23.8	–	–	–
Baltimore (USA)	–	–	0.89	12.1	38.3	35.2	–	–	18.4
Berlin (Germany)	3.9	1.2	0.35	–	25.1	31.2	0.19	–	7.7
Damascus (Syria)	–	–	–	10	51	30	–	–	35
Galway (Ireland)	8	–	–	6	35	27	–	–	22
Trondheim (Norway	3.3	–	0.19	–	65	39	0.15	–	45
Ibadan (Nigeria)	3	–	0.15	–	55.5	32	–	1.4	16.5
Santiago (Chile)	14.1	0.4	0.42	14.1	21.9	188.87	0.02	2.83	13.6

City	Pb	Sb	Sn	Tl	V	Zn	Analytical Method	References
Mexico City	82	–	–	–	97	219	XRF, HClO_4_+ HF	Morton–Bermea et al. (2009)
Hong Kong (China)	77.2	–	–	–		92.1	HNO_3_ + HClO_4_	Li et al. (2004)
Sicily (Italy)	202	3	–	–	54	138	HNO_3_ + HCl	Manta et al. (2002)
Chicago (USA)	198	–	–	–	82	235	HClO_4_ + H_2_SO_4_ + HF + HCl	Cannon and Horton (2009)
Lisbona (Portugal)	62	0.7	–	–	27	88	HNO_3_+ HCl	Cachada et al. (2013)
Napoli (Italy)	141	2		1.06	52	158	HNO_3_+ HCl	Cicchella et al. (2008)
Beijing (China)	19.3	–	–	–	–	84.5	HNO_3_ + HCl + HClO_4_ + HF	Wang et al. (2012)
Bristol (UK)	210.1	–	–	–	–	272.6	HNO_3_+ HCl	Giusti (2011)
Athens (Greece)	45	1.7	3.6	–	–	98	HNO_3_ + HCl + HClO_4_ + HF	Argyraki–Kelepertzis (2014)
Oslo (Norway)	33.9	–	–	–	51.9	130	HNO_3_	Tijhuis et al. (2002)
Sevilla (Spain)	103	–	–	–	–	86	HNO_3_+ HCl	Madrid et al. (2004)
Annaba (Algeria)	42.3	–	–	–	–	64.7	HNO_3_	Maas et al. (2010)
Baltimore (USA)	89.3	–	–	–	31.4	80.7	HNO_3_ + H_2_O_2_ + HCl	Yesilonis et al. (2008)
Berlin (Germany)	76.6	–	6	–	–	129	XRF, HNO_3_ + HCl	Birke and Rauch (2000)
Damascus (Syria)	10	–	–	–	–	84	HNO_3_+ HCl	Möller et al. (2005)
Galway (Ireland)	58	–	–	–	50	85	HNO_3_ + HCl + HClO_4_ + HF	Zhang (2006)
Trondheim (Norway	81	–	–	–	–	112	HNO_3_	Andersson et al. (2010)
Ibadan (Nigeria)	47	–	–	–	–	93.5	HNO_3_ + HCl	Odewande and Abimbola (2008)
Santiago (Chile)	68.92	1.61	7.2	0.11	94	227	HNO_3_+ HCl	This study

The values are in mg/kg

Among all the elements analyzed (Table 1), Cu, Pb, Zn, Hg, and subordinately Sn and V, have the strongest median absolute deviation (M.A.D.) and variance in Santiago soils. However, since these latter statistical parameters are directly influenced by the units and magnitude of the data, the coefficient of variation (CV) was deemed more appropriate to assess the inner variability of each element and to compare the variation of different variables characterized by median values as significantly different. Considering all the PTEs, Pb, Sb, and Sn are the elements with the highest dispersion followed by Cu, Cr, and Zn; V and Co are the least variable elements.

The primarily positive skewness values (with the exception of As) reveal a general tendency of the data to be asymmetric (tailed toward the right) with most of the distributions from moderately (Zn < Mo < Cd < Sn < Pb) to strongly peaked (Cu < Sb < Ni <  < Hg) elements staying in accordance with increasing values of kurtosis, which are often well above 3, a value considered to be a reference for the state of normality of distribution (Cullen & Frey, 1999) (Supplementary material S9).

All the above considerations and the direct observations inferred from both the EDA plots (Supplementary material S2) and the skewness-kurtosis graphs (Supplementary material S9) suggest that most elements have multiple populations in their distributions, and hence the potential contribution from multiple sources.

According to soil pH values (varying from 5.03 to 9.29 with an average of 7.78), about 80% of the samples collected in the study area are from weakly to moderately alkaline soils (Table 1) (U.S. Department of Agriculture, 1993). The range in variation of pH reflects the potential influence of anthropogenic activities on natural settings: the introduction of soils to small fractions of building materials and atmospheric fallout of dust associated with industrial activity and urban mobility can cause carbonation (Howard & Orlicki, 2015) or acidification of soils (Ulrich, 1986), respectively.

The individual spatial distributions of all of the considered PTEs (Figs. 2 and 3) have highly irregular patterns, making it very difficult to determine the potential point sources to explain elemental enrichments.

The dominance of high concentrations in almost all the analyzed elements in the neighborhoods of Yungay, Concha y Toro, Brasil, Republica, and subordinately Centro Historico, suggests that one of the main sources of contamination may be motor vehicle traffic. In fact, these areas represent the core of urban development of the city and are the main areas subjected to anthropogenic activity. Additionally, most of the anomalously high concentrations are found along two of the main urban roads that cross the Commune –southwest to northeast (Avenida Libertador Bernardo O'Higgins) and north to south (Avenida Manuel Rodriguez Norte).

With regard to Tl, it is worth noting that the highest concentration values of this element mark most of the urban green areas (e.g. Parque O' Higgins, the green area falling within the neighborhood of Parque Club) where, probably, the less degree of anthropogenic alteration, allow soil to show the original fingerprint of their volcanoclastic origin.

An in-depth observation of the traffic flow (Supplementary material S10) shows that generally the highest values of the Cumulative Contamination Degree (CCD) fit well with all of the sectors of the local road network, which are normally characterized by the slowest flow of traffic resulting in the highest levels of congestion, especially at rush hours.

In fact, it is interesting to point out that among the contaminants considered to be relevant for the first two principal components are Pb, reflecting the historical use of leaded gasoline (Albanese & Cicchella, 2012), Sb and Sn, which have been associated (together with Cd and Zn) with motor vehicle non-exhaust emissions (Adamiec et al., 2016) such as tires (Councell et al., 2004) and brake pad consumption (Grigoratos & Martini, 2015; Sathickbasha et al., 2019). Furthermore, the PCA biplot (Fig. 6) which corresponds with the vectors representing the above elements, shows that the samples with the highest positive scores also have the highest CCD values.

When considered in detail, PC1 is a component that potentially represents an association with elements related to historical contamination sources, in contrast with the main geochemical features of soils in green areas that are less impacted by human activity (Fig. 1). In fact, the highest positive values of the component are concentrated in the northwestern sector of the Commune, the historical center (Centro Historico) and other critical points along the road network (Fig. 7A). On the other hand, the negative scores are mainly associated with Be and Tl (related to the presence of alkali volcanics) that are primarily found in green areas (Parque O’ Higgins, Club Hipico, etc.) where volcanic soils are probably influenced to a lesser extent by the atmospheric fallout of contaminants.

PC2 allows for the opportunity to potentially discriminate between different sources of anthropogenic pollutants. In fact, positive scores mostly associated with Sb, Sn, and Cr are spread out across most of the Commune territory except in the northwestern sector of the study area where negative scores are associated with Hg (Fig. 7B).

In agreement with Pérez et al. (2019) and also confirmed by the single elemental distribution (Fig. 2), the different behavior of Hg can be associated with the presence of two power plants fuelled by fossil fuels (coal, diesel, gas) and located just beyond the north western boundary of the study area: (1) the “Renca Thermal Power Plant” (http://globalenergyobservatory.org/geoid/44667), lacking technologies to control particulate matter (PM) emissions and exclusively fueled by coal since 1962 (before recently shifting to diesel fuel), and (2) the “Nueva Renca CCGT Power Plant” (http://globalenergyobservatory.org/geoid/44611), commissioned in 1998 and fuelled by natural gas and most recently diesel fuel.

Clearly, the spatial pattern exhibited by scores in PC1 (Fig. 7A) is confirmed by the MIDW map of ILR coordinates (Fig. 8A) based on the balance (b1) associated with the first order of the SPB (Table 2). In fact, Fig. 8A shows the effect of the log-contrast among anthropogenic (Hg, Pb, Sn) and geogenic (Be and Tl) elements, with the highest values of coordinates resembling the pattern of positive scores of the first component of the PCA.

The map of coordinates of the second order balance (b2) (Fig. 8B), which put Hg in contrast with Sb and Pb, likely highlights the areas of the Commune where the influence of the fallout of particulate matter deriving from the thermoelectric plants of Renca (marked by high Hg content) is prevalent compared to the historical contamination associated with motor vehicle exhaust (Pb) and non-exhaust emissions (Sn). Incoherence with the pattern of ILR, the presence of crematoria ovens within the neighborhood of Parque Club and beyond the northeastern border of the Commune cannot be ruled out as additional potential point sources of Hg (González-Cardoso et al., 2020).

The relationship between Pb and Sn has been evaluated through a further balance (b3) (Fig. 8C), although there is small variability in the data that supports only a limited part (Table 2) of the total variance. Specifically, the highest values of the ILR coordinates (corresponding to a prevalence of Pb over Sn) could be interpreted as potential markers for areas historically affected by both intense motor vehicle traffic normally associated with high exhaust emissions (Muzychenko et al., 2017) and limited air circulation due to the presence of street canyons. Lower values of ILR could also be used to discriminate soils collected in residential areas that are crossed by numerous secondary roads (with little traffic) and intersections requiring high braking frequency yielding the production of non-exhaust emissions.

The ILR coordinates associated with the fourth order of SPB (Table 2), which are also representative of a balance (b4) (Fig. 8D) and have little influence on the total variability of the data, could be useful to discriminate areas of the Commune where secondary anthropogenic contributions of Be are associated with fossil fuels in an urban context (Taylor et al., 2003).

### Potential health impacts

Among all of the PTEs with toxicological reference values available for non-carcinogenic effects (Supplementary material S3), only the ingestion pathway (limited to children) shows a number of elements having a probability (certainty > 0%) of overcoming the HQ threshold of 0.2. Specifically, the percentage of certainty (Table 4) has the following decreasing order: V≃As≃Co >  > Pb >  > Sb > Cr_tot_ > Cu > Hg.

**Table 4:** Percent of certainty for HQ to equal or exceed the reference threshold for individual PTEs

HQ ≥ 0.2	Certainty		
	Ingestion	Inhalation	
Element	Child (%)	Child (%)	Adult (%)
As	**99.99 **	0	0
Be	0	0	0
Cd	0	0	0
Co	**98.86 **	0	0
Cr_Tot_	**2.44 **	–	–
Cu	**1.30 **	–	–
Hg	**0.001 **	0	0
Mo	0	0	0
Ni	0	0	0
Pb	**51.18 **	–	–
Sb	**3.88 **	0	0
Sn	0	–	–
V	**100 **	–	–
Zn	0	–	–

Values of certainty above 0 are reported in bold characters

Based on percentage values, PTEs can roughly be separated into three groups:

- V, As and Co characterized by values of certainty close to 100% with maximum values of HQ equal to 2.05, 1.24 and 1.31, respectively, with an almost symmetrical distribution of all of these elements (Supplementary material S5);
- Pb with a certainty of 51.18% and a distribution strongly skewed towards the right with a maximum value of HQ of 11.1 (Supplementary material S5);
- Sb, Cr_tot_, Cu, and Hg with a very low probability of overcoming the HQ threshold and a general tendency to be asymmetrical and skewed towards the right (Supplementary material S5).

By considering all of the above elements, the distribution of the hazard index (HI) for specific target organs following ingestion were determined for children (Table 5).

**Table 5:** The percentage of certainty for HI to equal or exceed the reference threshold for specific target organs/systems

HI ≥ 1	Organ/System	Elements	Certainty (%)
Child	Central nervous system	Hg and Pb	**3.780 **
	Kidney	Cu, Hg, Pb and V	**83.950 **
	Gastrointestinal system	Cu and V	**44.250 **
	Cardiovascoular system	As and Pb	**50.810 **
	Red blood cells	Pb	**3.622 **
	Blood	V	**34.226 **
	Development	Cu	**0.002 **
	Reproduction		
	Haematopoietic system		
	Liver		
	Gastrointestinal Tract	Sb	**0.029 **
	Skin	As	**4.465 **
	Nervous system		
	Not Assessed (NA)	Cr_tot_	0.000

Values of certainty above 0 are reported in bold characters

Organs that have a relevant probability of being affected by a non-acceptable hazard are, in decreasing order, kidneys (affected by Cu, Hg, Pb, and V), the cardiovascular system (affected by As and Pb), the gastrointestinal system (affected by Cu and V) and the blood (affected by V only).

The distribution of HI values for kidneys (with a certainty of overcoming the HI threshold of 83.95%) shows a slight skewness towards the right with a median value of 1.24 and a maximum value of 12.1, highlighting the risk to young people. Similar to the kidney risk, the hazard distribution for cardiovascular systems of children (with a certainty of 50.8%) is also clear, with a maximum value of 11.7 compared to a median of about 1.

More organs show a probability of HI > 1, but the certainty of overcoming the reference threshold varies from low (e.g., skin and nervous system are affected by As with a certainty of 4.47%) to very low percent values.

Total Cr, even though it has no target organ, is the only element among the ones discussed above with an individual distribution that does not overcome the HI value of 1.

The carcinogenic risk was assessed for both ingestion and inhalation pathways (Table 6). Ingestion was assessed for children by determining the ILCR distributions depending on As and Pb according to the available toxicological values (Supplementary material S5). The distribution of ILCR for As has a certainty of 100% to overcome the selected reference threshold of 10^–6^ with a maximum value of 4.27 × 10^–5^. The distribution of carcinogenic risk for Pb also shows a partial overcoming of the acceptable threshold with a considerable certainty value of 18.63% and a maximum ILCR value of 2.81 × 10^–5^.

**Table 6:** Percent of certainty for ILCR to equal or exceed the reference threshold for individual PTEs

ILCR ≥ 10^–6^	Certainty		
	Ingestion	Inhalation	
Element	Child (%)	Child (%)	Adult (%)
As	**100**	0	0
Be	–	0	0
Cd	–	0	0
Co	–	0	0
Cr_Tot_	–	0	0
Ni	–	0	0
Pb	**18.63**	**9.27**	**72.03**

Values of certainty above 0 are reported in bold characters

The probability of carcinogenic risk from the inhalation of dust with Pb for both children and adults is also not acceptable. The certainty for adults is quite high (i.e. 72%) while that for children is considerably lower (~ 9%).

## Conclusions

In this preliminary study on the surficial soils of Commune of Santiago, the effort to consider both the degree and complexity of contamination to obtain a comprehensive view of the distribution of the degree of contamination at the urban scale generated significant results. In fact, the highest values of the CCD have a good correspondence with topsoil samples influenced primarily by anthropogenic processes according to PCA.

The use of multivariate analysis (PCA), performed using data transformed to take into account compositional nature to exclude spurious correlations, enabled the determination of a first discrimination of geochemical sources (natural or anthropic) to further direct detailed analysis of the data. Furthermore, the use of ILR coordinates, generated by considering the principal component influencing the widest fraction of total variability, gave the authors the chance to observe the existence of secondary contamination processes that are normally difficult to constrain.

Assuming homogeneous mobility of the resident population within the territory of the Commune, this study also assessed human health risk by using a probabilistic approach taking into consideration the uncertainty generated by the variability of elemental concentrations across the entire study area. The results highlight how adults and children are characterized using different levels of risk and how ingestion could represent an important pathway of exposure to contaminants for children, especially for non-carcinogenic adverse effects to some organs such as kidneys. However, the results of any risk assessment are partial (i.e. limited to a selection of contaminants and to a specific conceptual model) and therefore represent a potentially fallible forecast, especially if the surrounding conditions change for better or worse.

Although this work is based on both a specific matrix (topsoil) and a group of contaminants (i.e. PTEs), the applied procedure could easily be extended to other media (bottom soil, water, air, etc.) to generate a more comprehensive analysis of environmental conditions in an urban setting including related risks.

## Supplementary Information

Below is the link to the electronic supplementary material.

Supplementary file1 (PDF 23 kb)

Supplementary file2 (PDF 319 kb)

Supplementary file3 (PDF 102 kb)

Supplementary file4 (PDF 1444 kb)

Supplementary file5 (PDF 132 kb)

Supplementary file6 (PDF 50 kb)

Supplementary file7 (PDF 74 kb)

Supplementary file8 (PDF 107 kb)

Supplementary file9 (PDF 4726 kb)

Supplementary file10 (PDF 111 kb)
