# Supplementary material for: A new approach to assess the degree of contamination and determine sources and risks related to PTEs in an urban environment: the case study of Santiago (Chile)
Source: Environ Geochem Health. 2022 Jan 10;45(2):275–97. doi: 10.1007/s10653-021-01185-6 (PMC9884654; doi:10.1007/s10653-021-01185-6)
Supplement: Supplementary file 1 — Supplementary file1 (PDF 23 kb) [file 10653_2021_1185_MOESM1_ESM.pdf]

A new approach to assess the degree of contamination and determine sources and risks related to PTEs in an urban environment: the case study of Santiago (Chile).

Aruta Antonio[1], Albanese Stefano[1*], Daniele Linda[2], Cannatelli Claudia[3], Buscher Jamie T.[3], De Vivo Benedetto[4,5], Petrik Attila [6], Cicchella Domenico[7], Lima Annamaria[1]

[1] *Department of Earth, Environmental and Resources Sciences, University of Naples Federico II, 80126 Naples, Italy*
[2] *Department of Geology, Andean Geothermal Center of Excellence (CEGA) and Millenium Nucleus for Metal Tracing Along Subduction, FCFM, Universidad de Chile, Plaza Ercilla 803, Santiago, Chile*
[3] *University of Alaska Anchorage, 3211 Providence Drive. Anchorage, AK 99508, USA*
[4] *Virginia Tech, Blacksburg 24061, VA, USA*
[5] *Pegaso On Line University, Piazza Trieste e Trento 48,80132 Naples, Italy*
[6] *Eriksfiord AS, Prof. Olav Hanssensvei 7A, 4021, Stavanger, Norway*
[7] *Department of Science and Technology, University of Sannio, 82100, Benevento, Italy*

**Corresponding author: stefano.albanese@unina.it*

Supplementary Material S1. Instrumental detection limits, accuracy error and relative percent difference (used to determine precision) of selected PTEs (HMTRI, 1997).

Element	U. M.	Instrumental Detection Limit (IDL)	Accuracy Error %	Relative Percent Difference (RPD) %
**As**	mg/kg	0.1	3.57	0.91
**Be**	mg/kg	1	7.14	38.52
**Cd**	mg/kg	0.01	1.53	3.08
**Co**	mg/kg	0.1	3.68	3.73
**Cr**	mg/kg	0.5	5.63	2.40
**Cu**	mg/kg	0.01	0.92	3.76
**Mo**	mg/kg	0.01	4.12	0.76
**Ni**	mg/kg	0.1	1.04	3.25
**Pb**	mg/kg	0.01	0.01	0.18
**Sb**	mg/kg	0.02	1.58	8.62
**Sn**	mg/kg	0.1	4.69	1.56
**Tl**	mg/kg	0.02	1.23	5.13
**V**	mg/kg	2	4.07	1.66
**Zn**	mg/kg	0.1	2.64	2.50
**Hg**	μg/kg	5	8.75	2.13

References

HMTRI (Hazardous Materials Training, Research Institute). (1997). Site characterization: Sampling and analysis. New York: Van Nostrand Reinhold.
