# Supplementary material for: A new approach to assess the degree of contamination and determine sources and risks related to PTEs in an urban environment: the case study of Santiago (Chile)
Source: Environ Geochem Health. 2022 Jan 10;45(2):275–97. doi: 10.1007/s10653-021-01185-6 (PMC9884654; doi:10.1007/s10653-021-01185-6)
Supplement: Supplementary file 3 — Supplementary file3 (PDF 102 kb) [file 10653_2021_1185_MOESM3_ESM.pdf]

# A new approach to assess the degree of contamination and determine sources and risks related to PTEs in an urban environment: the case study of Santiago (Chile).

Aruta Antonio[1], Albanese Stefano[1*], Daniele Linda[2], Cannatelli Claudia[3], Buscher Jamie T.[3], De Vivo Benedetto[4,5], Petrik Attila [6], Cicchella Domenico[7], Lima Annamaria[1]

[1] *Department of Earth, Environmental and Resources Sciences, University of Naples Federico II, 80126 Naples, Italy*
[2] *Department of Geology, Andean Geothermal Center of Excellence (CEGA) and Millenium Nucleus for Metal Tracing Along Subduction, FCFM, Universidad de Chile, Plaza Ercilla 803, Santiago, Chile*
[3] *University of Alaska Anchorage, 3211 Providence Drive. Anchorage, AK 99508, USA*
[4] *Virginia Tech, Blacksburg 24061, VA, USA*
[5] *Pegaso On Line University, Piazza Trieste e Trento 48,80132 Naples, Italy*
[6] *Eriksfiord AS, Prof. Olav Hanssensvei 7A, 4021, Stavanger, Norway*
[7] *Department of Science and Technology, University of Sannio, 82100, Benevento, Italy*

**Corresponding author: stefano.albanese@unina.it*

**Supplementary Material S2.** EDA (Exploratory Data Analysis) plots of selected PTEs

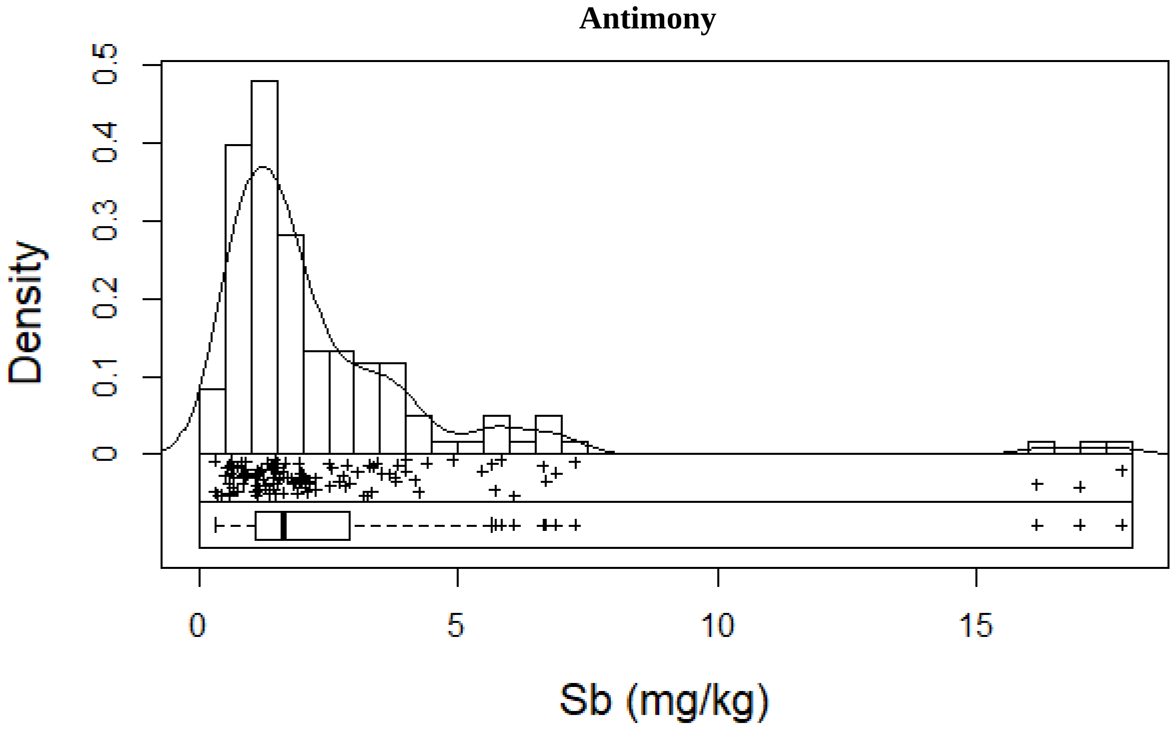


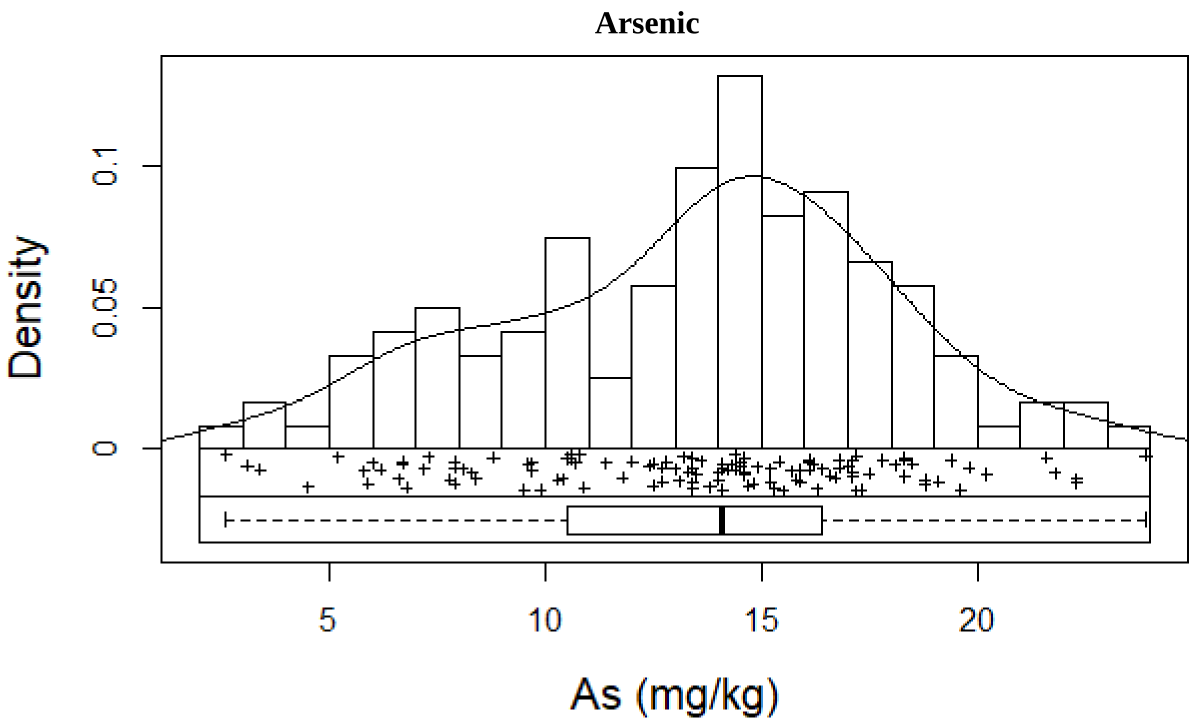

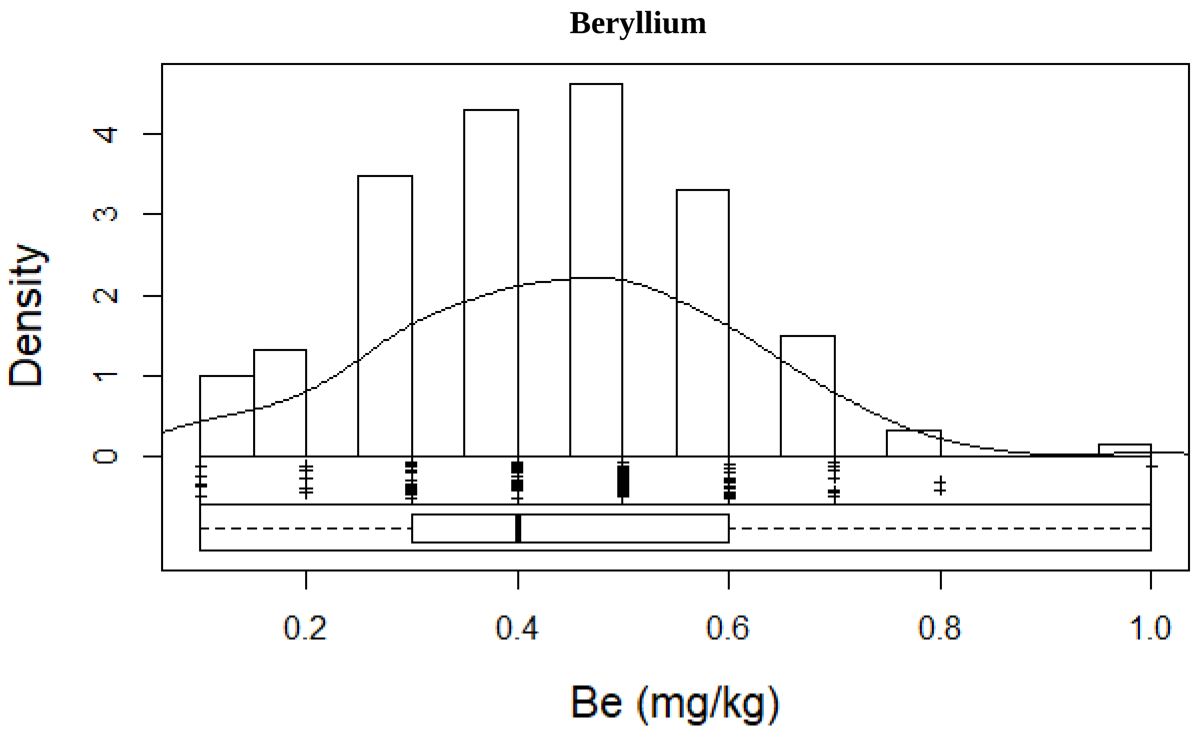

Beryllium
Density
0
1
2
3
4
0.2
0.4
0.6
0.8
1.0
Be (mg/kg)


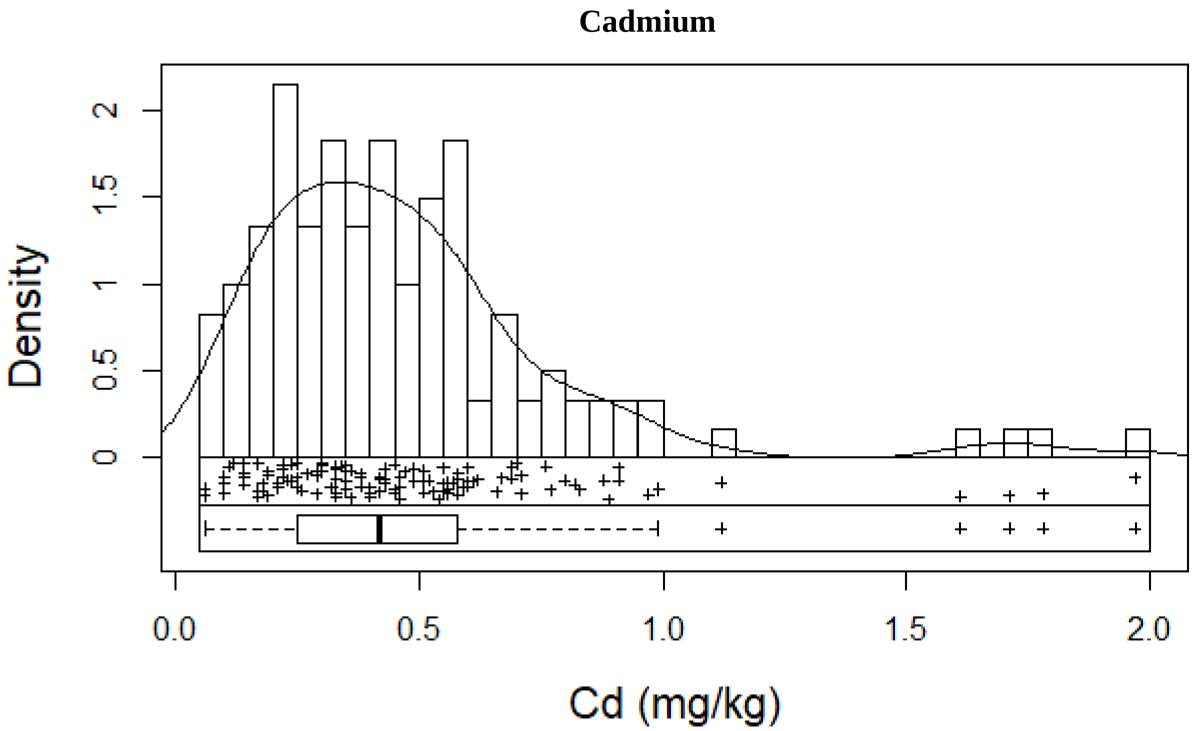

Cadmium
Density
0
0.5
1
1.5
2
0.0
0.5
1.0
1.5
2.0
Cd (mg/kg)

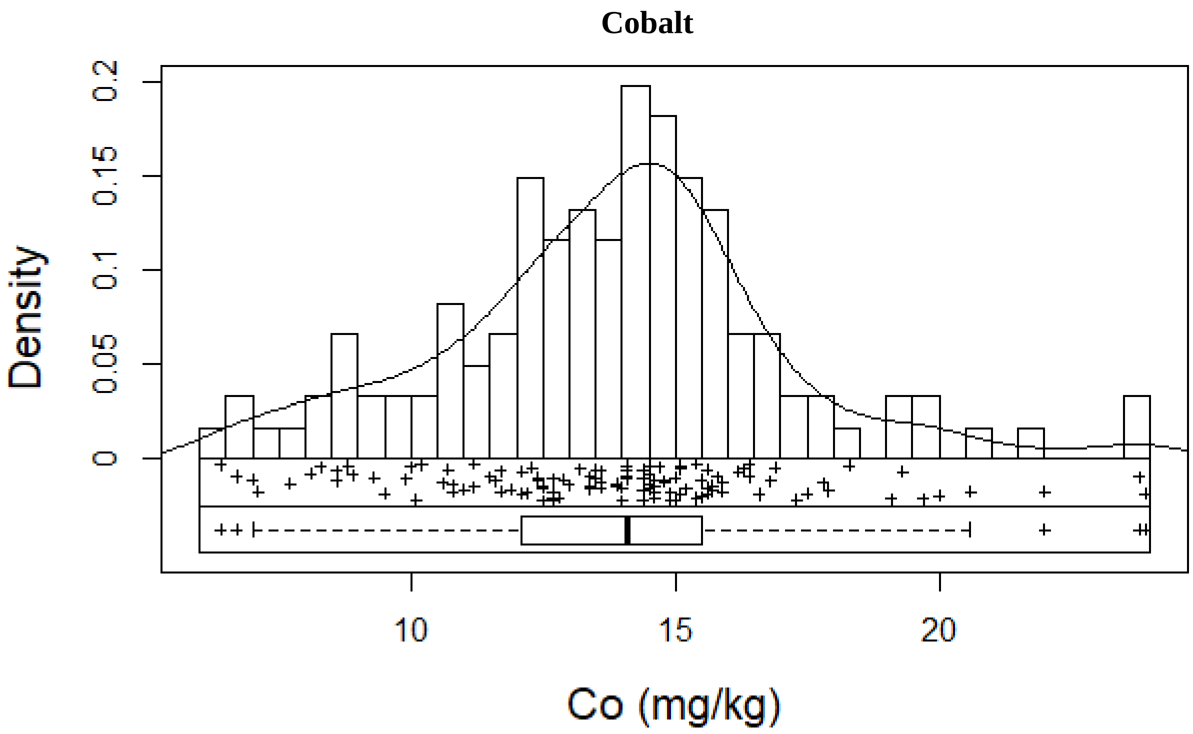

Cobalt
Density
0
0.05
0.1
0.15
0.2
10
15
20
Co (mg/kg)


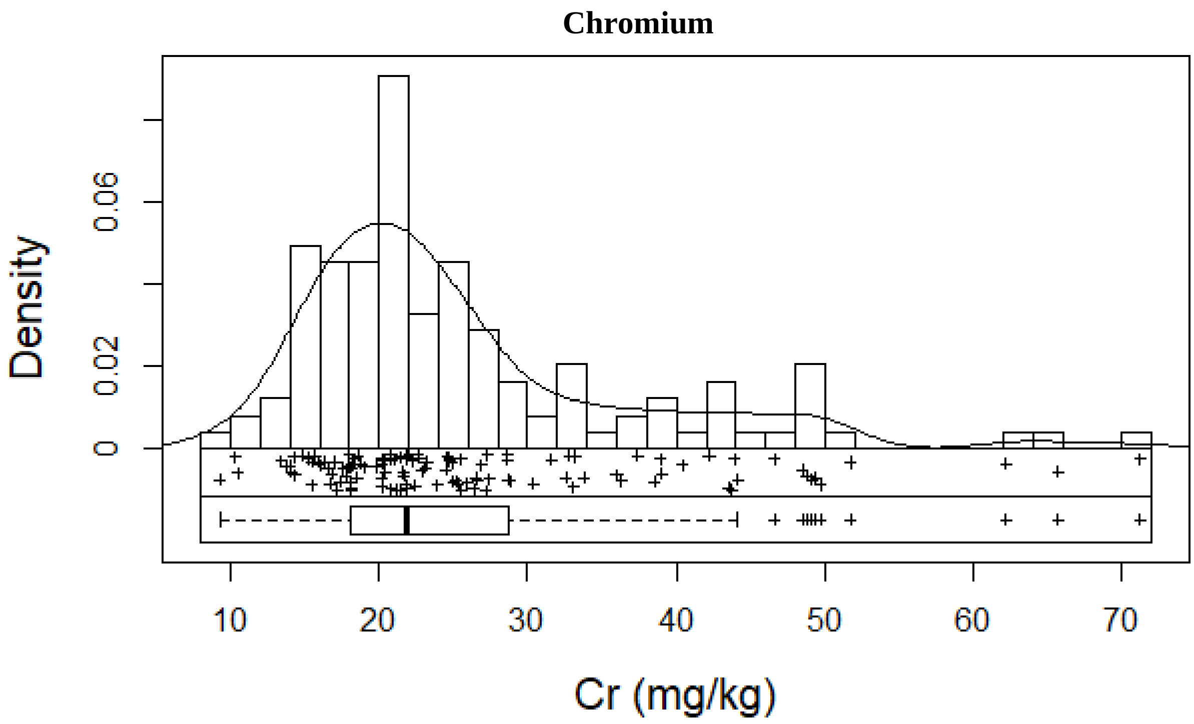

Chromium
Density
0
0.02
0.06
10
20
30
40
50
60
70
Cr (mg/kg)

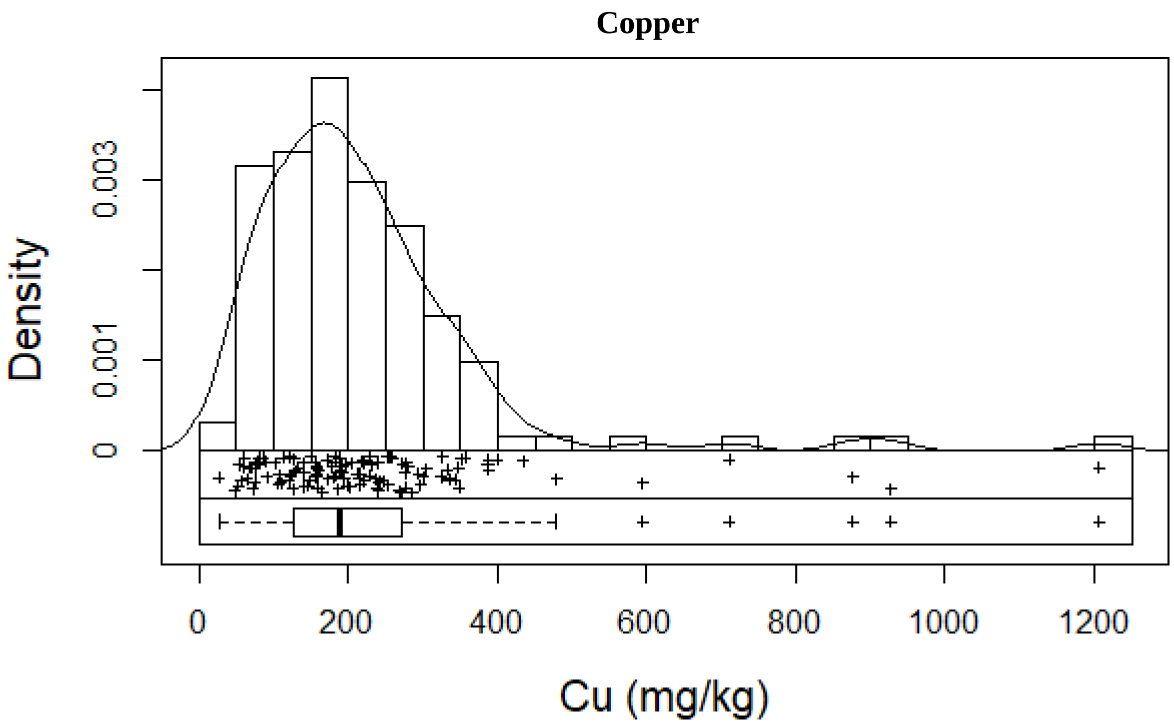
Copper
Density
0
0.001
0.003
0
200
400
600
800
1000
1200
Cu (mg/kg)

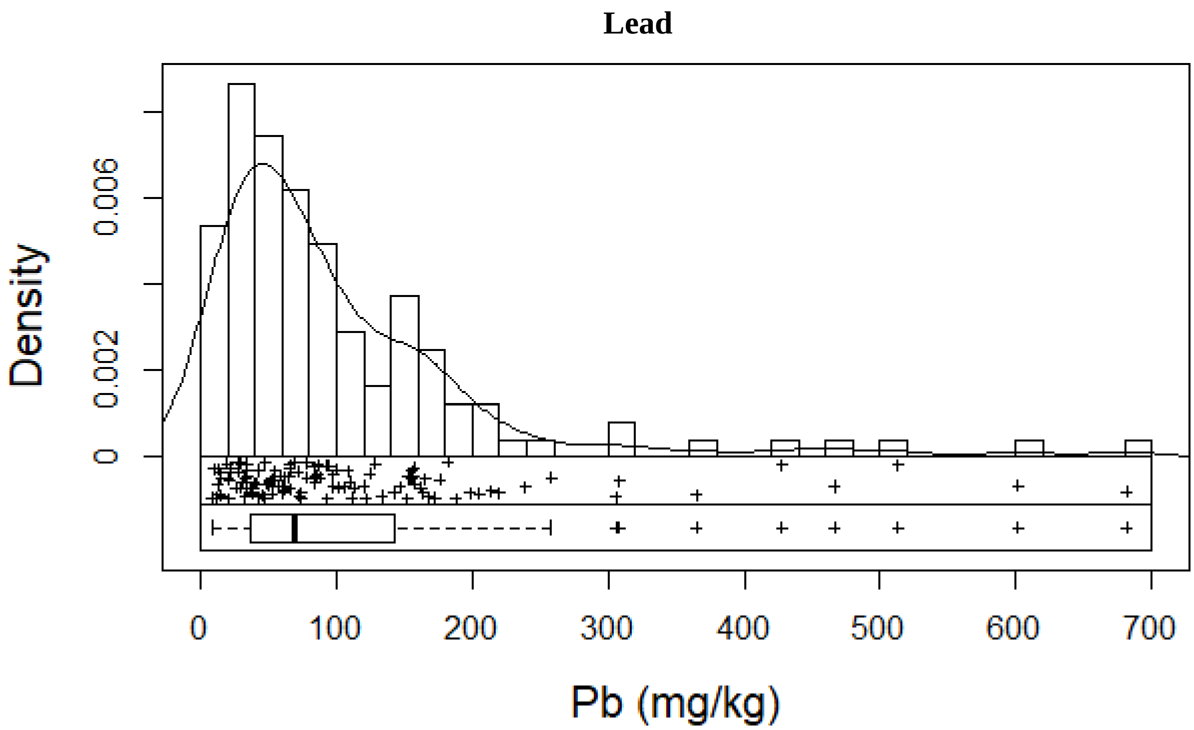
Lead
Density
0
0.002
0.006
0
100
200
300
400
500
600
700
Pb (mg/kg)

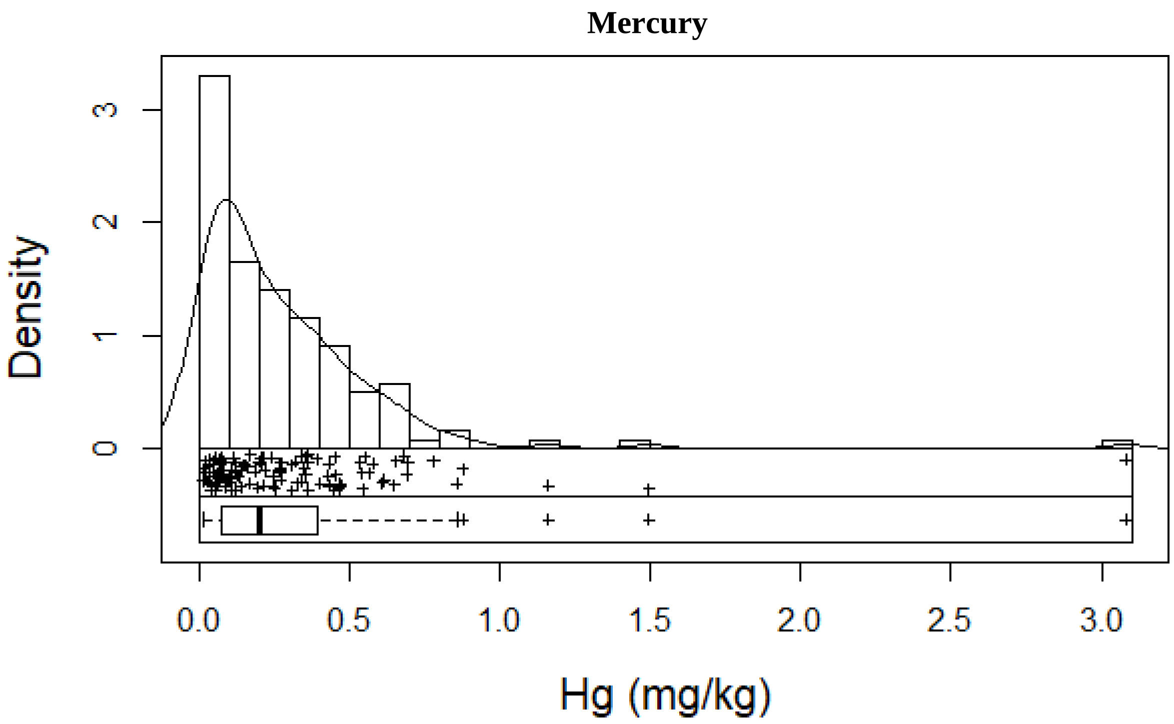
Mercury
Density
3
2
1
0
0.0
0.5
1.0
1.5
2.0
2.5
3.0
Hg (mg/kg)

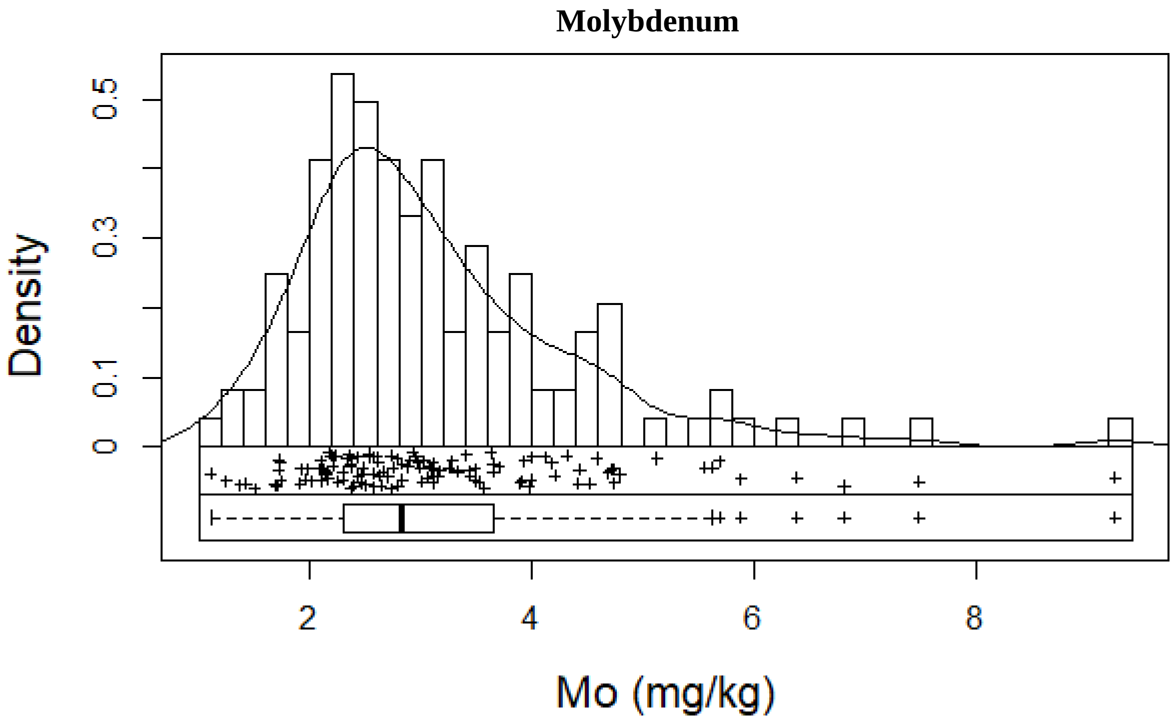
Molybdenum
Density
0.5
0.3
0.1
0
2
4
6
8
Mo (mg/kg)

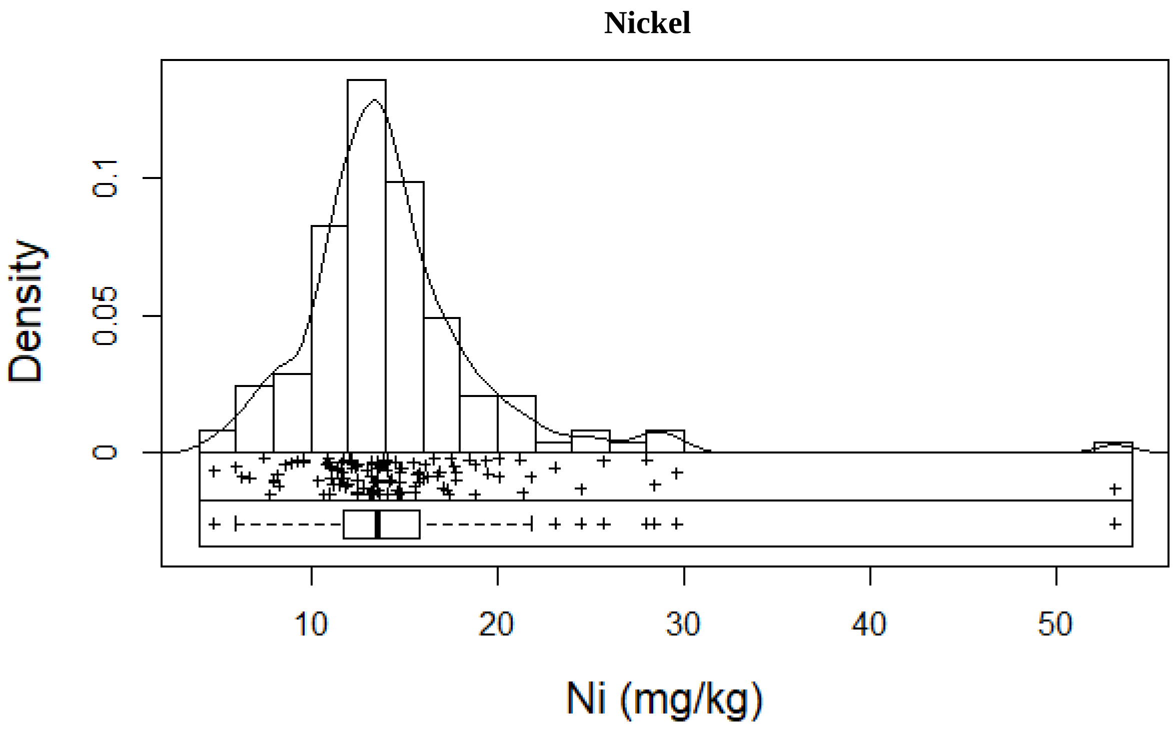

Nickel
Density
0.1
0.05
0
10
20
30
40
50
Ni (mg/kg)


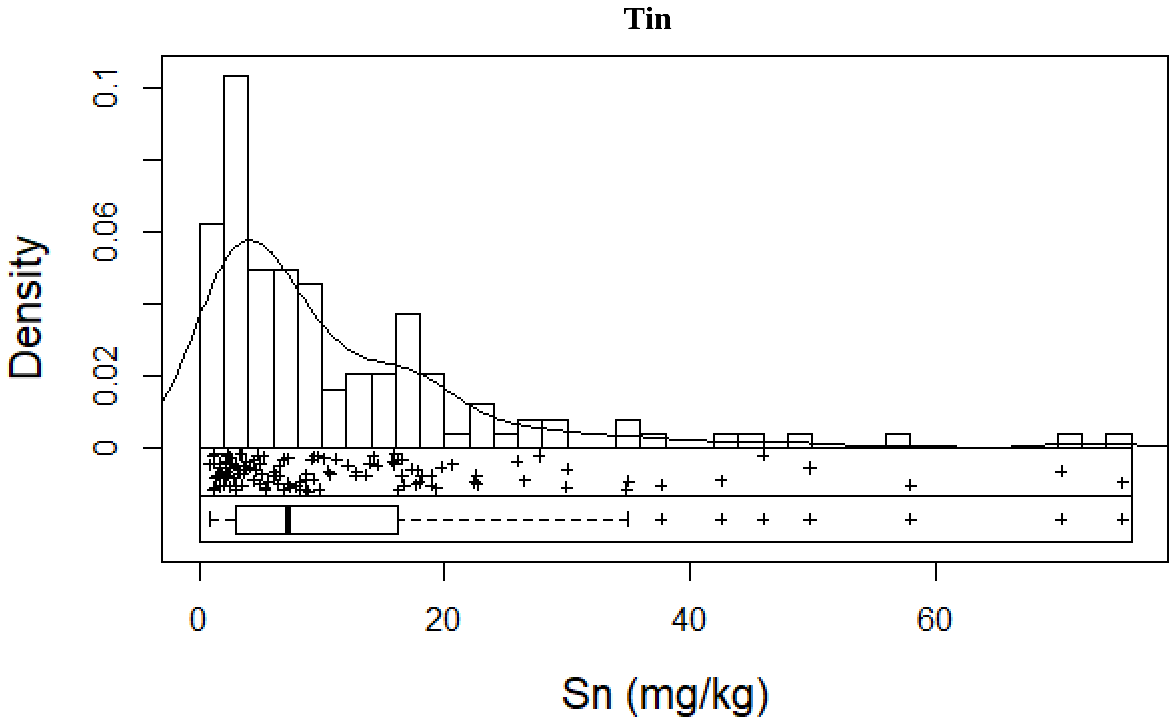

Tin
Density
0.1
0.06
0.02
0
0
20
40
60
Sn (mg/kg)

**Thallium**

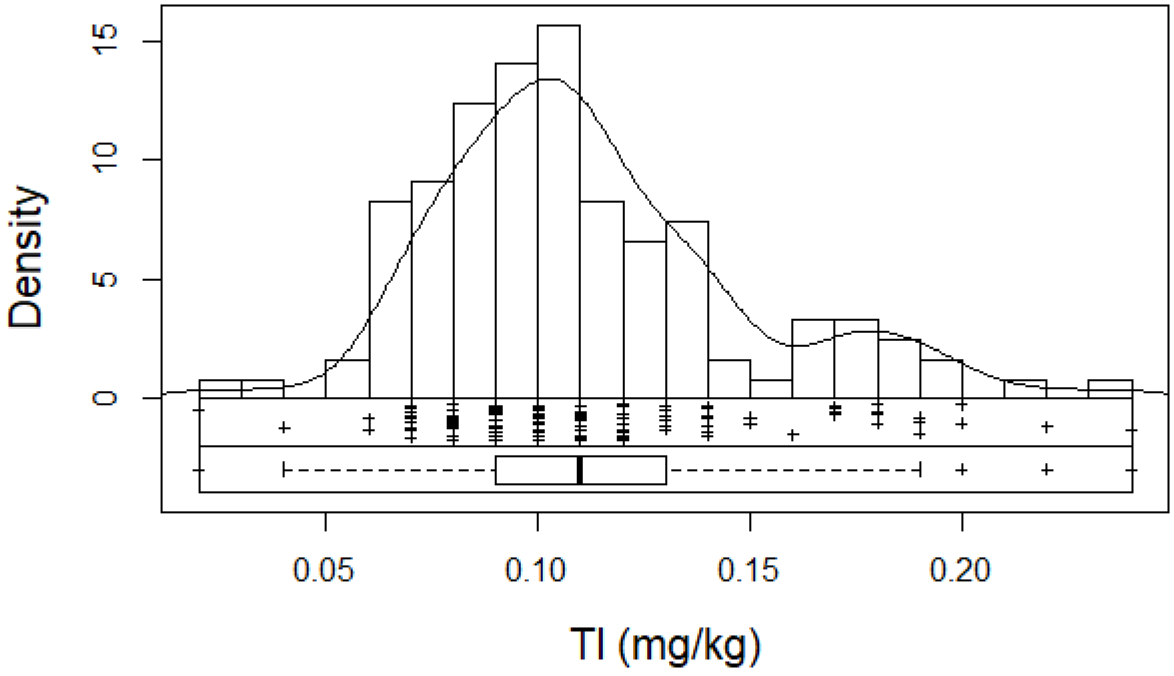


**Vanadium**

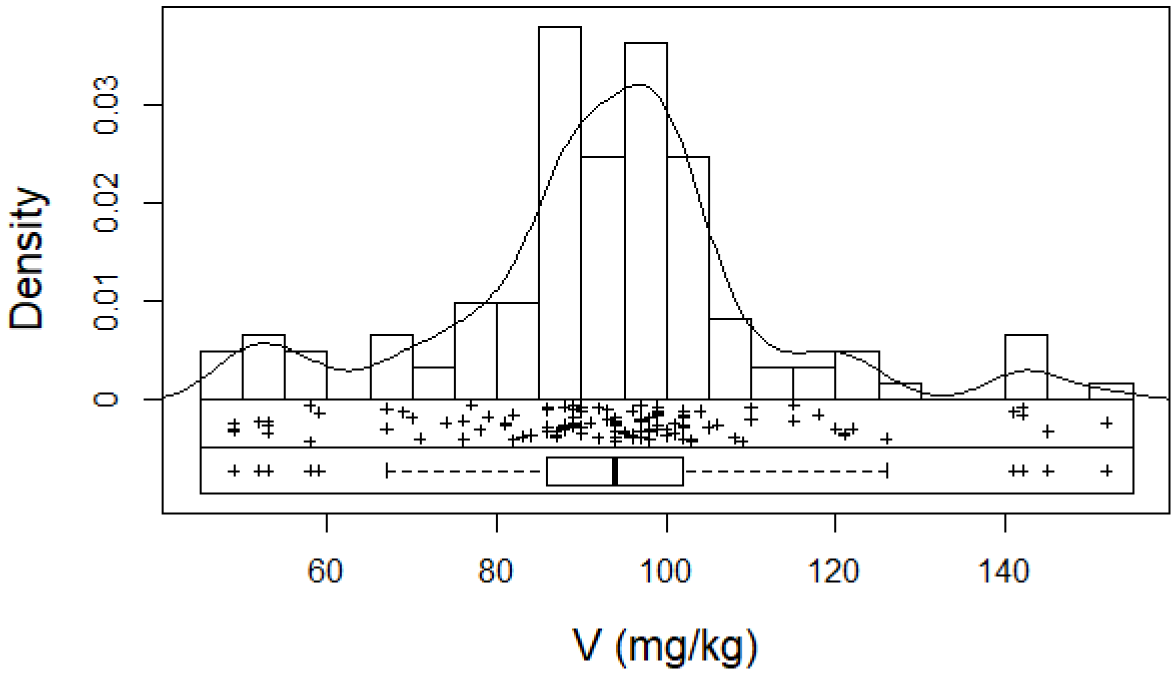


**Zinc**

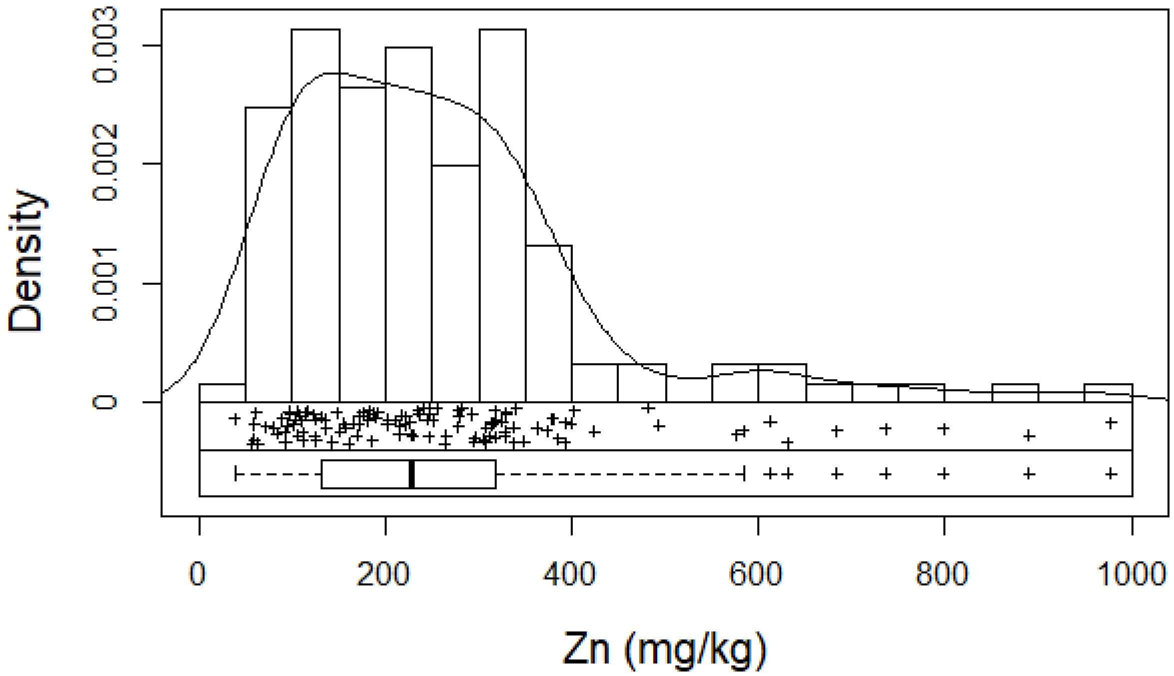

Density
0
0.001
0.002
0.003
0
200
400
600
800
1000
Zn (mg/kg)
