# Supplementary material for: A new approach to assess the degree of contamination and determine sources and risks related to PTEs in an urban environment: the case study of Santiago (Chile)
Source: Environ Geochem Health. 2022 Jan 10;45(2):275–97. doi: 10.1007/s10653-021-01185-6 (PMC9884654; doi:10.1007/s10653-021-01185-6)

A new approach to assess the degree of contamination and determine sources and risks related to PTEs in an urban environment: the case study of Santiago (Chile).

Aruta Antonio[1], Albanese Stefano[1*], Daniele Linda[2], Cannatelli Claudia[3], Buscher Jamie T.[3], De Vivo Benedetto[4,5], Petrik Attila [6], Cicchella Domenico[7], Lima Annamaria[1]

[1] *Department of Earth, Environmental and Resources Sciences, University of Naples Federico II, 80126 Naples, Italy*
[2] *Department of Geology, Andean Geothermal Center of Excellence (CEGA) and Millenium Nucleus for Metal Tracing Along Subduction, FCFM, Universidad de Chile, Plaza Ercilla 803, Santiago, Chile*
[3] *University of Alaska Anchorage, 3211 Providence Drive. Anchorage, AK 99508, USA*
[4] *Virginia Tech, Blacksburg 24061, VA, USA*
[5] *Pegaso On Line University, Piazza Trieste e Trento 48,80132 Naples, Italy*
[6] *Eriksfiord AS, Prof. Olav Hanssensvei 7A, 4021, Stavanger, Norway*
[7] *Department of Science and Technology, University of Sannio, 82100, Benevento, Italy*

**Corresponding author: stefano.albanese@unina.it*

Supplementary Material S3. C-A plots of MIDW maps of selected PTEs

Arsenic (As)

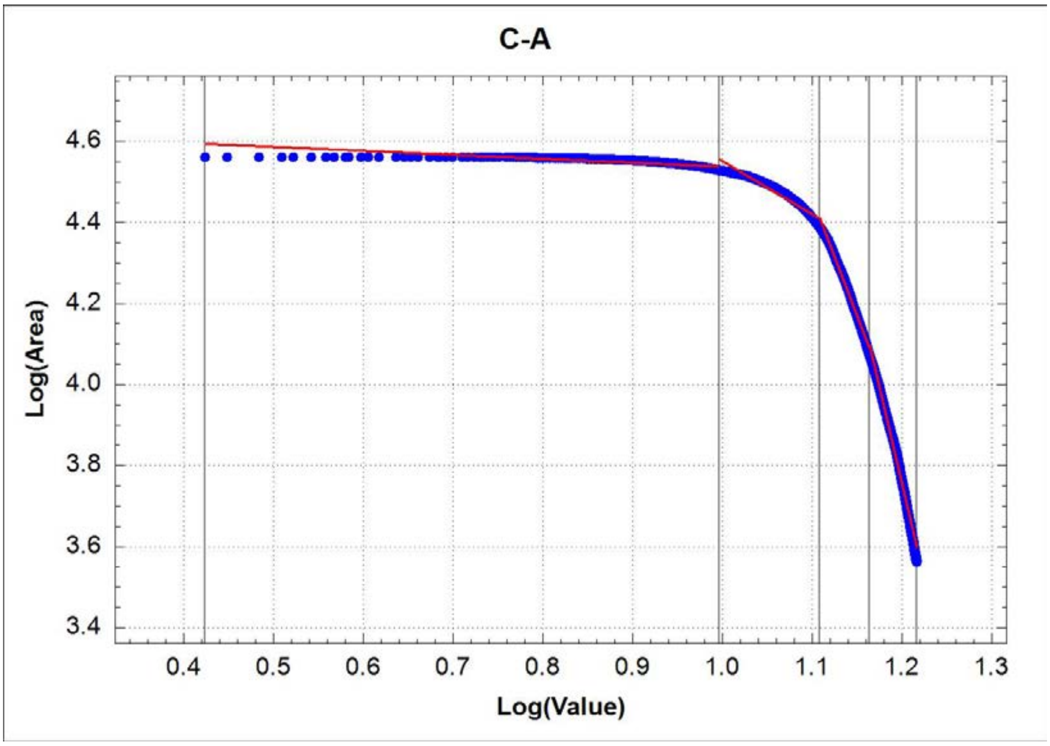

Beryllium (Be)

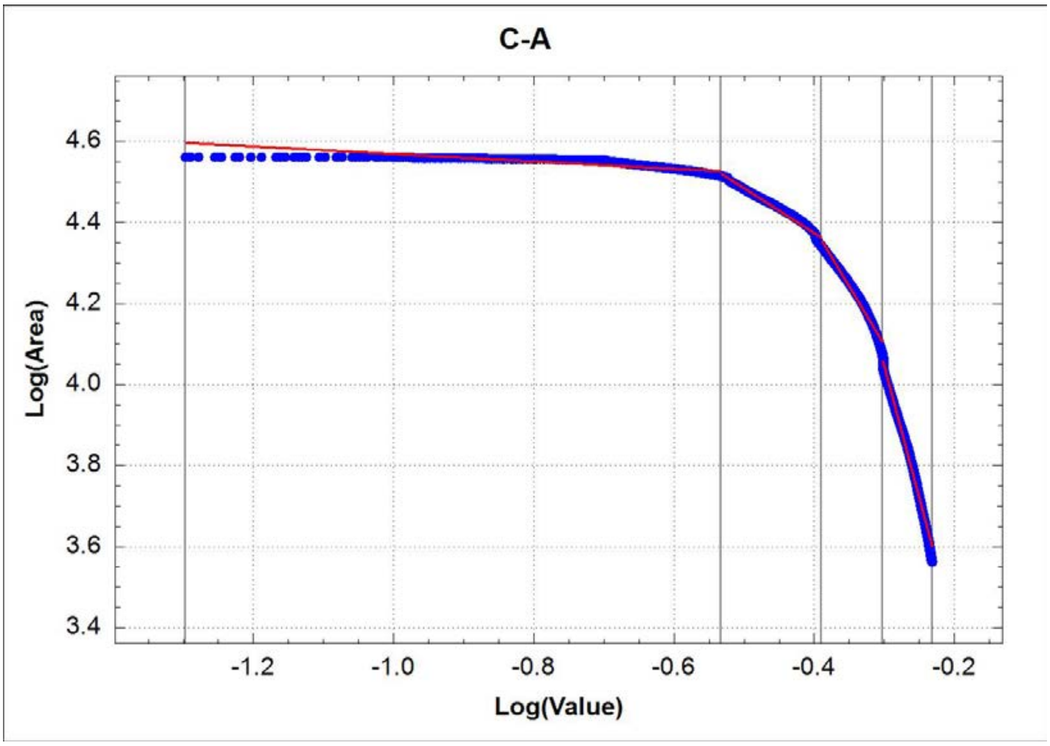

Cadmium (Cd)

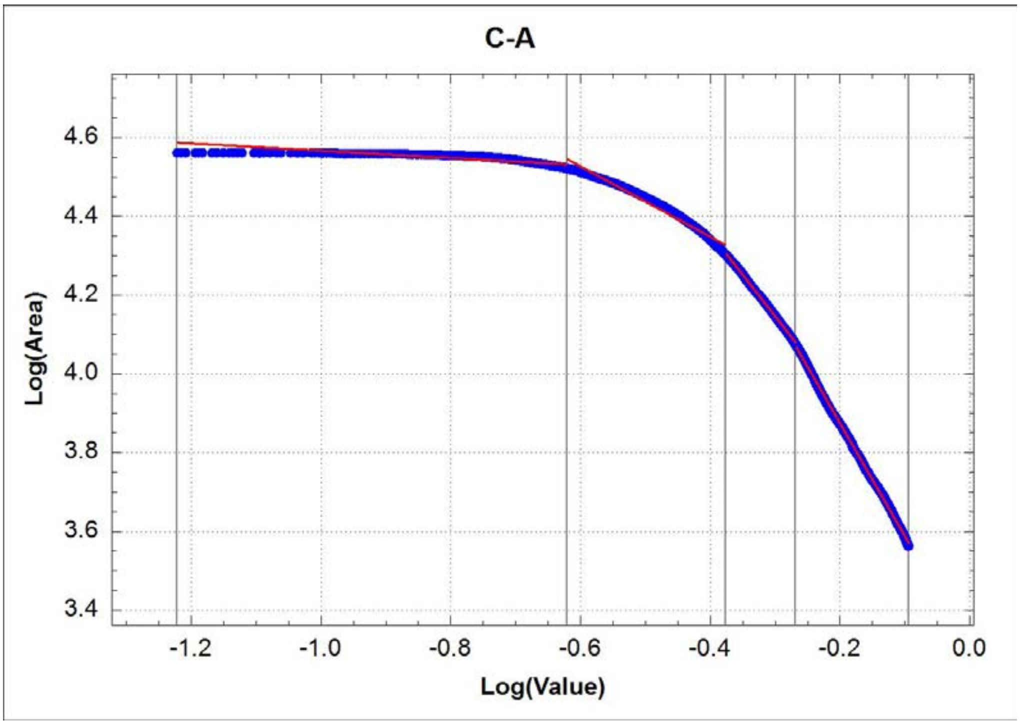

Cobalt (Co)

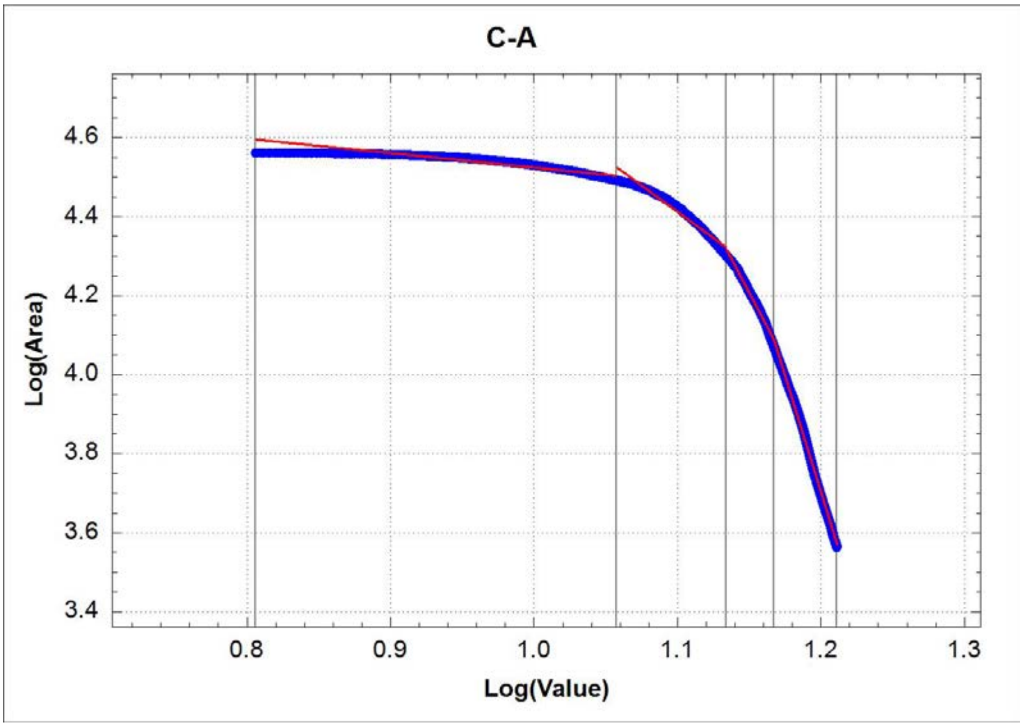

Chromium (Cr)

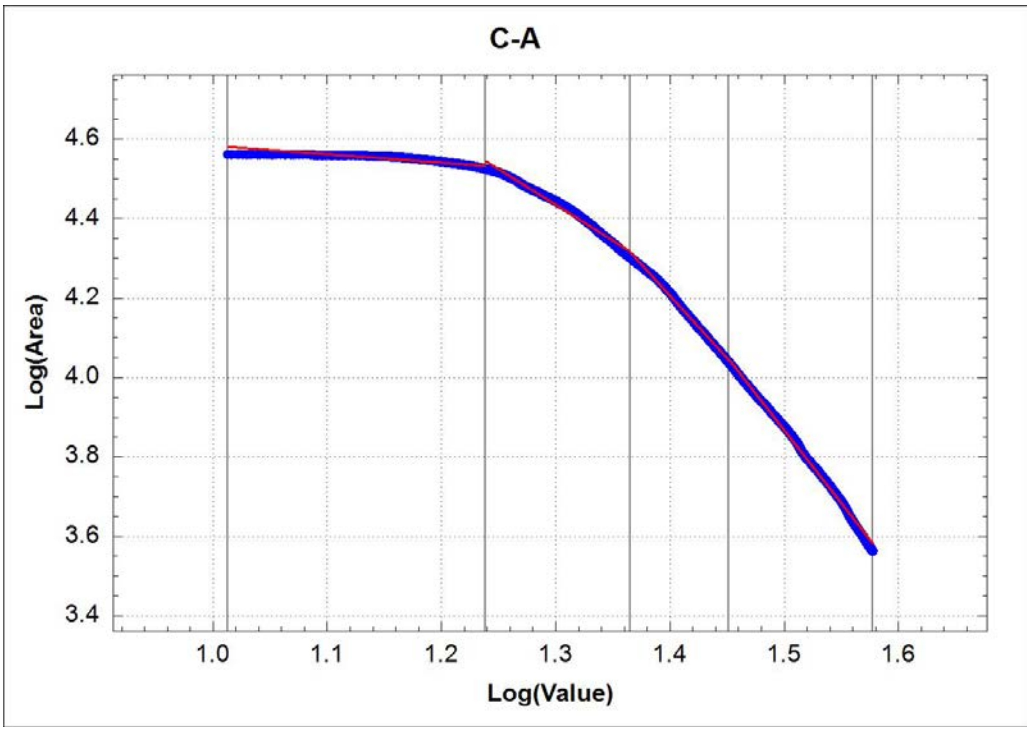

Copper (Cu)

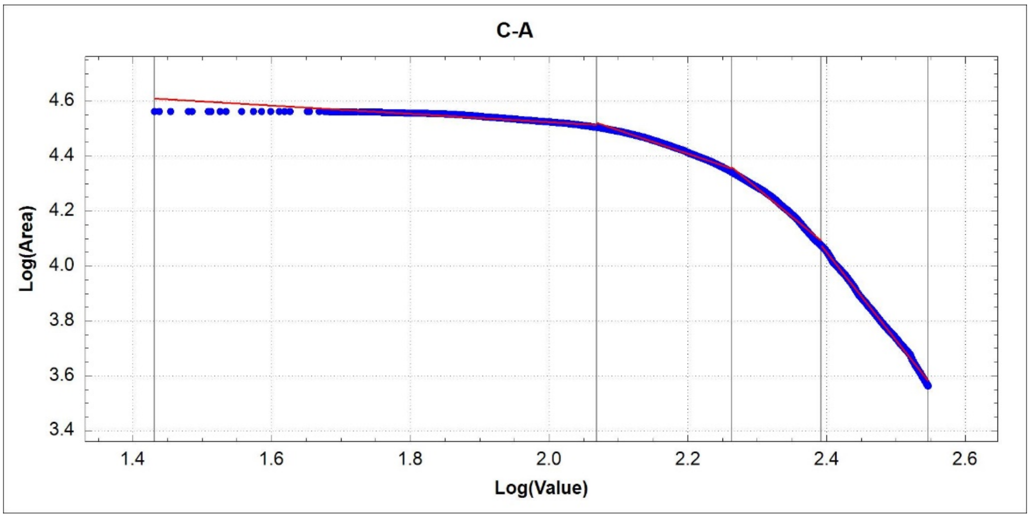

Mercury (Hg)

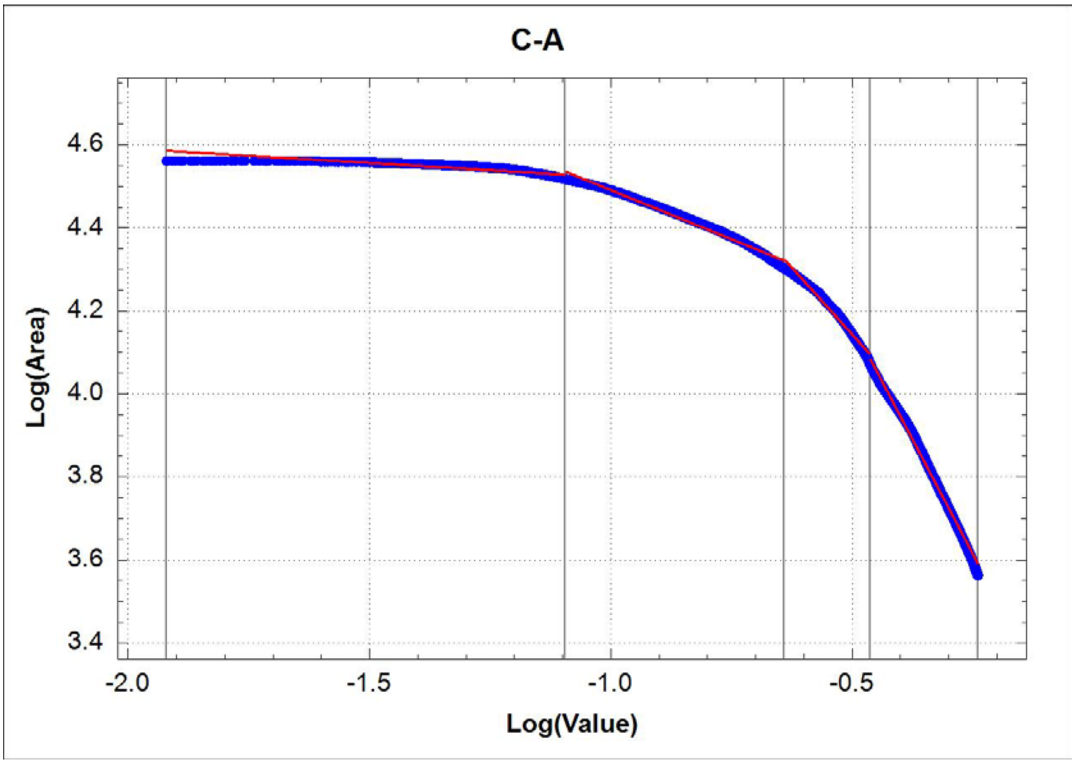

Molybdenum (Mo)

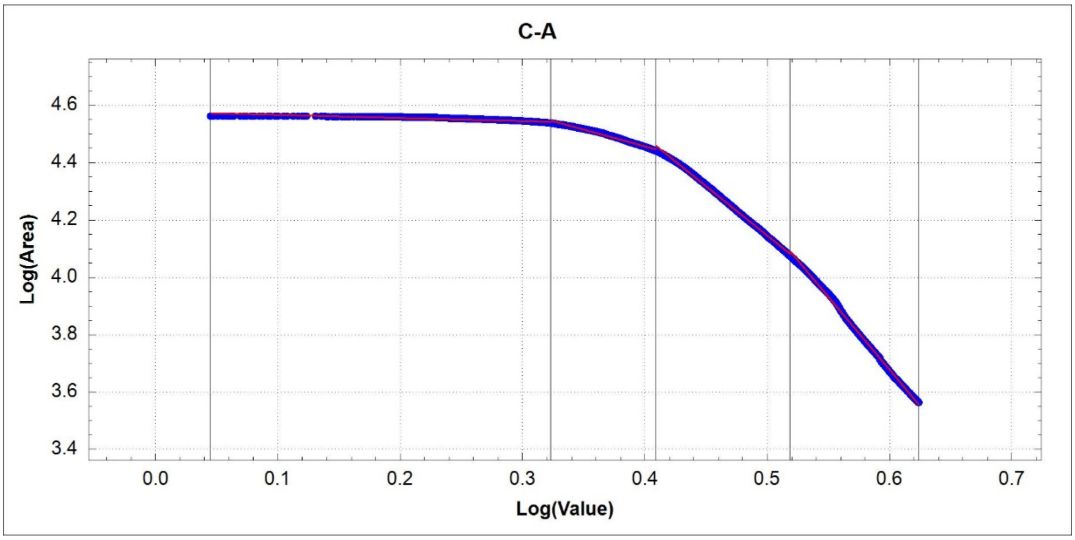

Nickel (Ni)

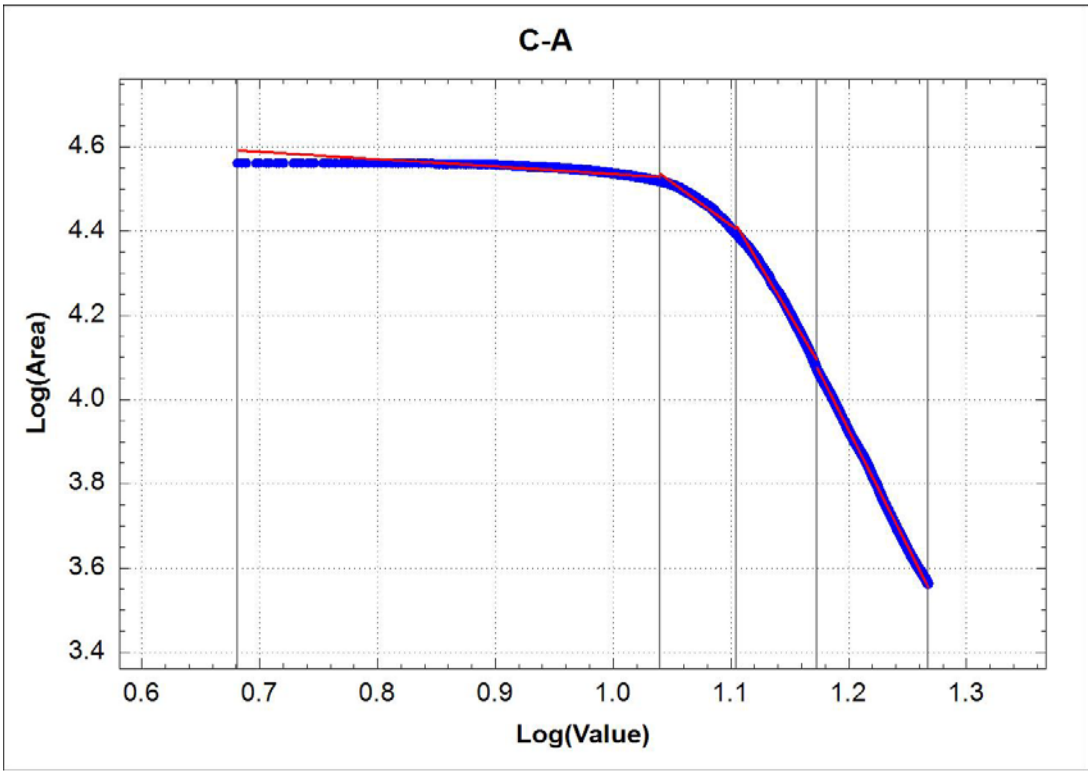

Lead (Pb)

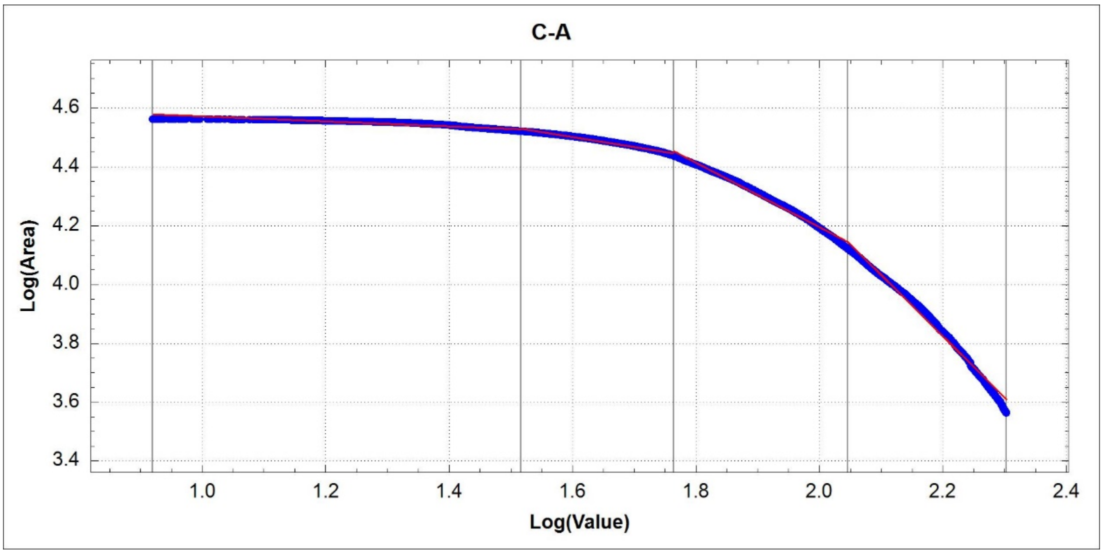

Antimony (Sb)

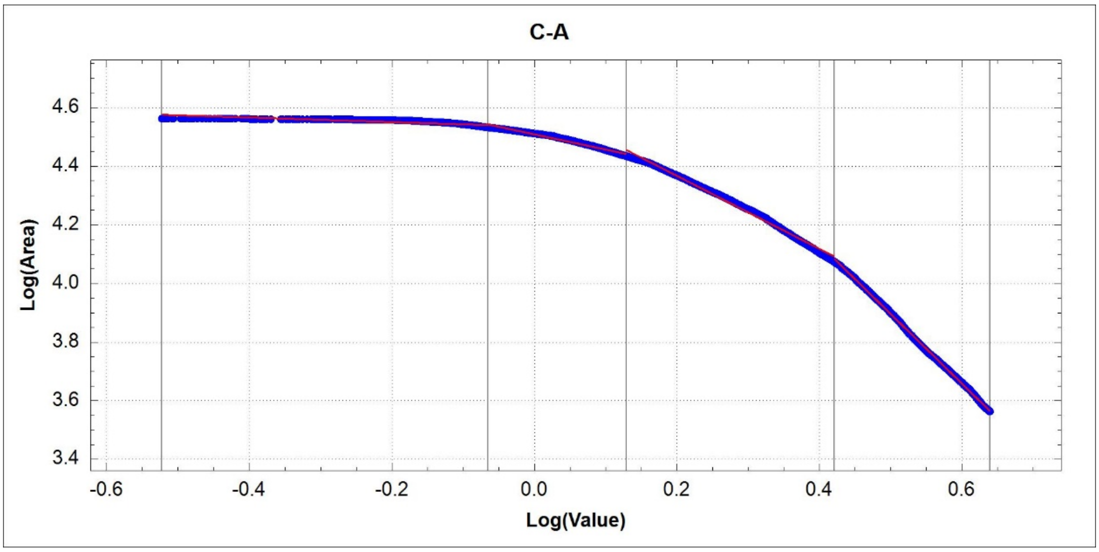

Tin (Sn)

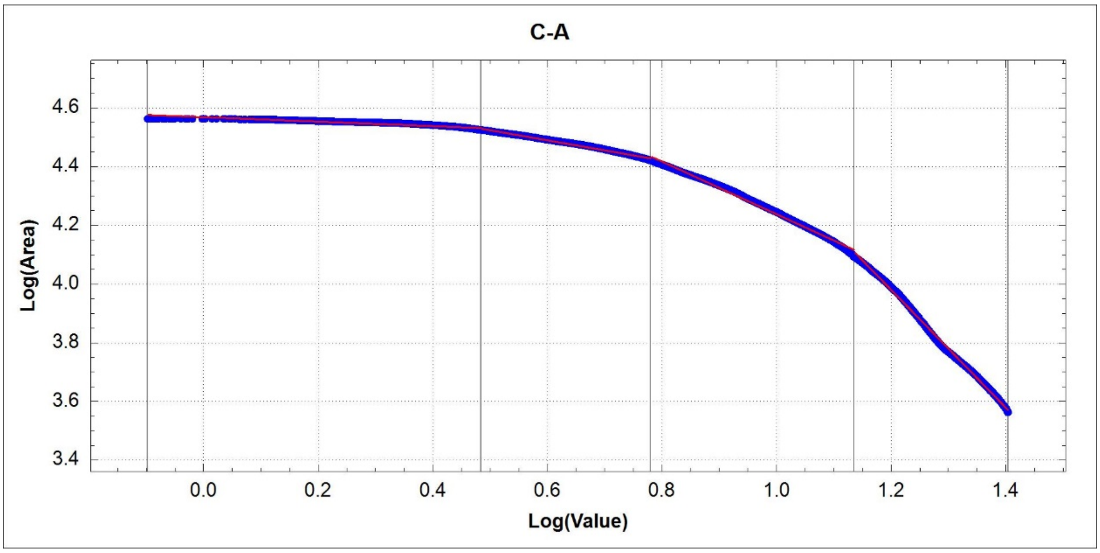

Tallium (Tl)

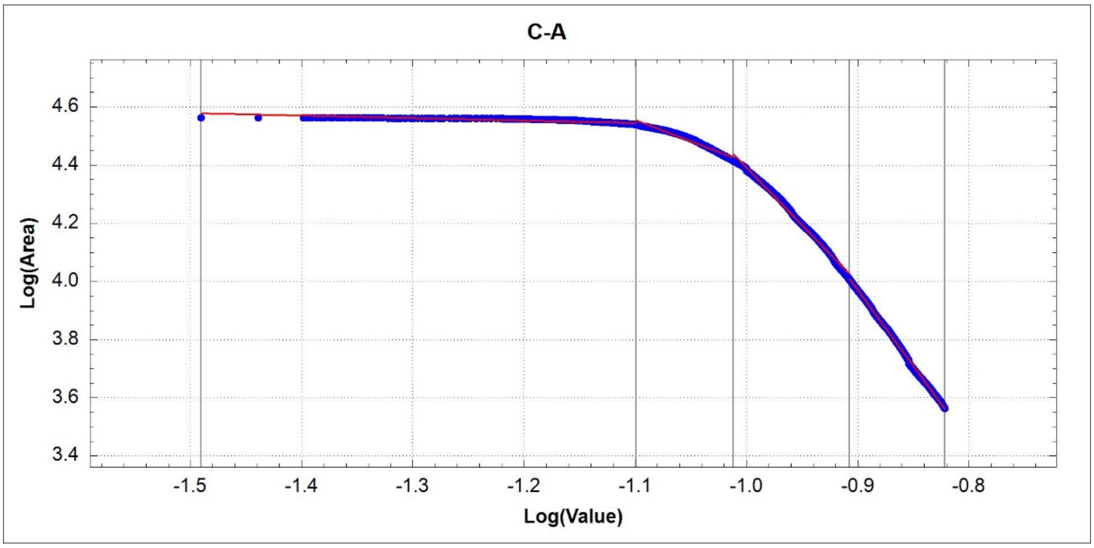

Zinc (Zn)

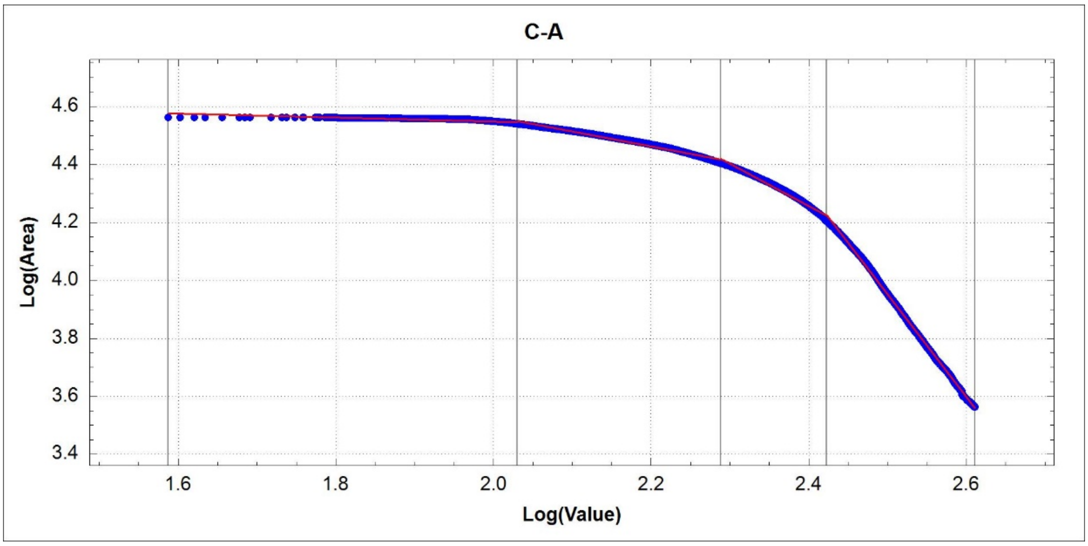

pH

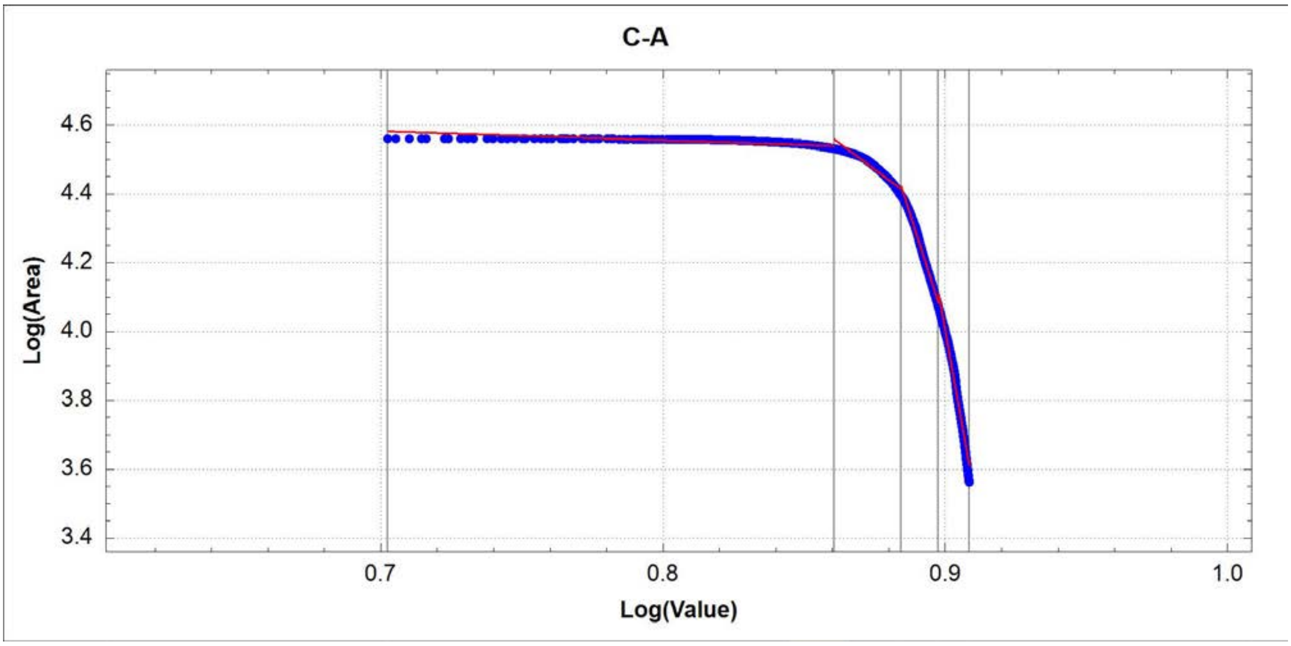

Supplement: Supplementary file 4 — Supplementary file4 (PDF 1444 kb) [file 10653_2021_1185_MOESM4_ESM.pdf]
