# Supplementary material for: A new approach to assess the degree of contamination and determine sources and risks related to PTEs in an urban environment: the case study of Santiago (Chile)
Source: Environ Geochem Health. 2022 Jan 10;45(2):275–97. doi: 10.1007/s10653-021-01185-6 (PMC9884654; doi:10.1007/s10653-021-01185-6)
Supplement: Supplementary file 5 — Supplementary file5 (PDF 132 kb) [file 10653_2021_1185_MOESM5_ESM.pdf]

A new approach to assess the degree of contamination and determine sources and risks related to PTEs in an urban environment: the case study of Santiago (Chile).

Aruta Antonio[1], Albanese Stefano[1*], Daniele Linda[2], Cannatelli Claudia[3], Buscher Jamie T.[3], De Vivo Benedetto[4,5], Petrik Attila [6], Cicchella Domenico[7], Lima Annamaria[1]

[1] *Department of Earth, Environmental and Resources Sciences, University of Naples Federico II, 80126 Naples, Italy*
[2] *Department of Geology, Andean Geothermal Center of Excellence (CEGA) and Millenium Nucleus for Metal Tracing Along Subduction, FCFM, Universidad de Chile, Plaza Ercilla 803, Santiago, Chile*
[3] *University of Alaska Anchorage, 3211 Providence Drive. Anchorage, AK 99508, USA*
[4] *Virginia Tech, Blacksburg 24061, VA, USA*
[5] *Pegaso On Line University, Piazza Trieste e Trento 48,80132 Naples, Italy*
[6] *Eriksfiord AS, Prof. Olav Hanssensvei 7A, 4021, Stavanger, Norway*
[7] *Department of Science and Technology, University of Sannio, 82100, Benevento, Italy*

**Corresponding author: stefano.albanese@unina.it*

Supplementary Material S4. Determination of dosis related to specific exposure pathways

As for the study area, for each PTE, doses, expressed as mg kg^{-1} d^{-1}, were calculated considering, as pathways, ingestion of soil and soil dust inhalation, respectively, and for carcinogenic and/or non-carcinogenic effects (USEPA, 1989, 1997, 2001; USDOE, 2011) as follows (Equations S1, S2, S3, S4):

$$D_{ing-nc} = \frac{C \times IR_{ing} \times EF \times ED \times 10^{-6}}{BW \times AT_{nc}} \qquad \text{(Eq. S1)}$$

$$D_{inh-nc} = \frac{C \times EF \times ET \times ED}{PEF \times 24 \times AT_{nc}} \qquad \text{(Eq. S2)}$$

$$D_{ing-ca} = \frac{C \times IR_{ing} \times EF \times ED \times 10^{-6}}{BW \times AT_{ca}} \qquad \text{(Eq. S3)}$$

$$D_{inh-ca} = \frac{C \times EF \times ET \times ED}{PEF \times 24 \times AT_{ca}} \times 10^{3} \qquad \text{(Eq. S4)}$$

where:

- D_{ing-nc} is the dosis (Expressed as mg kg^{-1} d^{-1}) for soil ingestion for non carcinogenic effects;
- D_{inh-nc} is the dosis (Expressed as mg m^{-3}) for inhalation of soil dust;
- D_{ing-ca} is the dosis (Expressed as mg kg^{-1} d^{-1}) for accidental soil ingestion for carcinogenic effects;
- D_{inh-a} is the dosis (Expressed as μg m^{-3}) for inhalation of soil dust for carcinogenic effects.
- C is the contaminant concentration (Expressed as mg kg^{-1})
- IR_{ing}, EF, ED, BW, AT_{nc}, AT_{ca}, ET, PEF are specific exposure factors whose values and units are reported in the table below:

Variable	Units	Child Ingestion	Adult Inhalation	Child Inhalation	Reference
Soil ingestion rate (**IR_{ing}**)	mg day^{-1}	200	-	-	USEPA 1991
Body weight (**BW**)	kg	19	72	19	USEPA 2011
Expousure Frequency (**EF**)	day $year^{-1}$	350	350	350	USDOE 2011
Expousure Time (**ET**)	h day^{-1}	-	24	24	USDOE 2011
Exposure Duration (**ED**)	years	6	30	6	USEPA 2011
Averaging time non-carcinogenic (**AT_{nc}**)	day (ED*365)	2190	10950	2190	-
Averaging time carcinogenic (**AT_{ca}**)	day (LT*365)	29090.5	29090.5	29090.5	-
Particle emision factor (**PEF**)	m^3 kg^{-1}	-	$1.36*10^9$	$1.36*10^9$	USEPA 2002
Lifetime (**LT**)	years	79.7	79.7	79.7	UNDP, 2012

References

UNDP, (2012). United Nations Development Programme. Human Development Reports available to http://hdr.undp.org/en/69206 - (last accessed 3.2.2020)

USDOE (2011). The risk assessment information system (RAIS). U.S. Oak: Department of Energy's Oak Ridge Operations Office (ORO)

USEPA (1989). Risk Assessment Guidance for Superfund. *Volume I Human Health Evaluation Manual (Part A)*. *I*(December). https://doi.org/EPA/540/1-89/002

USEPA (1997). Guiding Principles for Monte Carlo Analysis. https://doi.org/EPA 630-R-97-001

USEPA (2001). Risk assessment guidance for Superfund: volume III part A, process for conducting probabilistic risk assessment. US Environmental Protection Agency, Washington, DC.

USEPA (2011). Exposure Factors Handbook: 2011 Edition (Issue September National Center for Environmental Assessment, Washington, DC; EPA/600/R-09/052F). https://doi.org/EPA/600/R-090/052F
