# Supplementary material for: A new approach to assess the degree of contamination and determine sources and risks related to PTEs in an urban environment: the case study of Santiago (Chile)
Source: Environ Geochem Health. 2022 Jan 10;45(2):275–97. doi: 10.1007/s10653-021-01185-6 (PMC9884654; doi:10.1007/s10653-021-01185-6)
Supplement: Supplementary file 6 — Supplementary file6 (PDF 50 kb) [file 10653_2021_1185_MOESM6_ESM.pdf]

A new approach to assess the degree of contamination and determine sources and risks related to PTEs in an urban environment: the case study of Santiago (Chile).

Aruta Antonio[1], Albanese Stefano[1*], Daniele Linda[2], Cannatelli Claudia[3], Buscher Jamie T.[3], De Vivo Benedetto[4,5], Petrik Attila [6], Cicchella Domenico[7], Lima Annamaria[1]

[1] *Department of Earth, Environmental and Resources Sciences, University of Naples Federico II, 80126 Naples, Italy*
[2] *Department of Geology, Andean Geothermal Center of Excellence (CEGA) and Millenium Nucleus for Metal Tracing Along Subduction, FCFM, Universidad de Chile, Plaza Ercilla 803, Santiago, Chile*
[3] *University of Alaska Anchorage, 3211 Providence Drive. Anchorage, AK 99508, USA*
[4] *Virginia Tech, Blacksburg 24061, VA, USA*
[5] *Pegaso On Line University, Piazza Trieste e Trento 48,80132 Naples, Italy*
[6] *Eriksfiord AS, Prof. Olav Hanssensvei 7A, 4021, Stavanger, Norway*
[7] *Department of Science and Technology, University of Sannio, 82100, Benevento, Italy*

**Corresponding author: stefano.albanese@unina.it*

Supplementary Material S5. Toxicological values for carcinogenic and non-carcinogenic effects of selected PTEs (where available) for both ingestion and inhalation pathways.

Element	Carcinogenic		Non-carcinogenic	
	Ingestion	Inhalation	Ingestion	Inhalation
	**SF** mg/kg-day	**IUR** (µg/m ^{3}) $^{-1}$	**RfD** mg/kg-day	**RfC** mg/m3
As	1.50E+00	4.30E-03	3.00E-04	1.50E-05
Be	-	2.40E-03	2.00E-03	2.00E-05
Cd	-	1.80E-03	1.00E-03	1.00E-05
Co	-	9.00E-03	3.00E-04	6.00E-06
Cr_{Tot}	-	1.20E-02[a]	3.00E-03[a]	-
Cu	-	-	4.00E-02	-
Hg	-	-	1.60E-04	3.00E-04
Mo	-	-	5.00E-03	4.00E-04
Ni	-	2.60E-04	2.00E-02	9.00E-05
Pb	8.50E-03[b]	1.20E-05	3.50E-03	-
Sb	-	-	4.00E-04	3.00E-04
Sn	-	-	6.00E-01	
Tl	-	-	-	-
V	-	-	5.04E-03	
Zn	-	-	3.00E-01	-

All reported values were extracted from the the Risk Assessment Information System (RAIS) database (available at https://rais.ornl.gov/ - Last accessed on 03.02.2020), except a) USEPA, 2002 and b) WHO, 1993.

References

The Risk Assessment Information System (RAIS). Available at https://rais.ornl.gov/ - Last accessed on 03.02.2020

USEPA, (2002). Supplemental guidance for developing soil screening levels for superfund sites U.S. Environmental Protection Agency (2002), pp. 4-24
OSWER 9355

WHO, (1993). Guidelines for drinking-water quality, Volume 1 — recommendation World Health Organization (1993)
