# Supplementary material for: A new approach to assess the degree of contamination and determine sources and risks related to PTEs in an urban environment: the case study of Santiago (Chile)
Source: Environ Geochem Health. 2022 Jan 10;45(2):275–97. doi: 10.1007/s10653-021-01185-6 (PMC9884654; doi:10.1007/s10653-021-01185-6)
Supplement: Supplementary file 7 — Supplementary file7 (PDF 74 kb) [file 10653_2021_1185_MOESM7_ESM.pdf]

A new approach to assess the degree of contamination and determine sources and risks related to PTEs in an urban environment: the case study of Santiago (Chile).

Aruta Antonio[1], Albanese Stefano[1*], Daniele Linda[2], Cannatelli Claudia[3], Buscher Jamie T.[3], De Vivo Benedetto[4,5], Petrik Attila [6], Cicchella Domenico[7], Lima Annamaria[1]

[1] *Department of Earth, Environmental and Resources Sciences, University of Naples Federico II, 80126 Naples, Italy*
[2] *Department of Geology, Andean Geothermal Center of Excellence (CEGA) and Millenium Nucleus for Metal Tracing Along Subduction, FCFM, Universidad de Chile, Plaza Ercilla 803, Santiago, Chile*
[3] *University of Alaska Anchorage, 3211 Providence Drive. Anchorage, AK 99508, USA*
[4] *Virginia Tech, Blacksburg 24061, VA, USA*
[5] *Pegaso On Line University, Piazza Trieste e Trento 48,80132 Naples, Italy*
[6] *Eriksfiord AS, Prof. Olav Hanssensvei 7A, 4021, Stavanger, Norway*
[7] *Department of Science and Technology, University of Sannio, 82100, Benevento, Italy*

**Corresponding author: stefano.albanese@unina.it*

Supplementary Material S6. A table reposrting a list of target organs and systems in human body associated to PTEs which could generate cumulative non-carcinogenic adverse effect on them in case of an environmental exposure through ingestion or inhalation. All information was retrieved from the Risk Assessment Information System (RAIS) database (Available at https://rais.ornl.gov/ - Last accessed on 03.02.2020).

	**Target Organ/System**	**As**	**Be**	**Cd**	**Co**	**Cr_{tot}**	**Cu**	**Hg**	**Mo**	**Ni**	**Pb**	**Sb**	**Sn**	**V**	**Zn**
**Ingestion**	Blood									X				X	X
	Cardiovascular system	X									X				
	Central nervous system							X			X				
	Development						X								
	Gastrointestinal system						X							X	
	Gastrointestinal tract			X								X			X
	Hematopeic system						X								
	Kidneys			X			X	X		X	X			X	
	Liver						X		X						
	Nervous system	X													
	Red blood cells										X				
	Pancreas														X
	Reproduction						X								
	Skeletal system		X						X						
	Skin	X													
	NA					X									
**Inhalation**	Cardiovascular System	X									X				
	Central nervous system							X			X				
	Gastrointestinal Tract						X								
	Immune system									X					
	Kidney			X				X			X				
	Liver						X								
	Lung		X	X					X						X
	Nervous System	X					X								
	Red blood cell										X				
	Respiratory system					X								X	
	Respiratory tract						X			X		X			
	Skeletal system								X						
	Skin	X													
