# Supplementary material for: A new approach to assess the degree of contamination and determine sources and risks related to PTEs in an urban environment: the case study of Santiago (Chile)
Source: Environ Geochem Health. 2022 Jan 10;45(2):275–97. doi: 10.1007/s10653-021-01185-6 (PMC9884654; doi:10.1007/s10653-021-01185-6)
Supplement: Supplementary file 9 — Supplementary file9 (PDF 4726 kb) [file 10653_2021_1185_MOESM9_ESM.pdf]

A new approach to assess the degree of contamination and determine sources and risks related to PTEs in an urban environment: the case study of Santiago (Chile).

Aruta Antonio[1], Albanese Stefano[1*], Daniele Linda[2], Cannatelli Claudia[3], Buscher Jamie T.[3], De Vivo Benedetto[4,5], Petrik Attila [6], Cicchella Domenico[7], Lima Annamaria[1]

[1] *Department of Earth, Environmental and Resources Sciences, University of Naples Federico II, 80126 Naples, Italy*
[2] *Department of Geology, Andean Geothermal Center of Excellence (CEGA) and Millenium Nucleus for Metal Tracing Along Subduction, FCFM, Universidad de Chile, Plaza Ercilla 803, Santiago, Chile*
[3] *University of Alaska Anchorage, 3211 Providence Drive. Anchorage, AK 99508, USA*
[4] *Virginia Tech, Blacksburg 24061, VA, USA*
[5] *Pegaso On Line University, Piazza Trieste e Trento 48,80132 Naples, Italy*
[6] *Eriksfiord AS, Prof. Olav Hanssensvei 7A, 4021, Stavanger, Norway*
[7] *Department of Science and Technology, University of Sannio, 82100, Benevento, Italy*

**Corresponding author: stefano.albanese@unina.it*

Supplementary Material S8. The folder contains the results of Monte Carlo simulation applied to risk assessment for ingestion (only for children) and inhalation (for both children and adults) of soil/dust for the commune of Santiago.

For non-carcinogenic risk:

- the distribution of HQs is reported for each element, pathway, and receptor as frequency histograms.
- the distribution of HIs is reported for each target organs/system.

For carcinogenic risk:

- the distribution of ILCRs is reported for each element, pathway, and receptor as frequency histograms.

Summaries of univariate statistics for HQs and ILCRs distribution are reported as tables.

HQs INGESTION CHILDREN

HQ ing Child

Element	**As**	**Be**	**Cd**	**Co**	**Cr_{Tot}**	**Cu**	**Hg**	**Mo**	**Ni**	**Pb**	**Sb**	**Sn**	**V**	**Zn**
Level of certainty	**99.994%**	0.000%	0.000%	**98.864%**	**2.443%**	**1.301%**	**0.001%**	0.000%	0.000%	**51.184%**	**3.881%**	0.000%	**100%**	0.000%
Mean	7.62E-01	2.20E-03	4.80E-03	4.65E-01	9.74E-02	5.61E-02	1.82E-02	6.30E-03	7.96E-03	3.06E-01	6.23E-02	2.00E-04	9.31E-01	8.90E-03
Median	7.70E-01	2.20E-03	3.90E-03	4.65E-01	8.80E-02	4.41E-02	1.27E-02	5.70E-03	7.55E-03	2.05E-01	4.12E-02	1.30E-04	9.11E-01	7.50E-03
Min	1.52E-01	-1.60E-03	8.00E-04	-2.21E-01	3.65E-02	8.49E-03	2.79E-08	2.50E-03	3.33E-02	2.73E-02	8.03E-03	2.00E-05	3.71E-01	1.30E-03
Max	1.24E+00	5.70E-03	5.01E-02	1.31E+00	6.12E-01	1.70E+00	2.04E-01	3.93E-02	1.00E-05	1.11E+01	3.12E+00	5.28E-03	2.05E+00	5.59E-02
St. Dev.	1.51E-01	9.00E-04	3.20E-03	1.07E-01	3.96E-02	4.24E-02	1.82E-02	2.60E-03	2.48E-03	3.41E-01	7.08E-02	2.20E-04	1.96E-01	5.50E-03

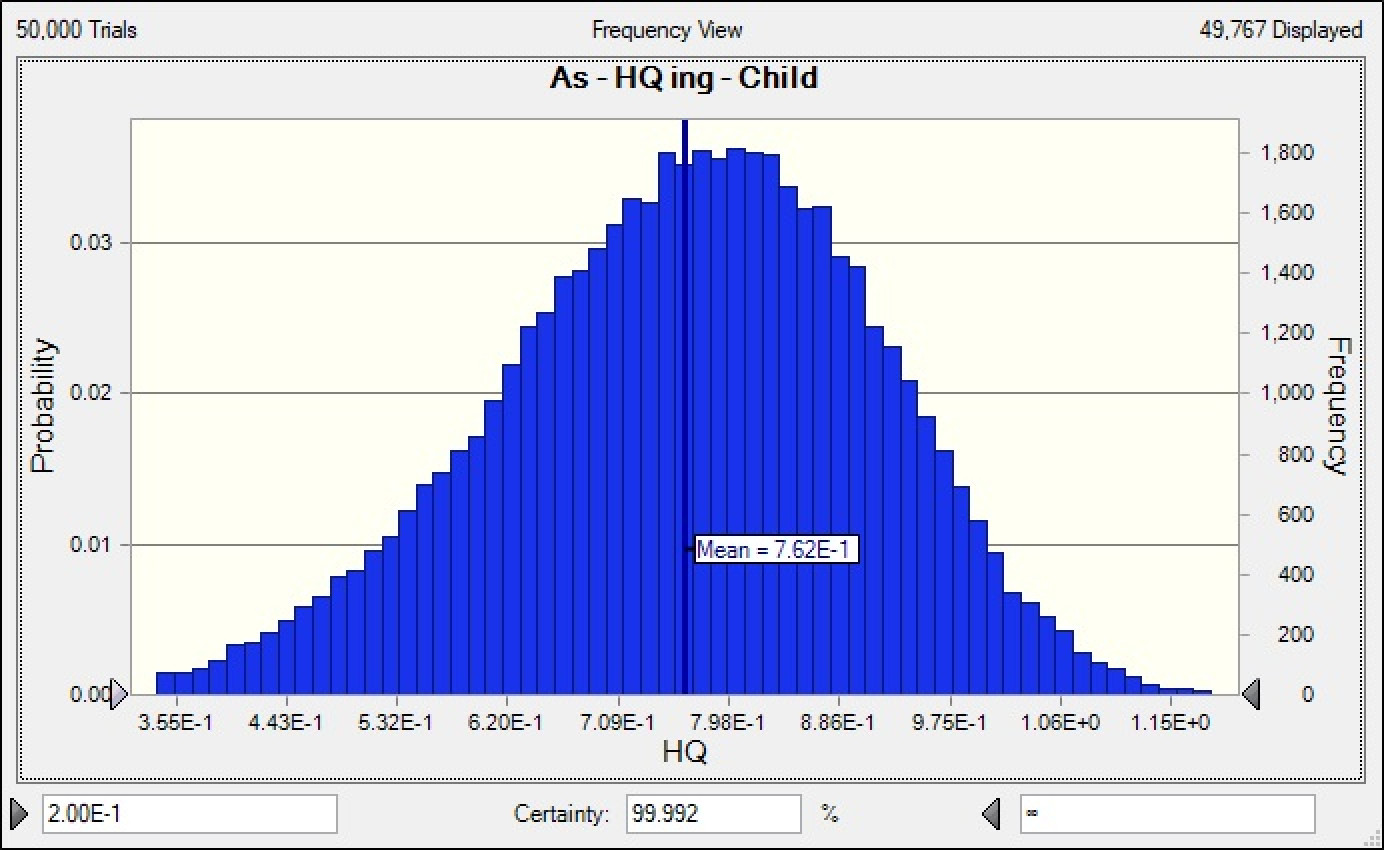

50,000 Trials
Frequency View
49,767 Displayed
As - HQ ing - Child
Probability
Frequency
0.03
0.02
0.01
0.00
1,800
1,600
1,400
1,200
1,000
800
600
400
200
0
Mean = 7.62E-1
3.55E-1
4.43E-1
5.32E-1
6.20E-1
7.09E-1
7.98E-1
8.86E-1
9.75E-1
1.06E+0
1.15E+0
HQ
2.00E-1
Certainty:
99.992
%
∞

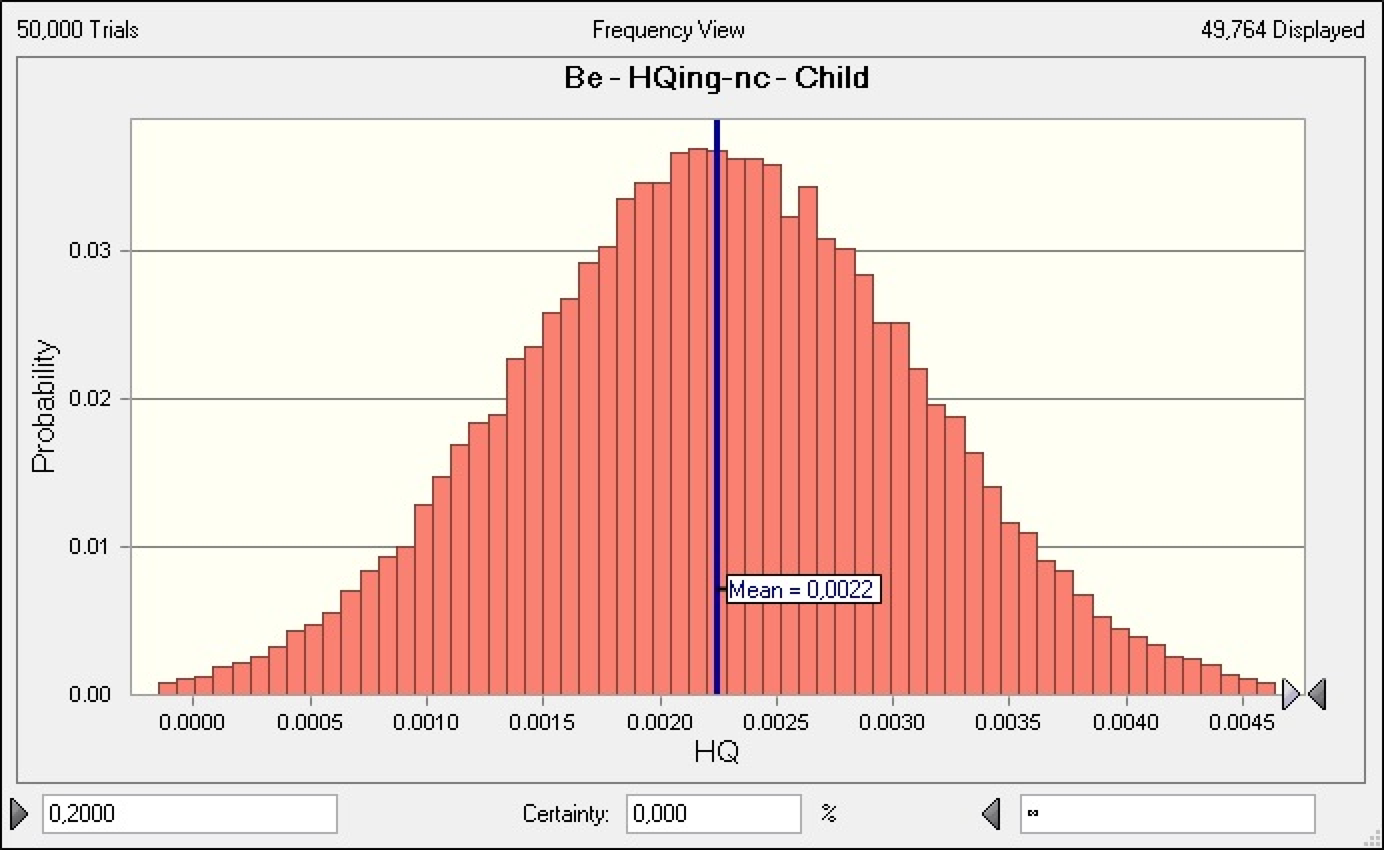

50,000 Trials
Frequency View
49,764 Displayed
Be - HQing-nc - Child
Probability
0.03
0.02
0.01
0.00
0.0000
0.0005
0.0010
0.0015
0.0020
0.0025
0.0030
0.0035
0.0040
0.0045
HQ
Mean = 0,0022
0,2000
Certainty:
0,000
%
∞

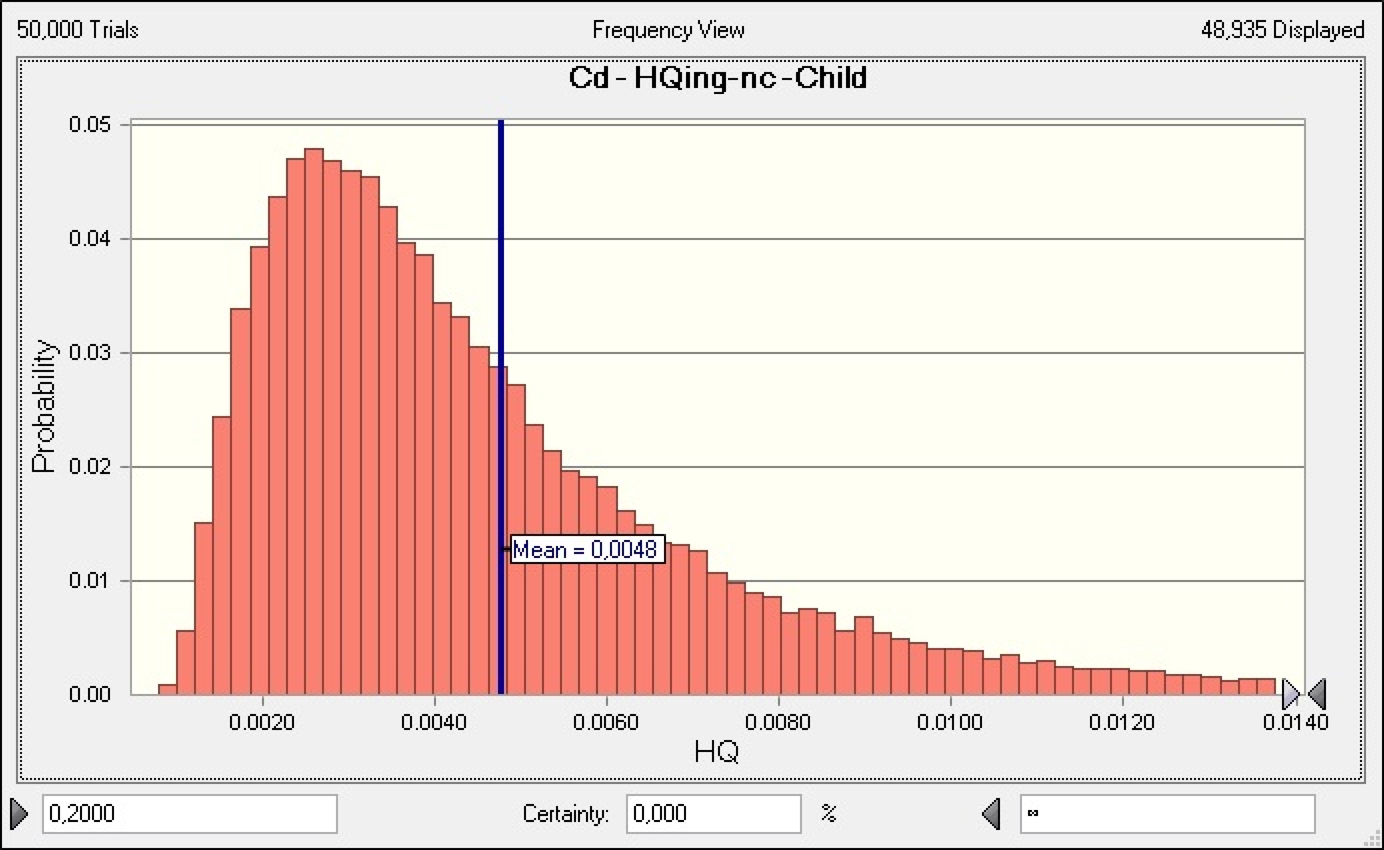
50,000 Trials
Frequency View
48,935 Displayed
Cd - HQing-nc-Child
Probability
0.05
0.04
0.03
0.02
0.01
0.00
Mean = 0,0048
0.0020
0.0040
0.0060
0.0080
0.0100
0.0120
0.0140
HQ
0,2000
Certainty:
0,000
%
∞

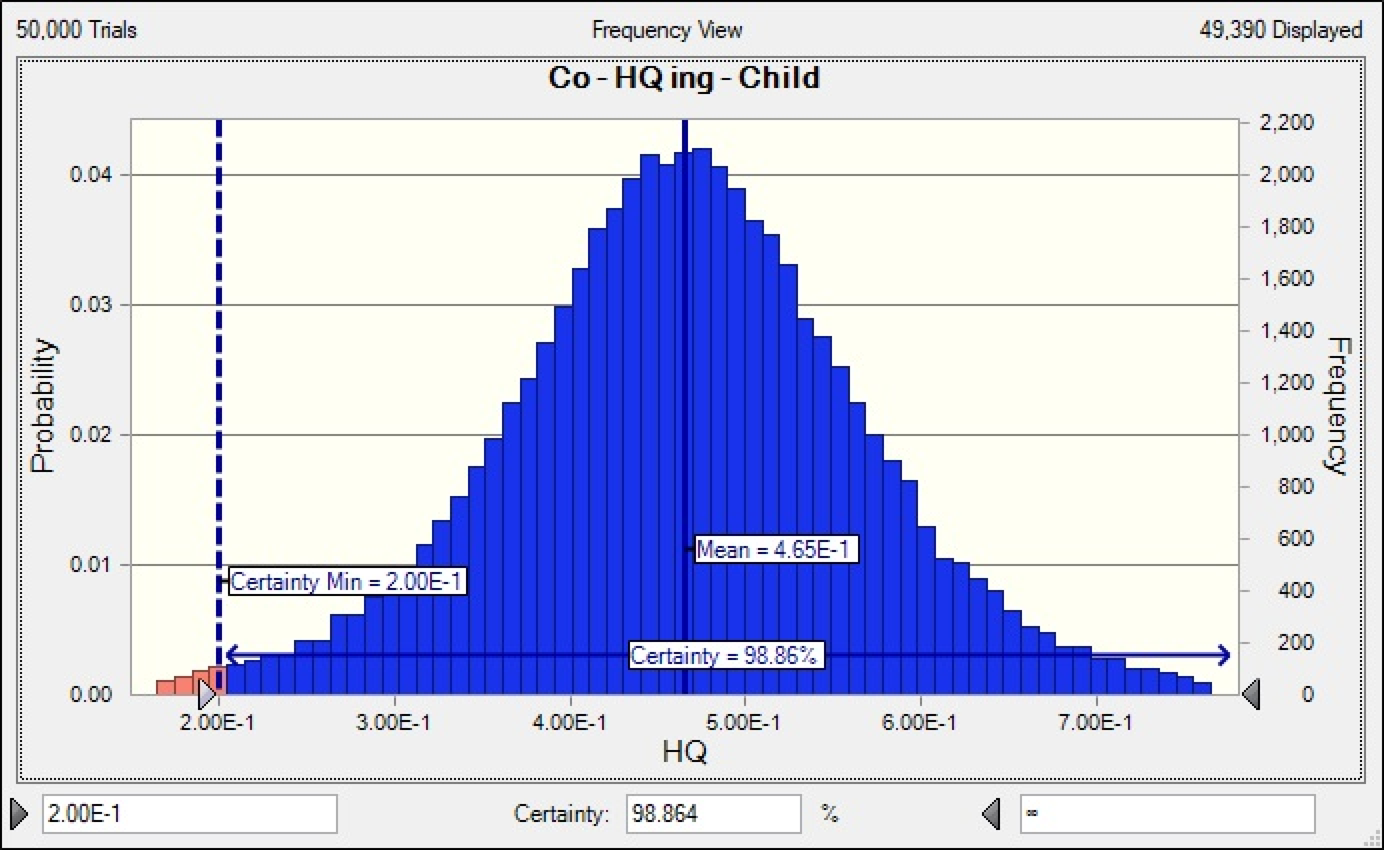

50,000 Trials
Frequency View
49,390 Displayed
Co - HQ ing - Child
Probability
Frequency
0.04
0.03
0.02
0.01
0.00
2,200
2,000
1,800
1,600
1,400
1,200
1,000
800
600
400
200
0
Mean = 4.65E-1
Certainty Min = 2.00E-1
Certainty = 98.86%
2.00E-1
3.00E-1
4.00E-1
5.00E-1
6.00E-1
7.00E-1
HQ
2.00E-1
Certainty:
98.864
%
∞

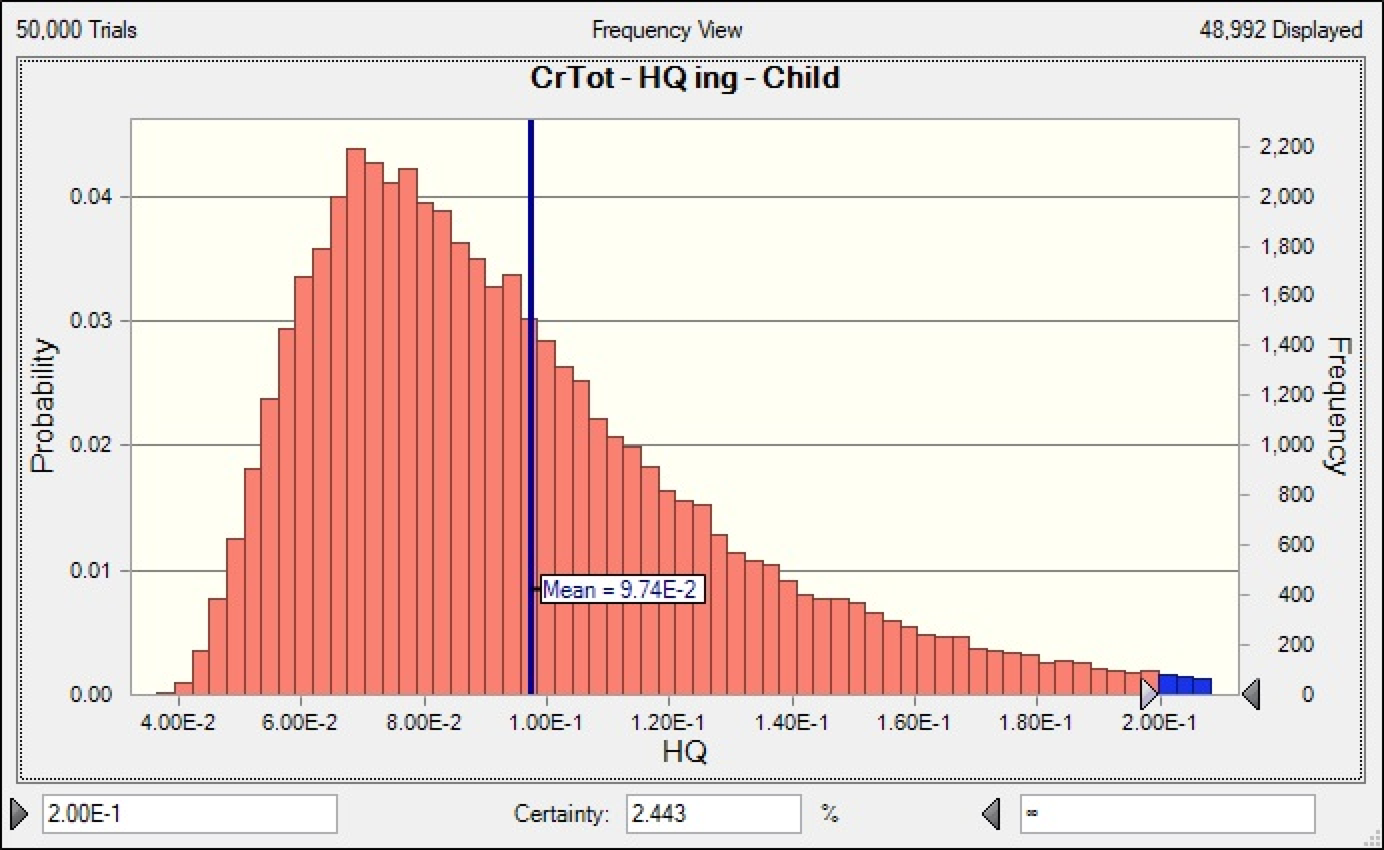
50,000 Trials
Frequency View
48,992 Displayed
CrTot - HQ ing - Child
Probability
Frequency
0.00
0.01
0.02
0.03
0.04
0
200
400
600
800
1,000
1,200
1,400
1,600
1,800
2,000
2,200
Mean = 9.74E-2
4.00E-2
6.00E-2
8.00E-2
1.00E-1
1.20E-1
1.40E-1
1.60E-1
1.80E-1
2.00E-1
HQ
2.00E-1
Certainty:
2.443
%
∞

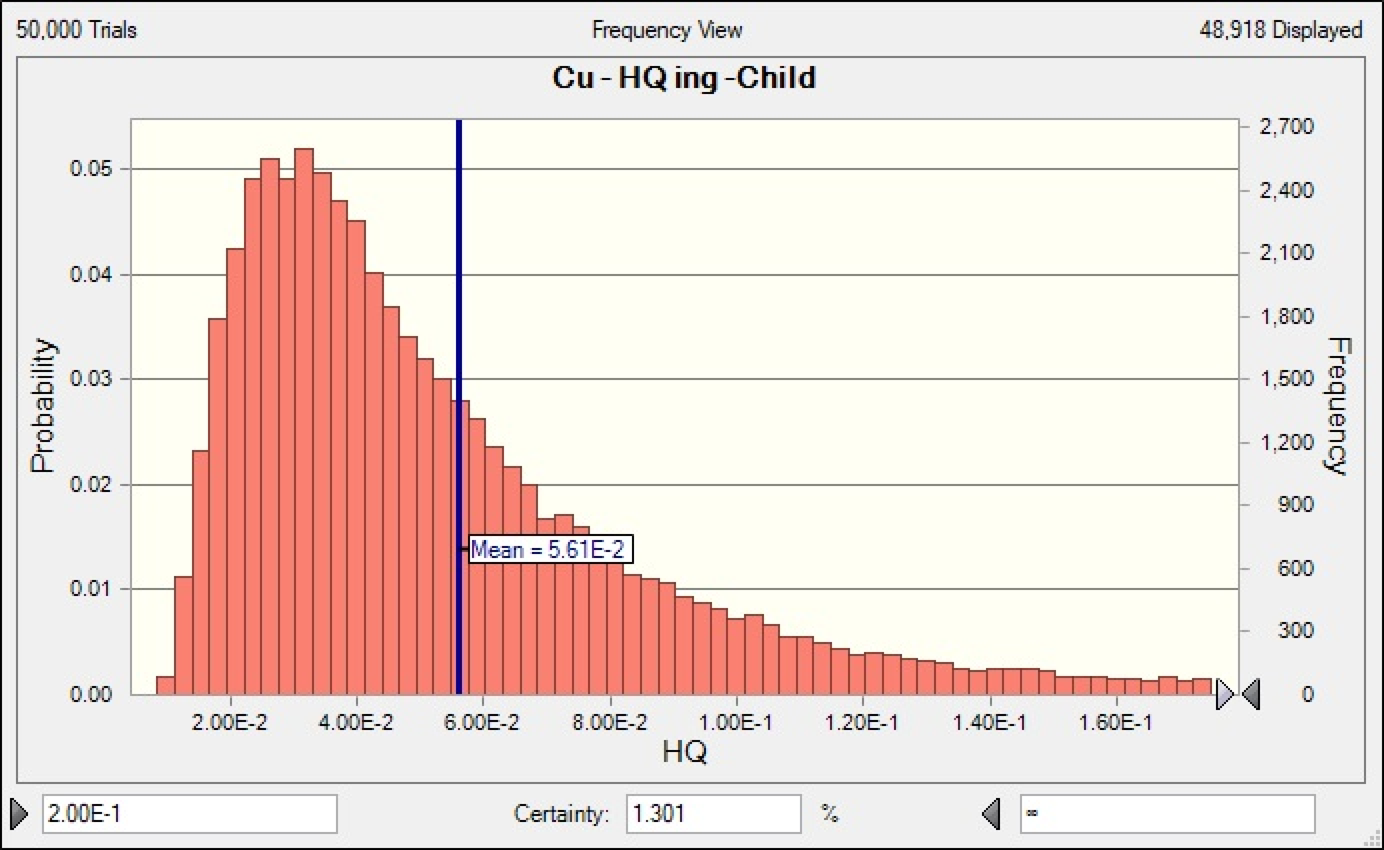

50,000 Trials
Frequency View
48,918 Displayed
Cu - HQ ing -Child
Probability
Frequency
0.05
0.04
0.03
0.02
0.01
0.00
2,700
2,400
2,100
1,800
1,500
1,200
900
600
300
0
Mean = 5.61E-2
2.00E-2
4.00E-2
6.00E-2
8.00E-2
1.00E-1
1.20E-1
1.40E-1
1.60E-1
HQ
2.00E-1
Certainty:
1.301
%
∞

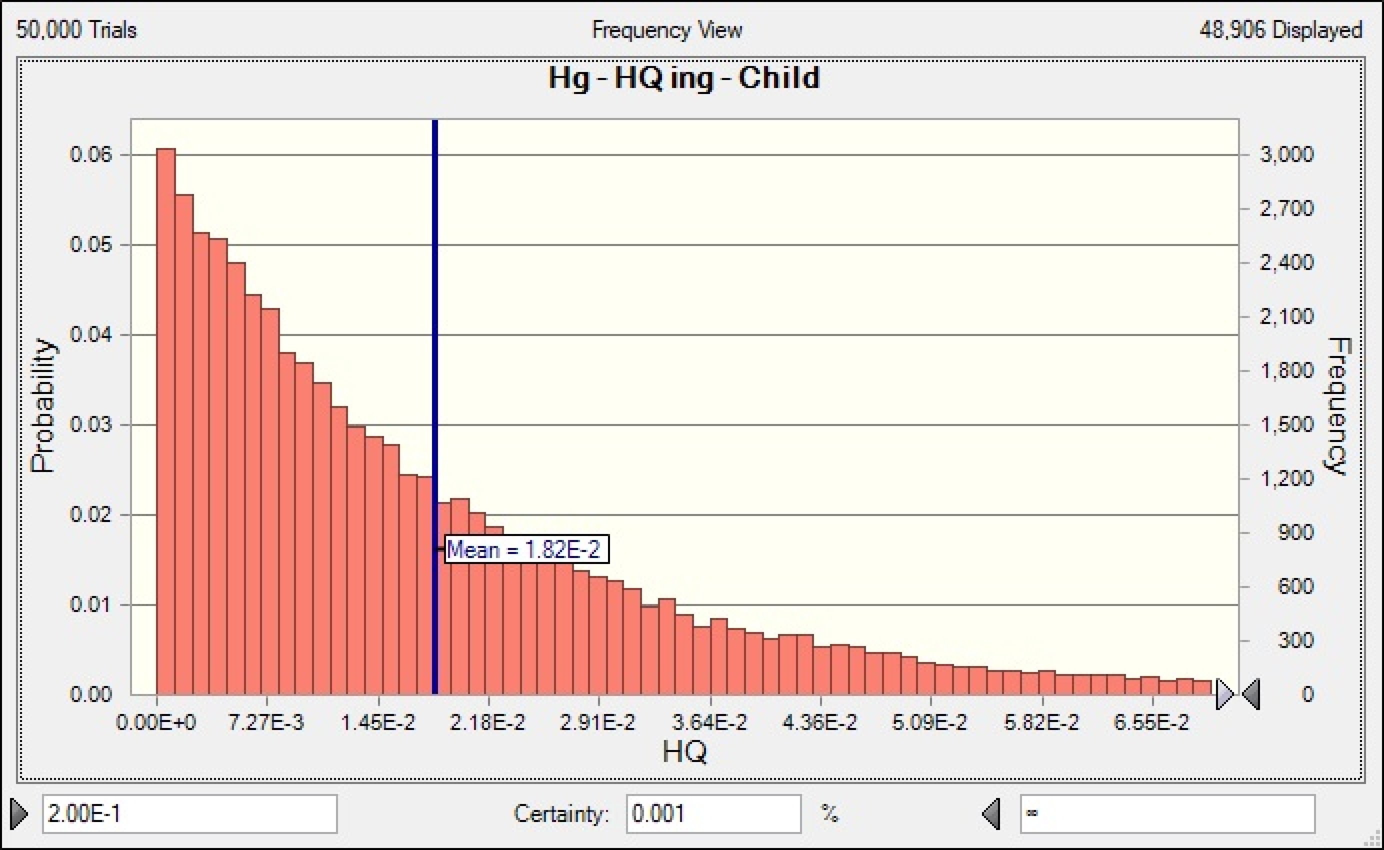
50,000 Trials
Frequency View
48,906 Displayed
Hg - HQ ing - Child
Probability
Frequency
0.06
0.05
0.04
0.03
0.02
0.01
0.00
3,000
2,700
2,400
2,100
1,800
1,500
1,200
900
600
300
0
Mean = 1.82E-2
0.00E+0
7.27E-3
1.45E-2
2.18E-2
2.91E-2
3.64E-2
4.36E-2
5.09E-2
5.82E-2
6.55E-2
HQ
2.00E-1
Certainty:
0.001
%
∞

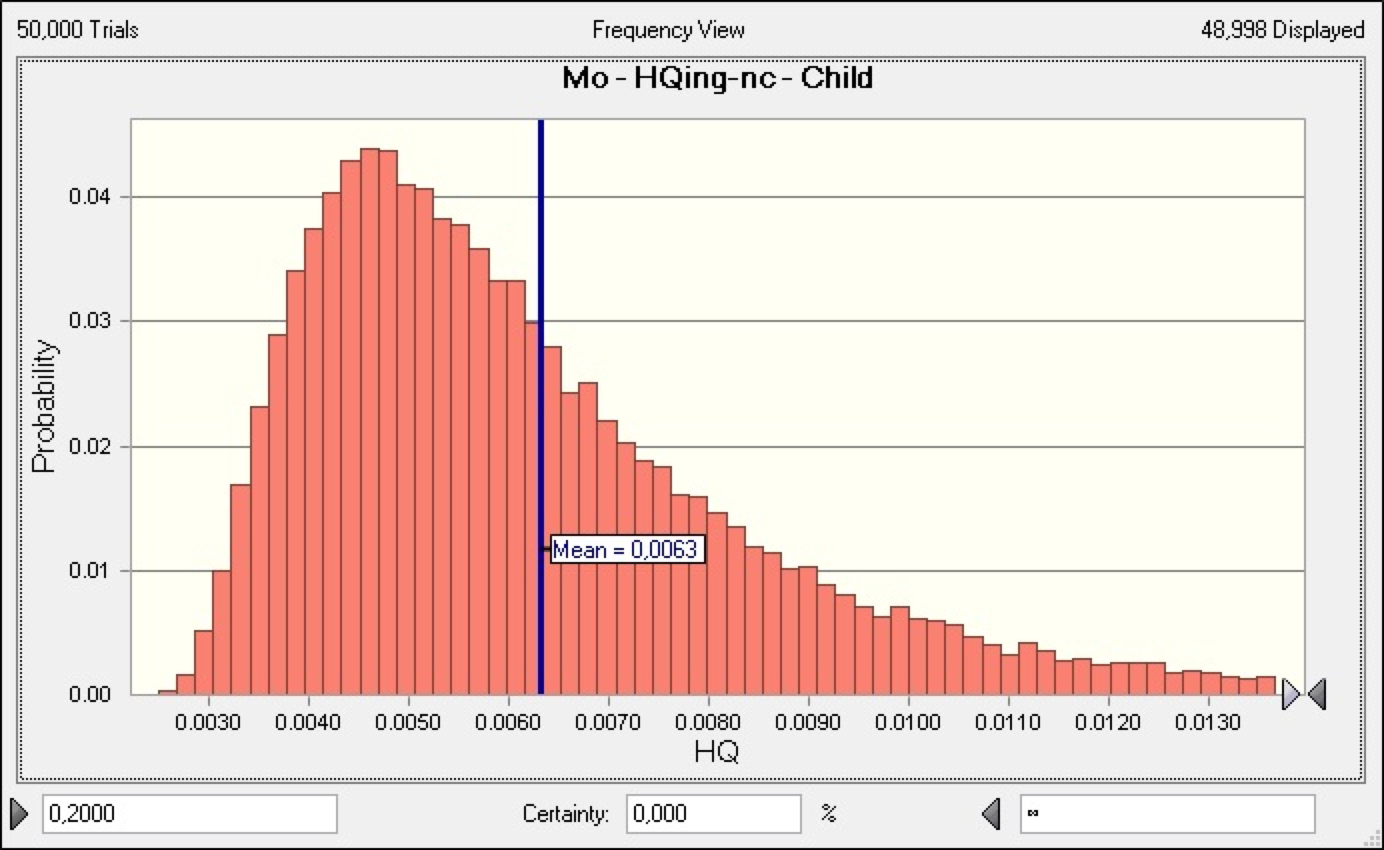
50,000 Trials
Frequency View
48,998 Displayed
Mo - HQing-nc - Child
Probability
0.00
0.01
0.02
0.03
0.04
Mean = 0,0063
0.0030
0.0040
0.0050
0.0060
0.0070
0.0080
0.0090
0.0100
0.0110
0.0120
0.0130
HQ
0,2000
Certainty:
0,000
%
∞

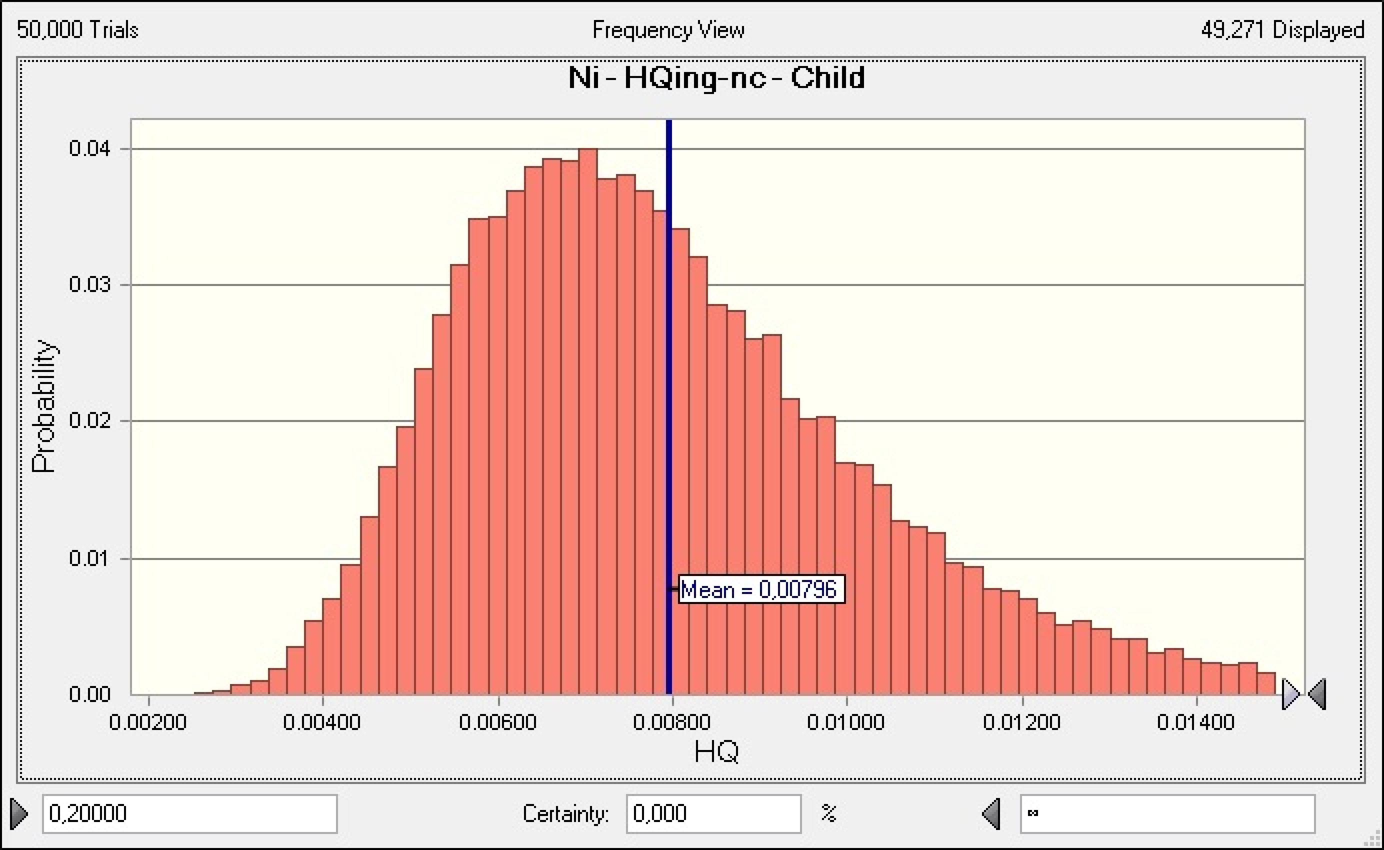
50,000 Trials
Frequency View
49,271 Displayed
Ni - HQing-nc - Child
Probability
0.04
0.03
0.02
0.01
0.00
Mean = 0,00796
0.00200
0.00400
0.00600
0.00800
0.01000
0.01200
0.01400
HQ
0,20000
Certainty:
0,000
%
∞

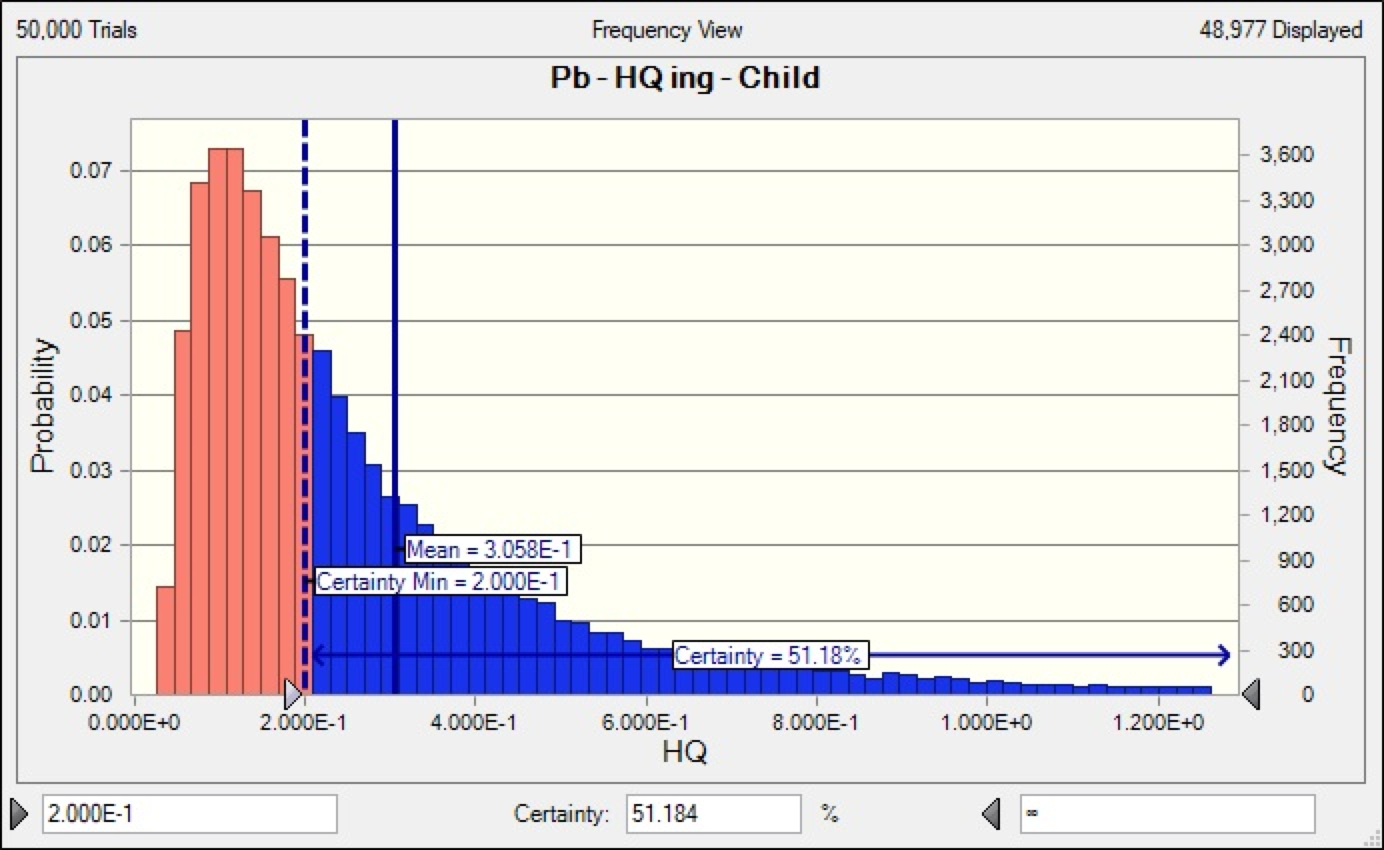
50,000 Trials
Frequency View
48,977 Displayed
Pb - HQ ing - Child
Probability
Frequency
0.07
0.06
0.05
0.04
0.03
0.02
0.01
0.00
3,600
3,300
3,000
2,700
2,400
2,100
1,800
1,500
1,200
900
600
300
0
Mean = 3.058E-1
Certainty Min = 2.000E-1
Certainty = 51.18%
0.000E+0
2.000E-1
4.000E-1
6.000E-1
8.000E-1
1.000E+0
1.200E+0
HQ
2.000E-1
Certainty:
51.184
%
∞

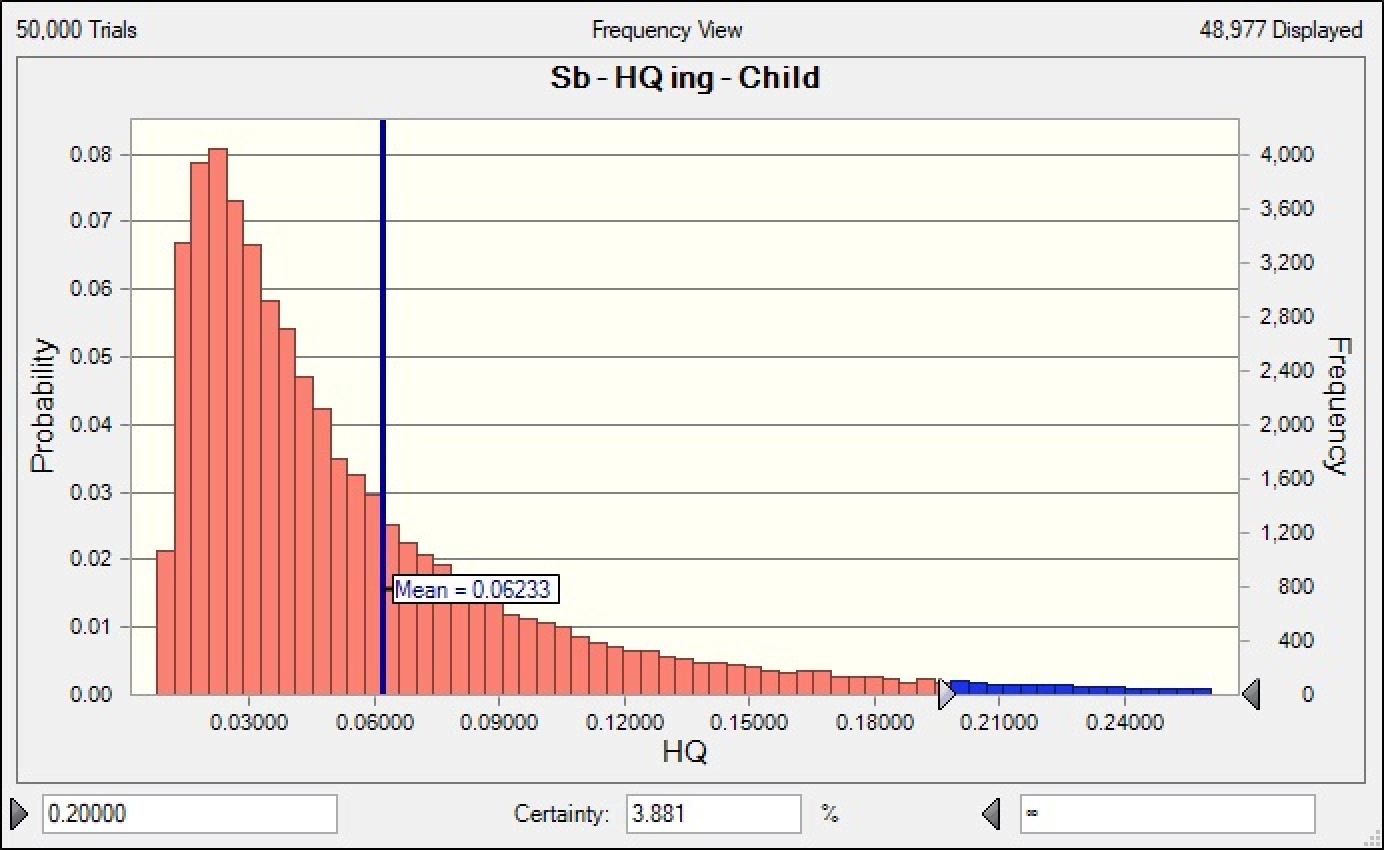

50,000 Trials
Frequency View
48,977 Displayed
Sb - HQ ing - Child
Probability
Frequency
0.00
0.01
0.02
0.03
0.04
0.05
0.06
0.07
0.08
0
400
800
1,200
1,600
2,000
2,400
2,800
3,200
3,600
4,000
Mean = 0.06233
0.03000
0.06000
0.09000
0.12000
0.15000
0.18000
0.21000
0.24000
HQ
0.20000
Certainty:
3.881
%
∞

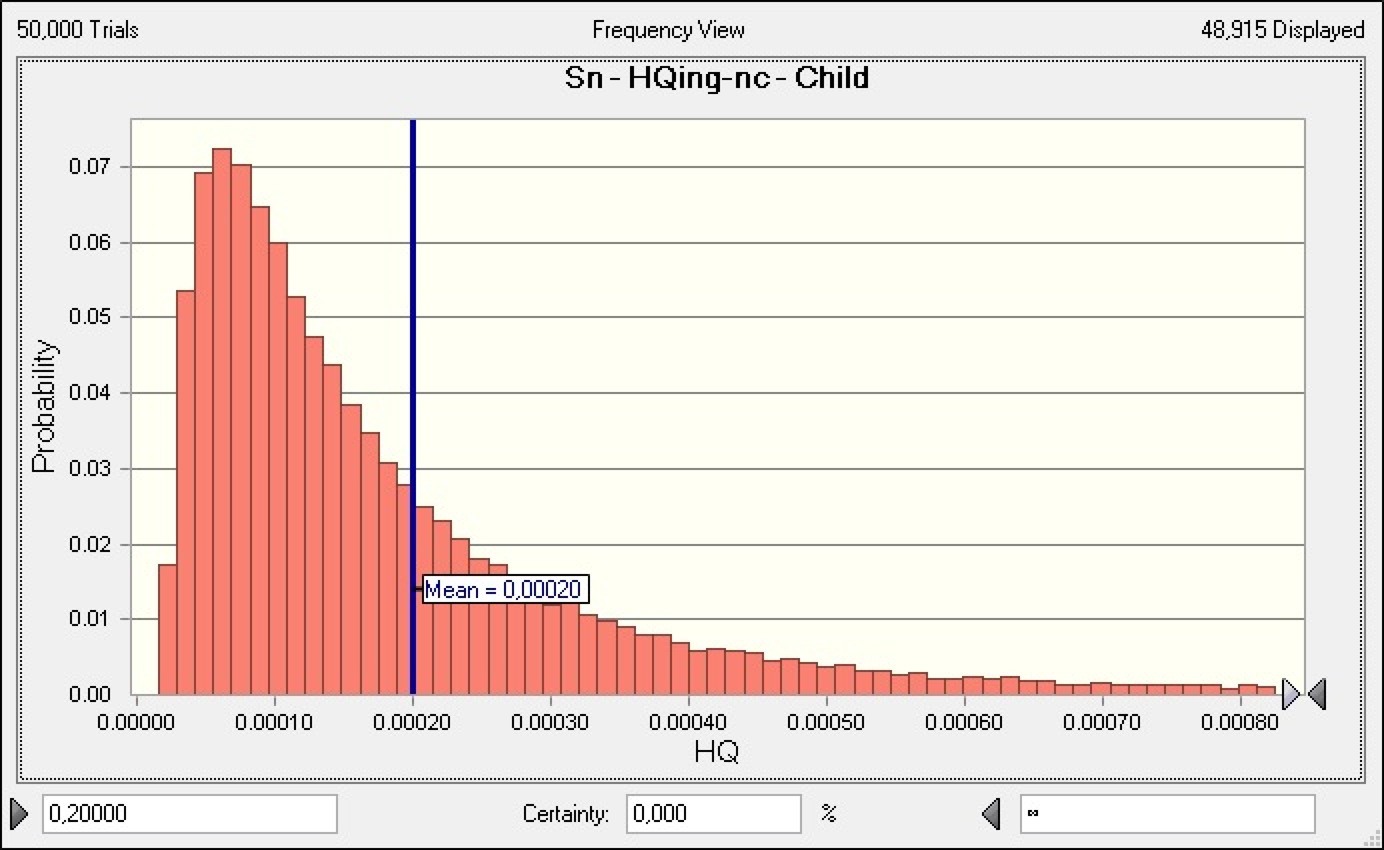

50,000 Trials
Frequency View
48,915 Displayed
Sn - HQing-nc - Child
Probability
0.07
0.06
0.05
0.04
0.03
0.02
0.01
0.00
Mean = 0,00020
0.00000
0.00010
0.00020
0.00030
0.00040
0.00050
0.00060
0.00070
0.00080
HQ
0,20000
Certainty:
0,000
%
∞

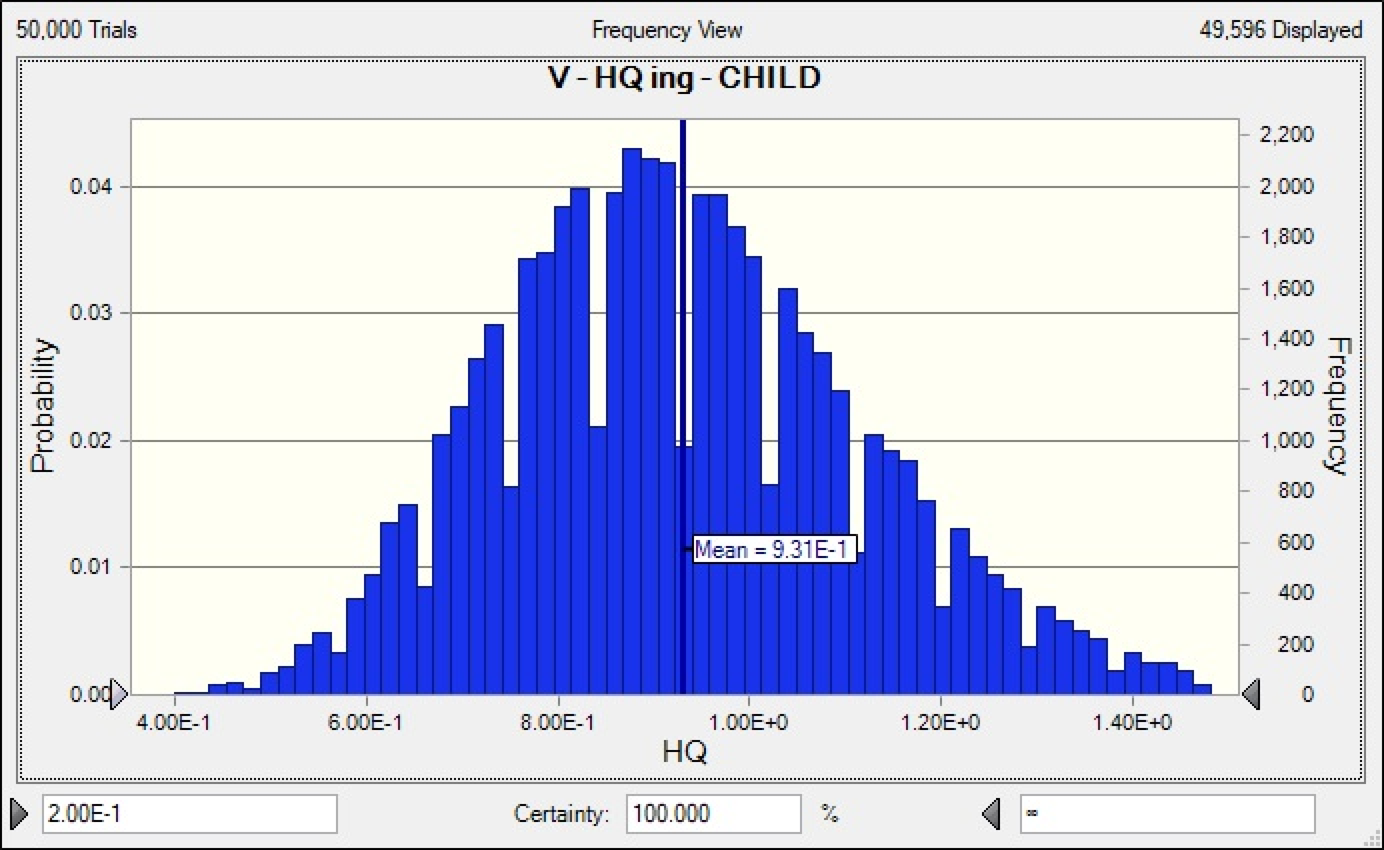
50,000 Trials
Frequency View
49,596 Displayed
V - HQ ing - CHILD
Probability
0.04
0.03
0.02
0.01
0.00
Frequency
2,200
2,000
1,800
1,600
1,400
1,200
1,000
800
600
400
200
0
Mean = 9.31E-1
4.00E-1
6.00E-1
8.00E-1
1.00E+0
1.20E+0
1.40E+0
HQ
2.00E-1
Certainty:
100.000
%
∞

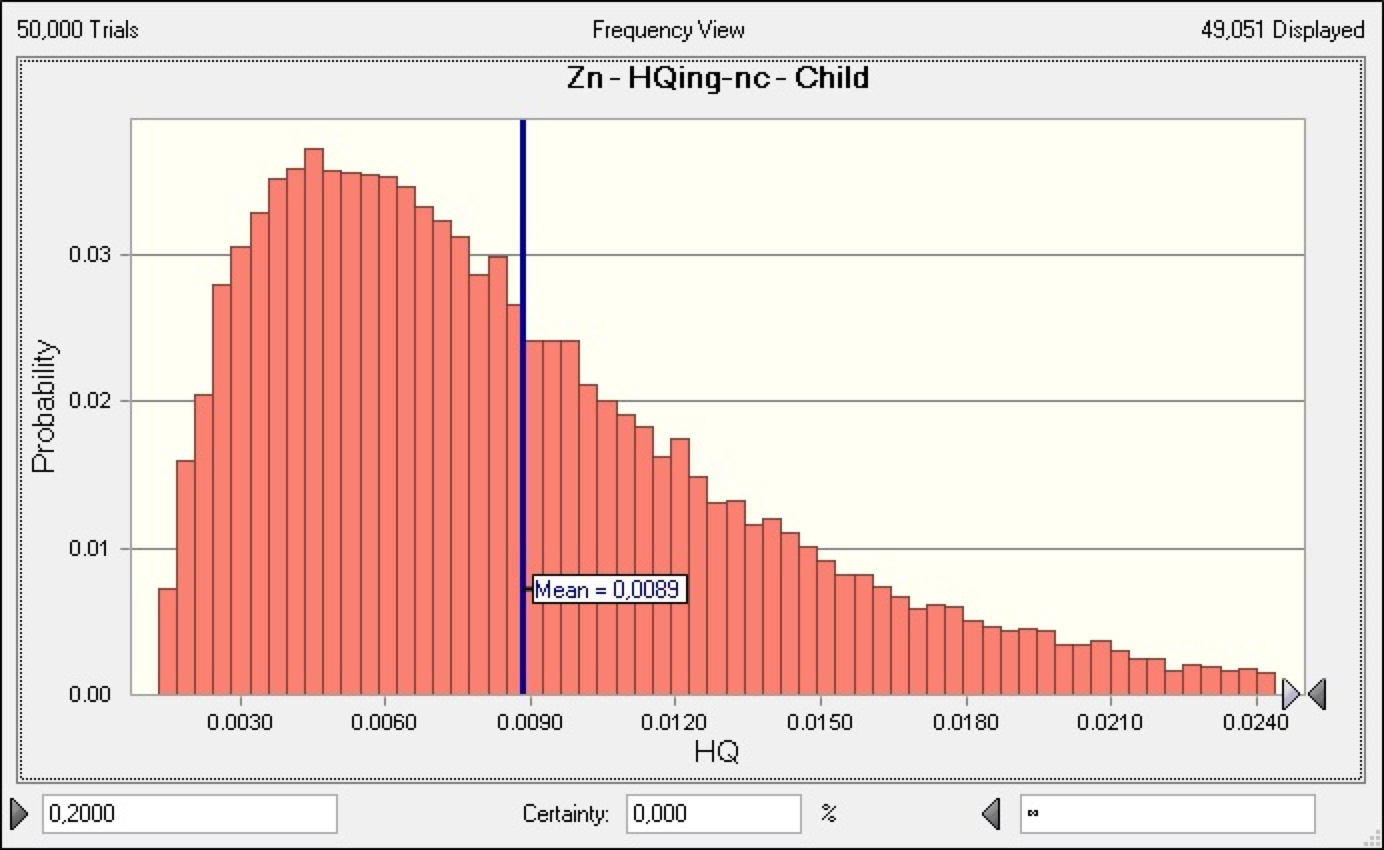
50,000 Trials
Frequency View
49,051 Displayed
Zn - HQing-nc - Child
Probability
0.03
0.02
0.01
0.00
Mean = 0,0089
0.0030
0.0060
0.0090
0.0120
0.0150
0.0180
0.0210
0.0240
HQ
0,2000
Certainty:
0,000
%
∞

HQs INHALATION
CHILDREN

HQ inh- Child

Element	**As**	**Be**	**Cd**	**Co**	**Hg**	**Mo**	**Ni**	**Sb**
Level of certainty	**0.00%**	**0.00%**	**0.00%**	**0.00%**	**0.00%**	**0.00%**	**0.00%**	**0.00%**
Mean	2.13E-04	3.13E-06	6.67E-06	3.25E-04	1.35E-07	1.11E-06	2.48E-05	1.16E-06
Median	2.16E-04	3.13E-06	5.43E-06	3.25E-04	9.28E-08	9.93E-07	2.35E-05	7.55E-07
Min	4.19E-05	-1.94E-06	1.14E-06	-8.79E-05	2.28E-12	4.38E-07	5.56E-06	1.50E-07
Max	3.57E-04	8.55E-06	1.06E-04	7.27E-04	1.67E-06	7.13E-06	7.91E-05	3.72E-05
St. Dev.	4.20E-05	1.20E-06	4.62E-06	7.47E-05	1.35E-07	4.55E-07	7.71E-06	1.33E-06

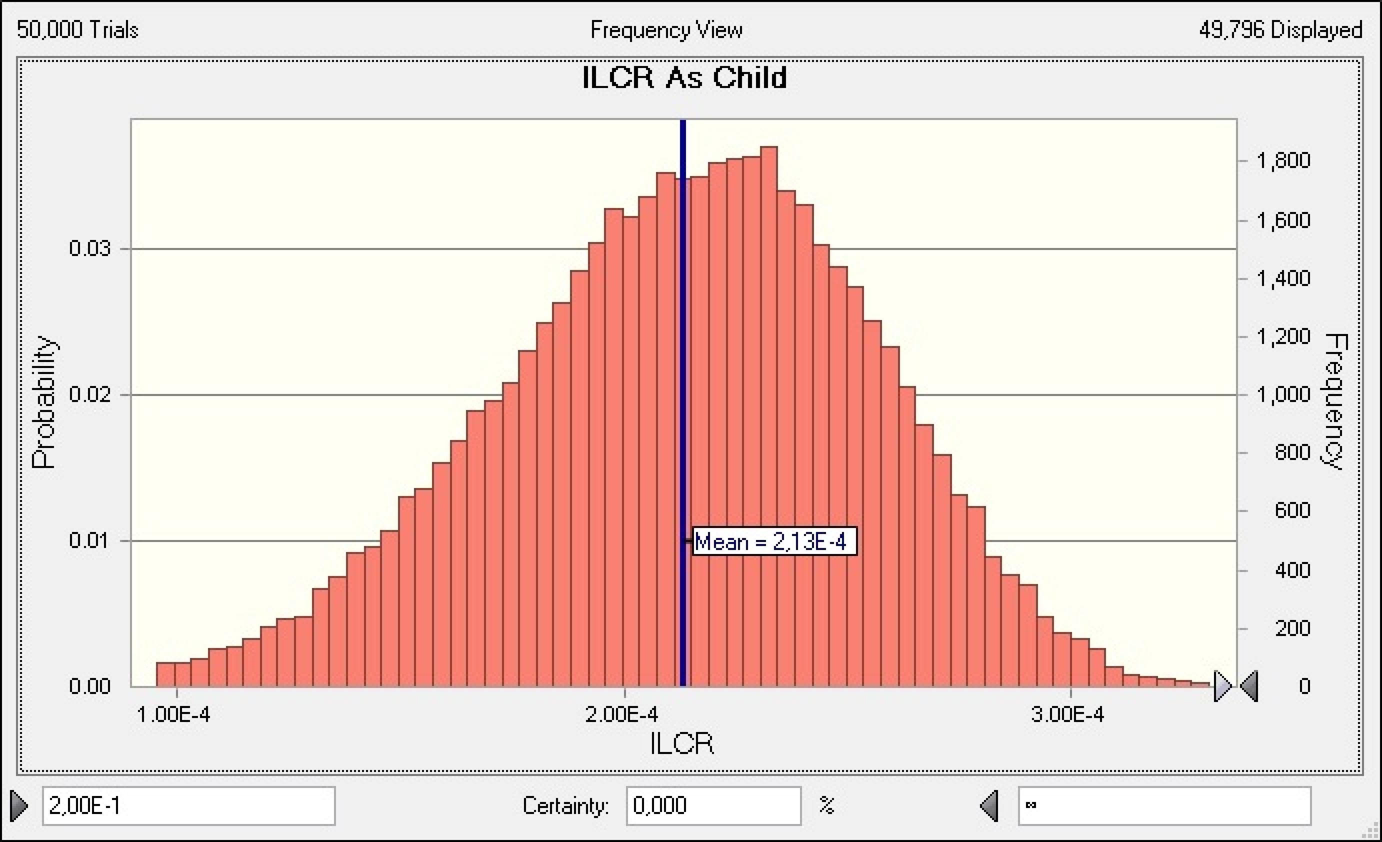
50,000 Trials
Frequency View
49,796 Displayed
ILCR As Child
Probability
0.03
0.02
0.01
0.00
1,800
1,600
1,400
1,200
1,000
800
600
400
200
0
Frequency
Mean = 2,13E-4
1.00E-4
2.00E-4
3.00E-4
ILCR
2,00E-1
Certainty:
0,000
%
∞

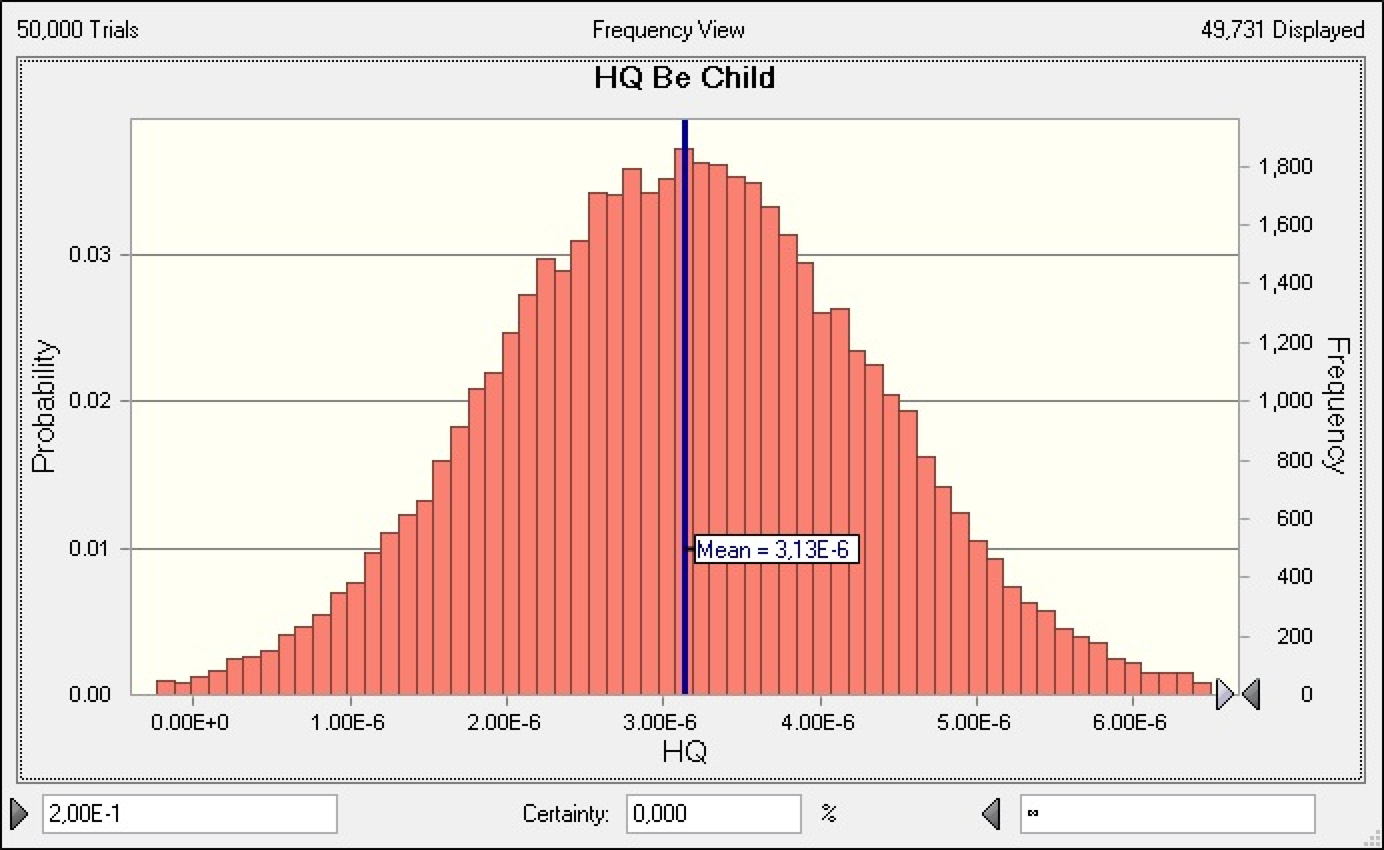
50,000 Trials
Frequency View
49,731 Displayed
HQ Be Child
Probability
Frequency
0.03
0.02
0.01
0.00
1,800
1,600
1,400
1,200
1,000
800
600
400
200
0
Mean = 3,13E-6
0.00E+0
1.00E-6
2.00E-6
3.00E-6
4.00E-6
5.00E-6
6.00E-6
HQ
2,00E-1
Certainty:
0,000
%
∞

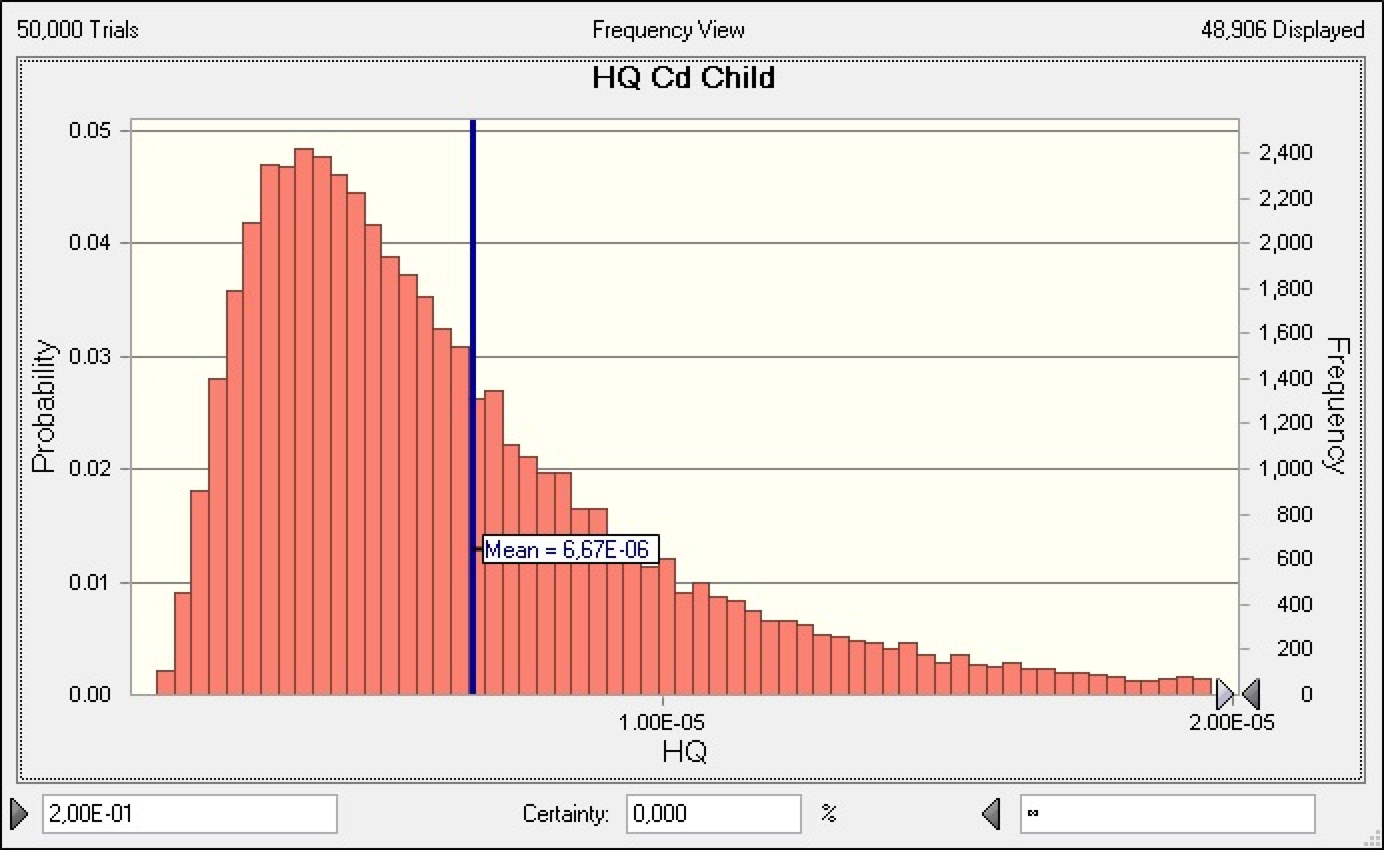

50,000 Trials
Frequency View
48,906 Displayed
HQ Cd Child
Probability
0.05
0.04
0.03
0.02
0.01
0.00
Frequency
2,400
2,200
2,000
1,800
1,600
1,400
1,200
1,000
800
600
400
200
0
Mean = 6,67E-06
1.00E-05
2.00E-05
HQ
2,00E-01
Certainty:
0,000
%
∞

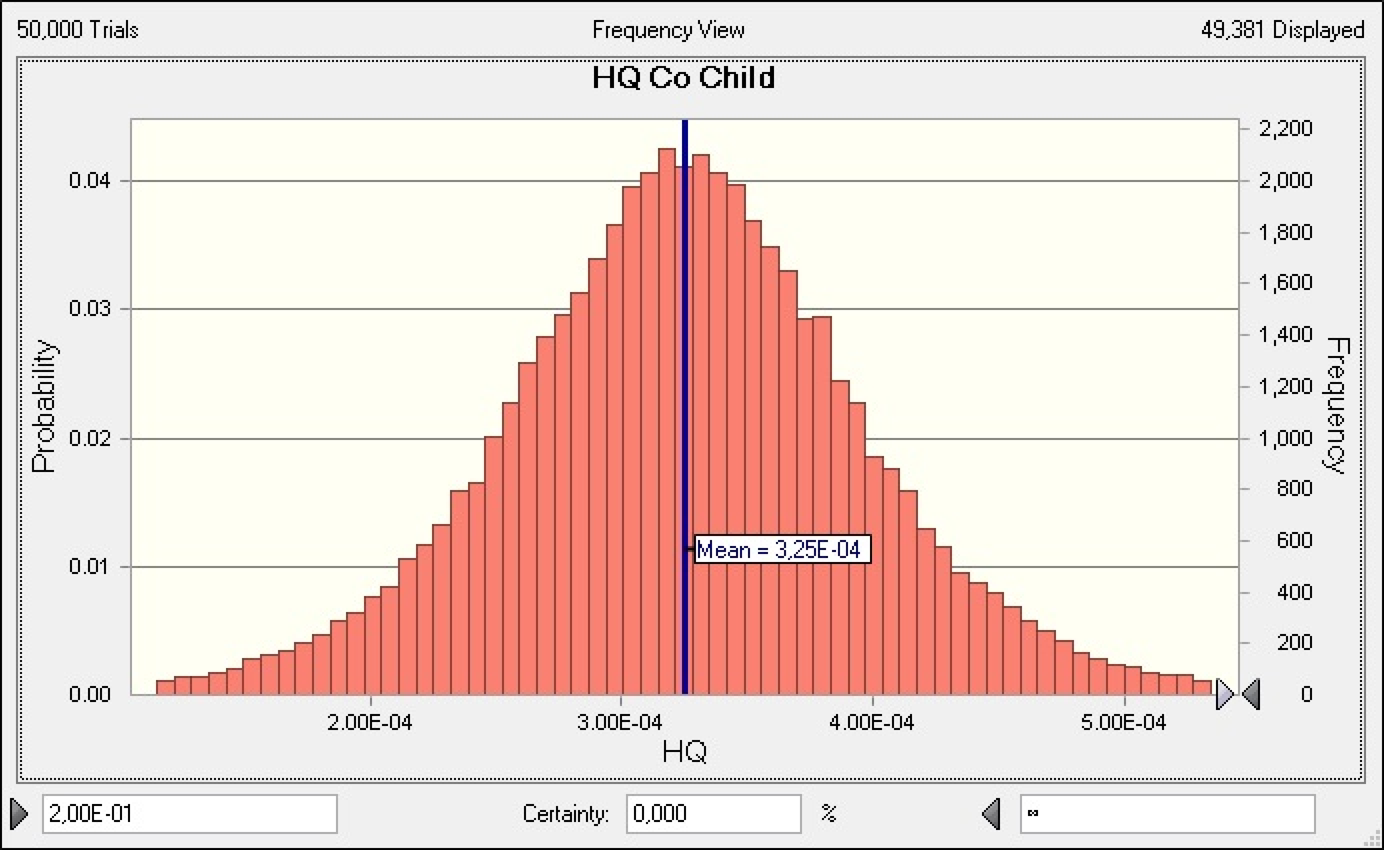
50,000 Trials
Frequency View
49,381 Displayed
HQ Co Child
Probability
Frequency
0.00
0.01
0.02
0.03
0.04
0
200
400
600
800
1,000
1,200
1,400
1,600
1,800
2,000
2,200
Mean = 3,25E-04
2.00E-04
3.00E-04
4.00E-04
5.00E-04
HQ
2,00E-01
Certainty:
0,000
%
∞

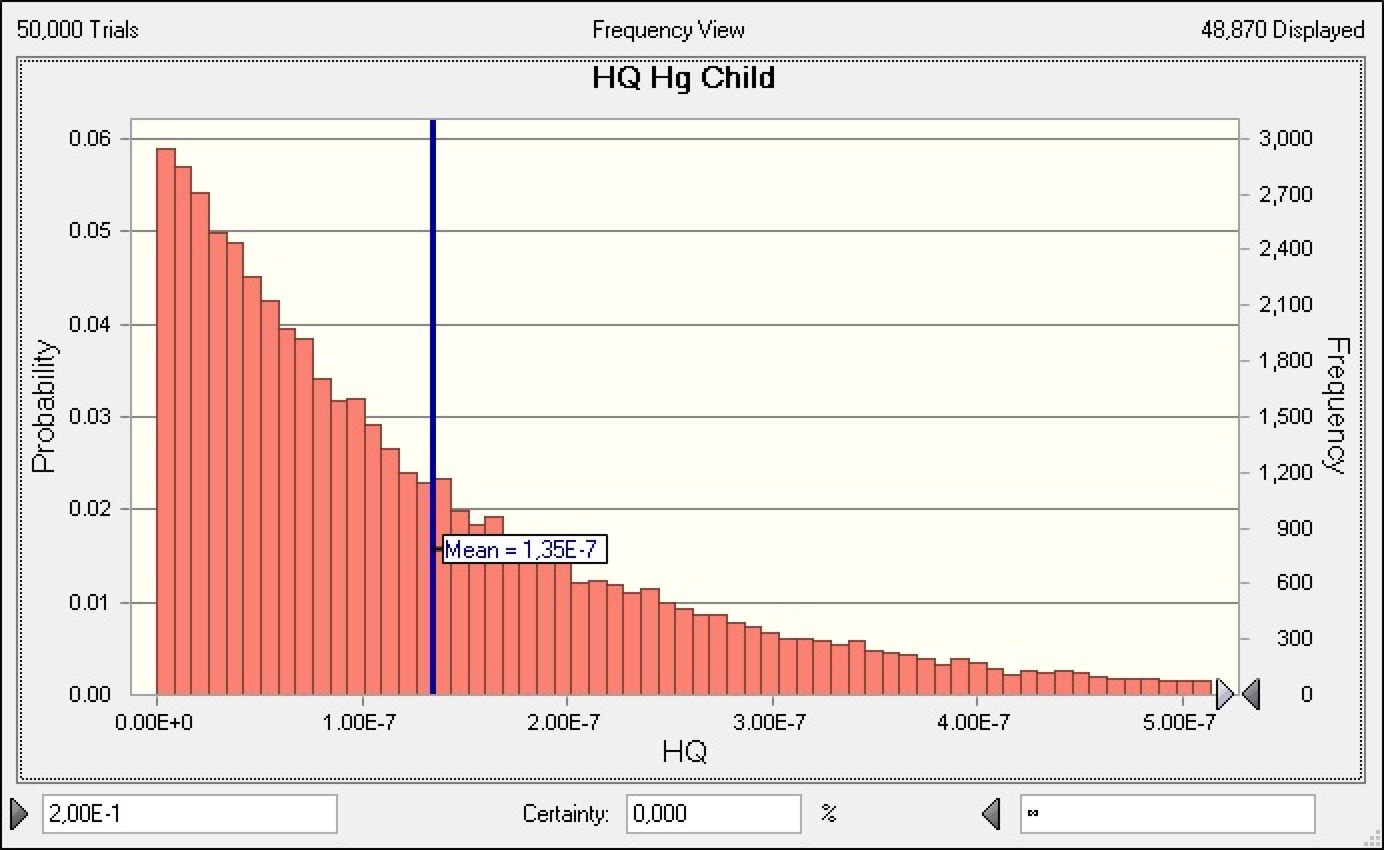

50,000 Trials
Frequency View
48,870 Displayed
HQ Hg Child
Probability
Frequency
0.06
0.05
0.04
0.03
0.02
0.01
0.00
3,000
2,700
2,400
2,100
1,800
1,500
1,200
900
600
300
0
Mean = 1,35E-7
0.00E+0
1.00E-7
2.00E-7
3.00E-7
4.00E-7
5.00E-7
HQ
2,00E-1
Certainty:
0,000
%
∞

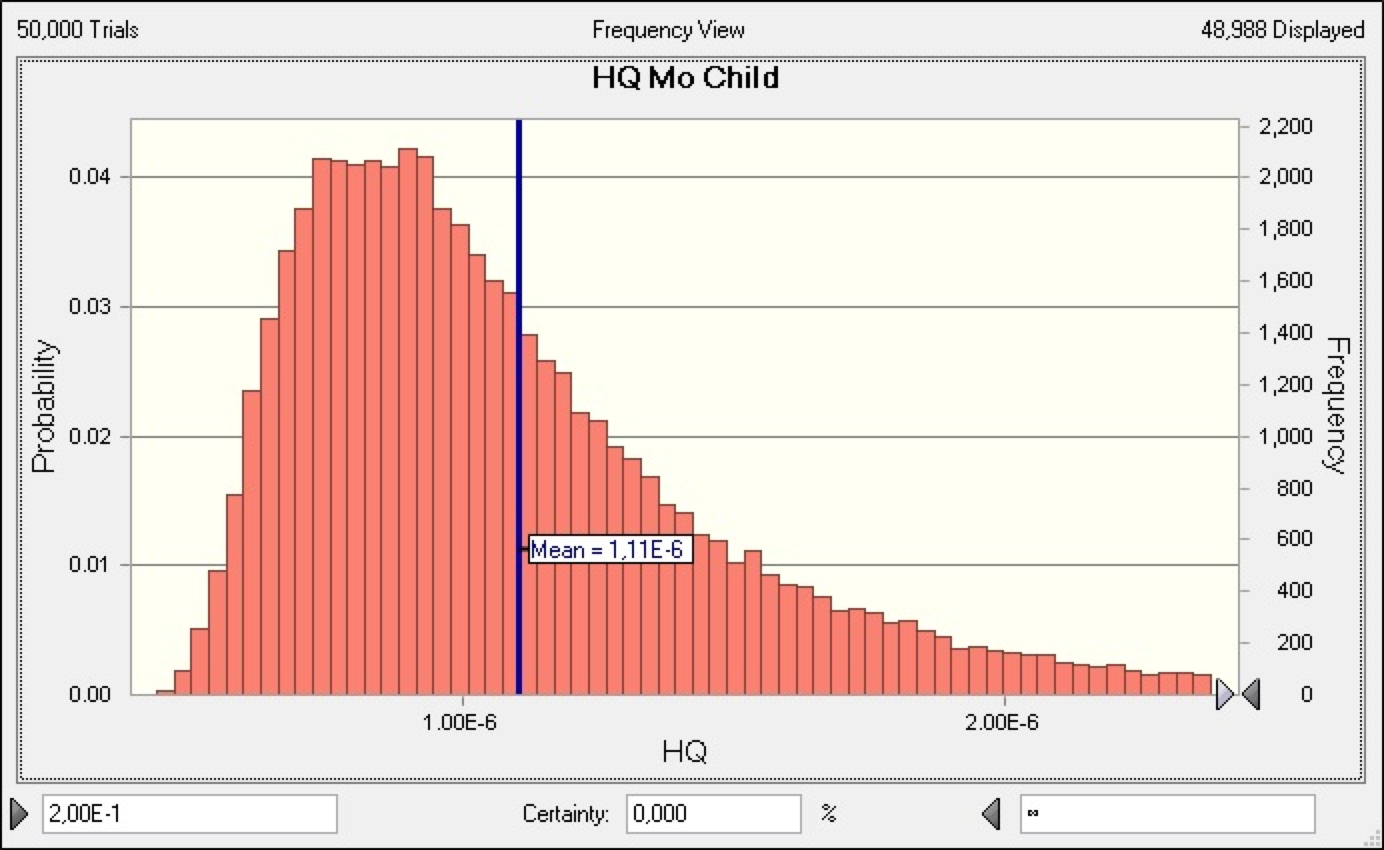

50,000 Trials
Frequency View
48,988 Displayed
HQ Mo Child
Probability
0.04
0.03
0.02
0.01
0.00
Frequency
2,200
2,000
1,800
1,600
1,400
1,200
1,000
800
600
400
200
0
Mean = 1,11E-6
1.00E-6
2.00E-6
HQ
2,00E-1
Certainty:
0,000
%
∞

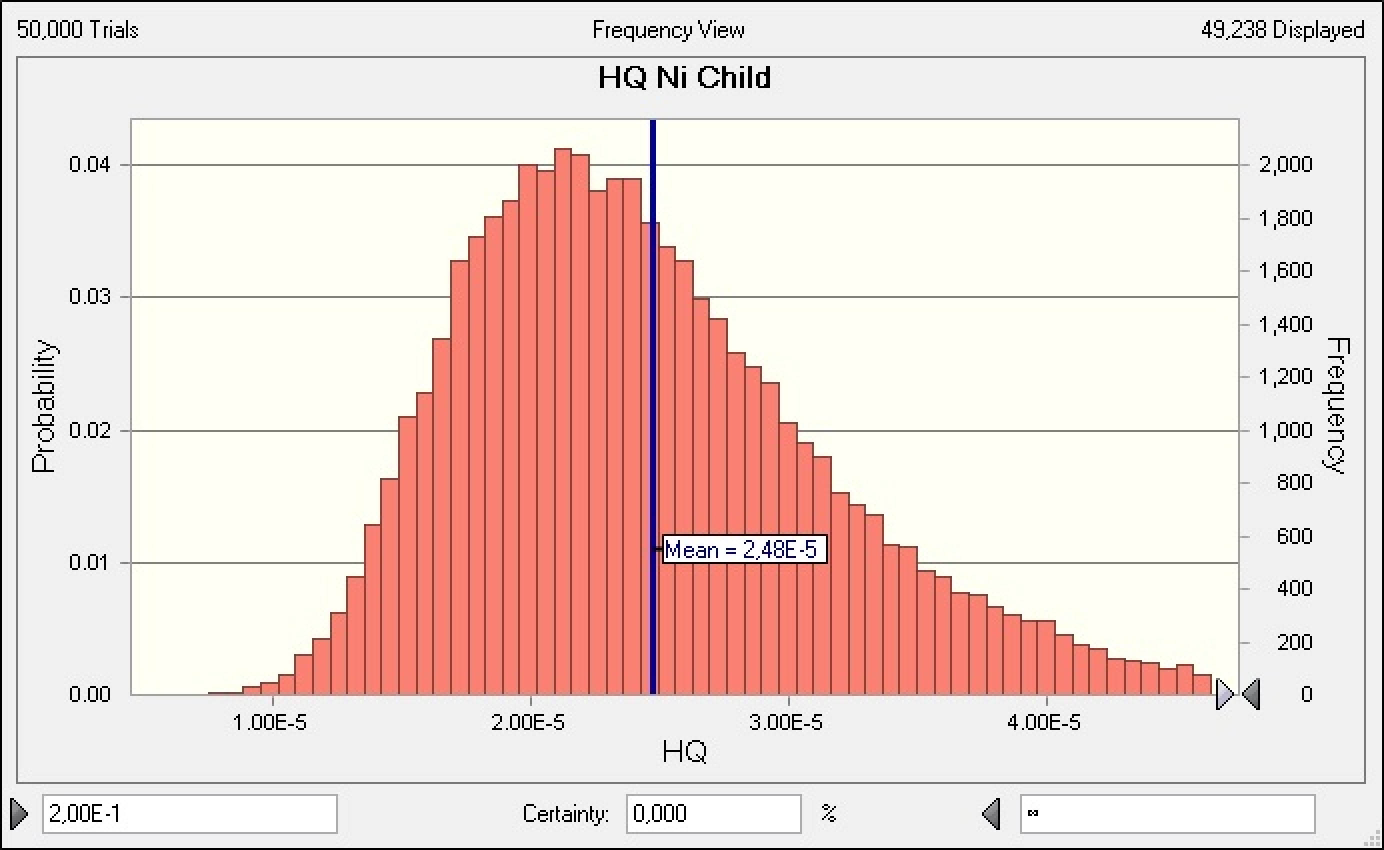

50,000 Trials
Frequency View
49,238 Displayed
HQ Ni Child
Probability
Frequency
0.04
0.03
0.02
0.01
0.00
2,000
1,800
1,600
1,400
1,200
1,000
800
600
400
200
0
Mean = 2,48E-5
1.00E-5
2.00E-5
3.00E-5
4.00E-5
HQ
2,00E-1
Certainty:
0,000
%
∞

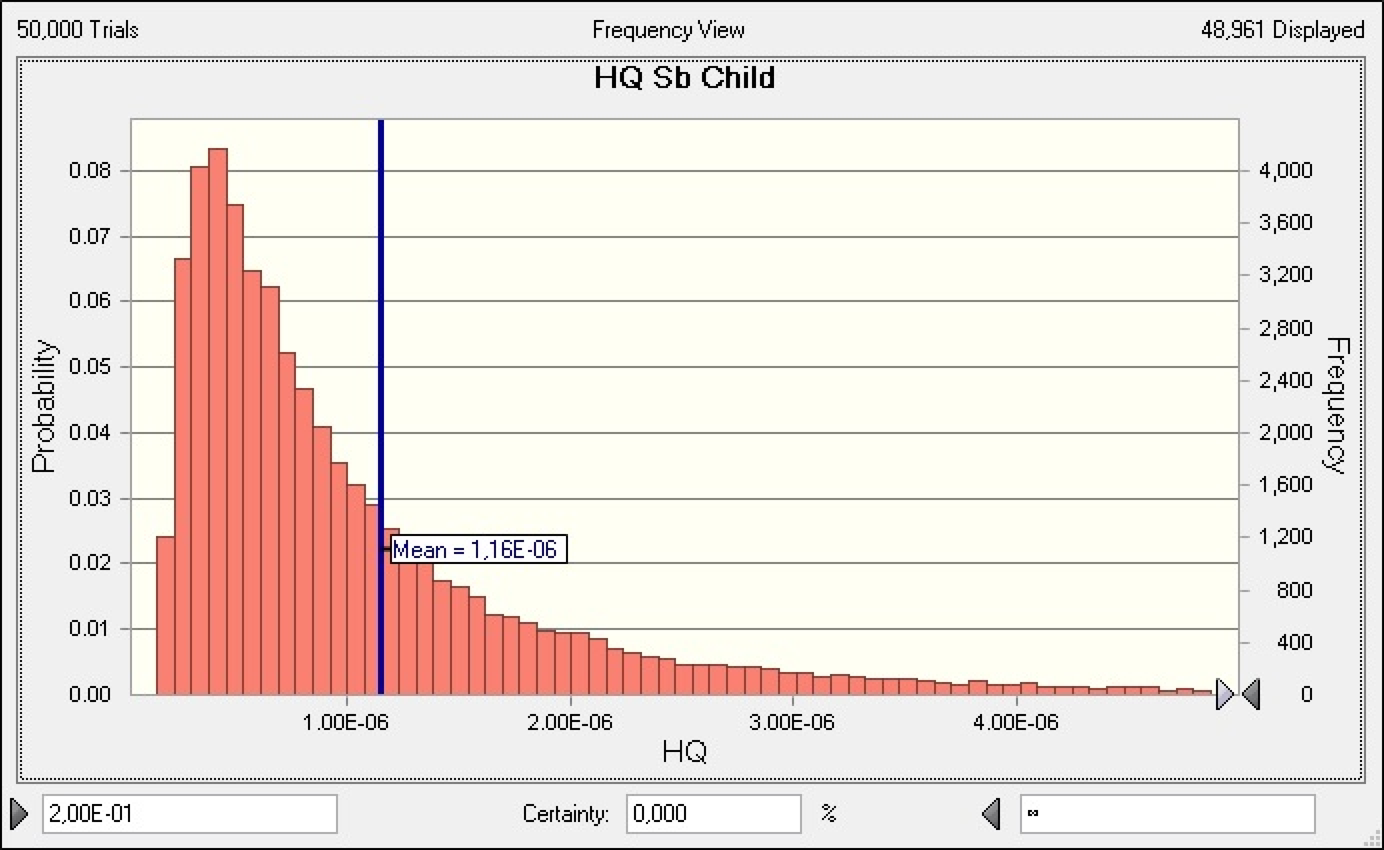
50,000 Trials
Frequency View
48,961 Displayed
HQ Sb Child
Probability
Frequency
0.08
0.07
0.06
0.05
0.04
0.03
0.02
0.01
0.00
4,000
3,600
3,200
2,800
2,400
2,000
1,600
1,200
800
400
0
Mean = 1,16E-06
1.00E-06
2.00E-06
3.00E-06
4.00E-06
HQ
2,00E-01
Certainty:
0,000
%
∞

HQs INHALATION ADULTS

HQ inh- Child

Element	**As**	**Be**	**Cd**	**Co**	**Hg**	**Mo**	**Ni**	**Sb**
Level of certainty	**0.00%**	**0.00%**	**0.00%**	**0.00%**	**0.00%**	**0.00%**	**0.00%**	**0.00%**
Mean	1.07E-03	1.56E-05	3.33E-05	1.62E-03	6.79E-07	5.53E-06	1.24E-04	5.75E.06
Median	1.08E-03	1.56E-05	2.69E-05	1.62E-03	4.73E-07	4.96E-06	1.18E-04	3.77E-06
Min	2.42E-04	-9.22E-06	5.42E-06	-3.42E-04	1.33E-11	2.15E-06	3.72E.05	7.50E-07
Max	1.70E-03	4.19E-05	4.84E-04	3.89E-03	6.33E-06	4.58E-05	3.94E-04	1.68E-04
St. Dev.	2.09E-04	5.99E-06	2.35E-05	3.73E-04	6.75E-07	2.31E-06	3.85E-05	6.48E-06

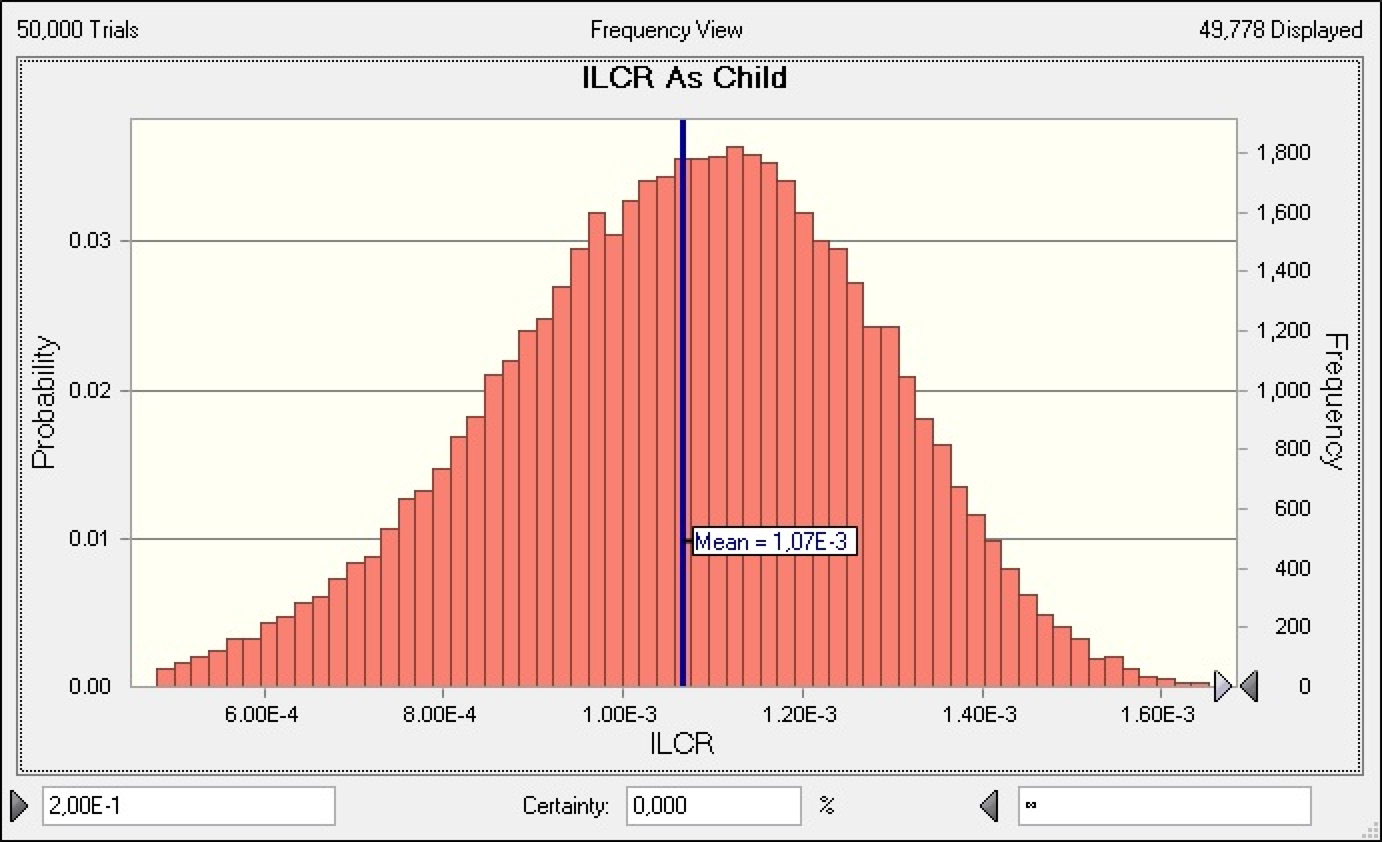
50,000 Trials
Frequency View
49,778 Displayed
ILCR As Child
Probability
Frequency
0.03
0.02
0.01
0.00
1,800
1,600
1,400
1,200
1,000
800
600
400
200
0
Mean = 1,07E-3
6.00E-4
8.00E-4
1.00E-3
1.20E-3
1.40E-3
1.60E-3
ILCR
2,00E-1
Certainty:
0,000
%
∞

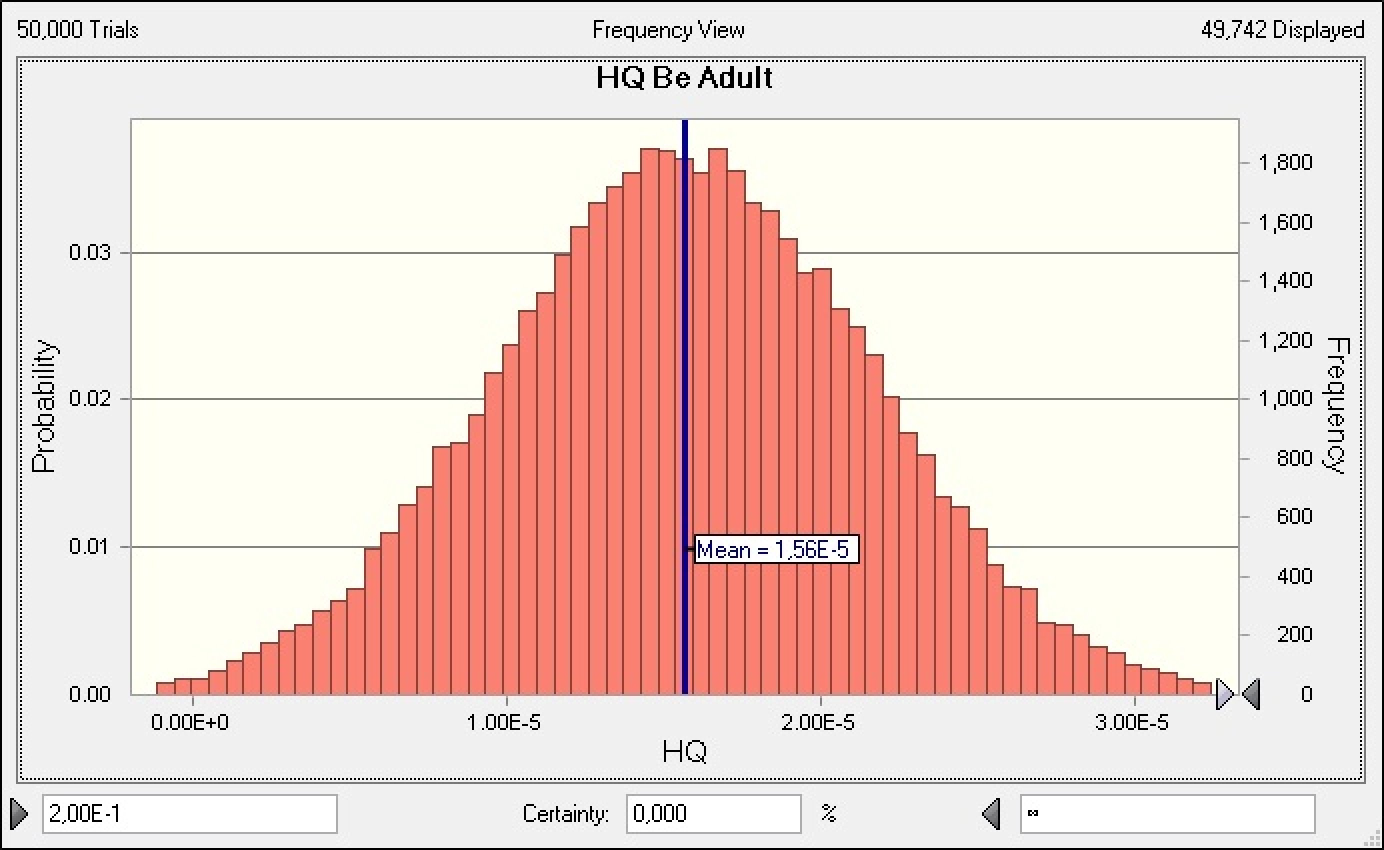

50,000 Trials
Frequency View
49,742 Displayed
HQ Be Adult
Probability
Frequency
0.00
0.01
0.02
0.03
0
200
400
600
800
1,000
1,200
1,400
1,600
1,800
Mean = 1,56E-5
0.00E+0
1.00E-5
2.00E-5
3.00E-5
HQ
2,00E-1
Certainty:
0,000
%
∞

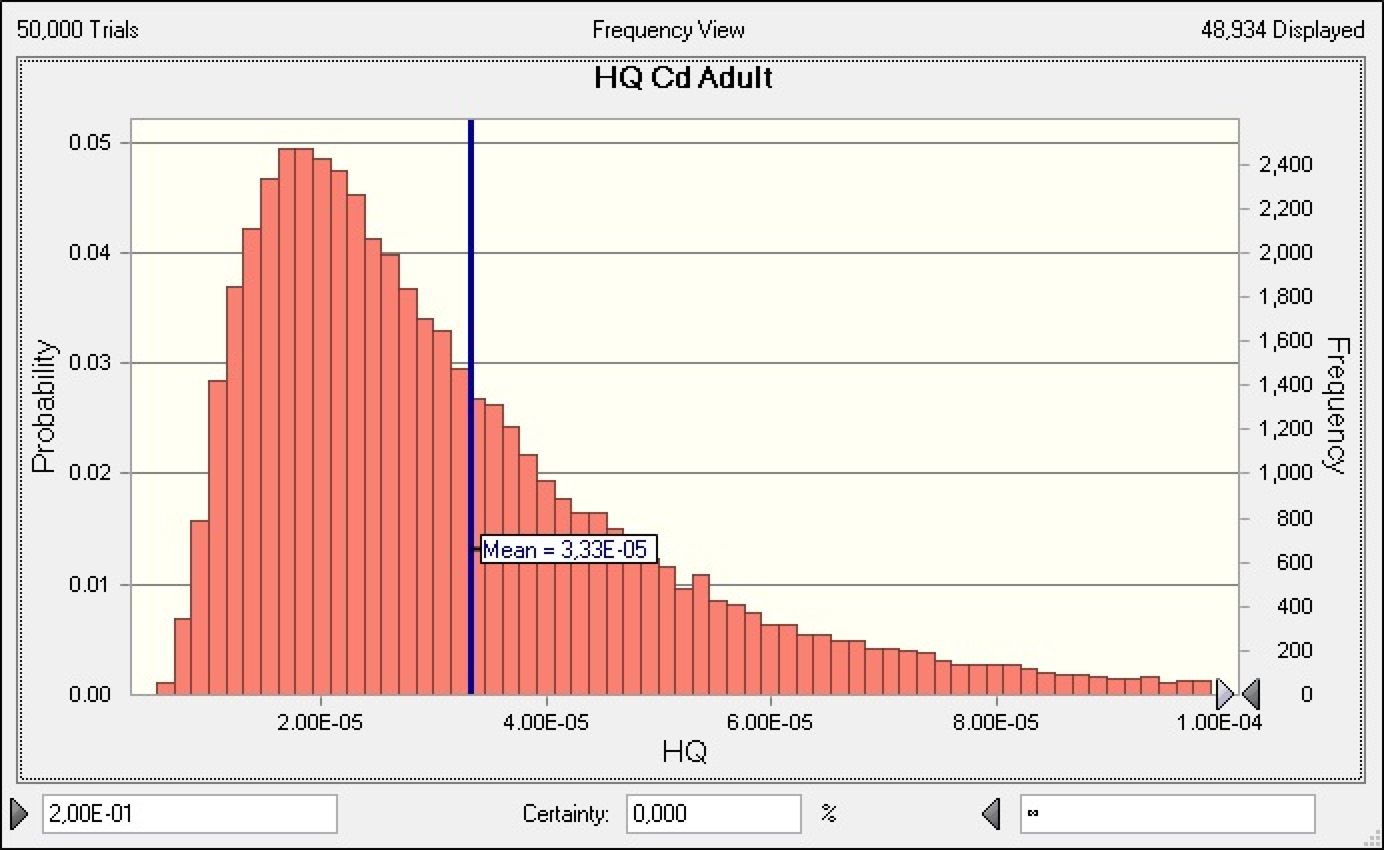

50,000 Trials
Frequency View
48,934 Displayed
HQ Cd Adult
Probability
0.05
0.04
0.03
0.02
0.01
0.00
Frequency
2,400
2,200
2,000
1,800
1,600
1,400
1,200
1,000
800
600
400
200
0
Mean = 3,33E-05
2.00E-05
4.00E-05
6.00E-05
8.00E-05
1.00E-04
HQ
2,00E-01
Certainty:
0,000
%
∞

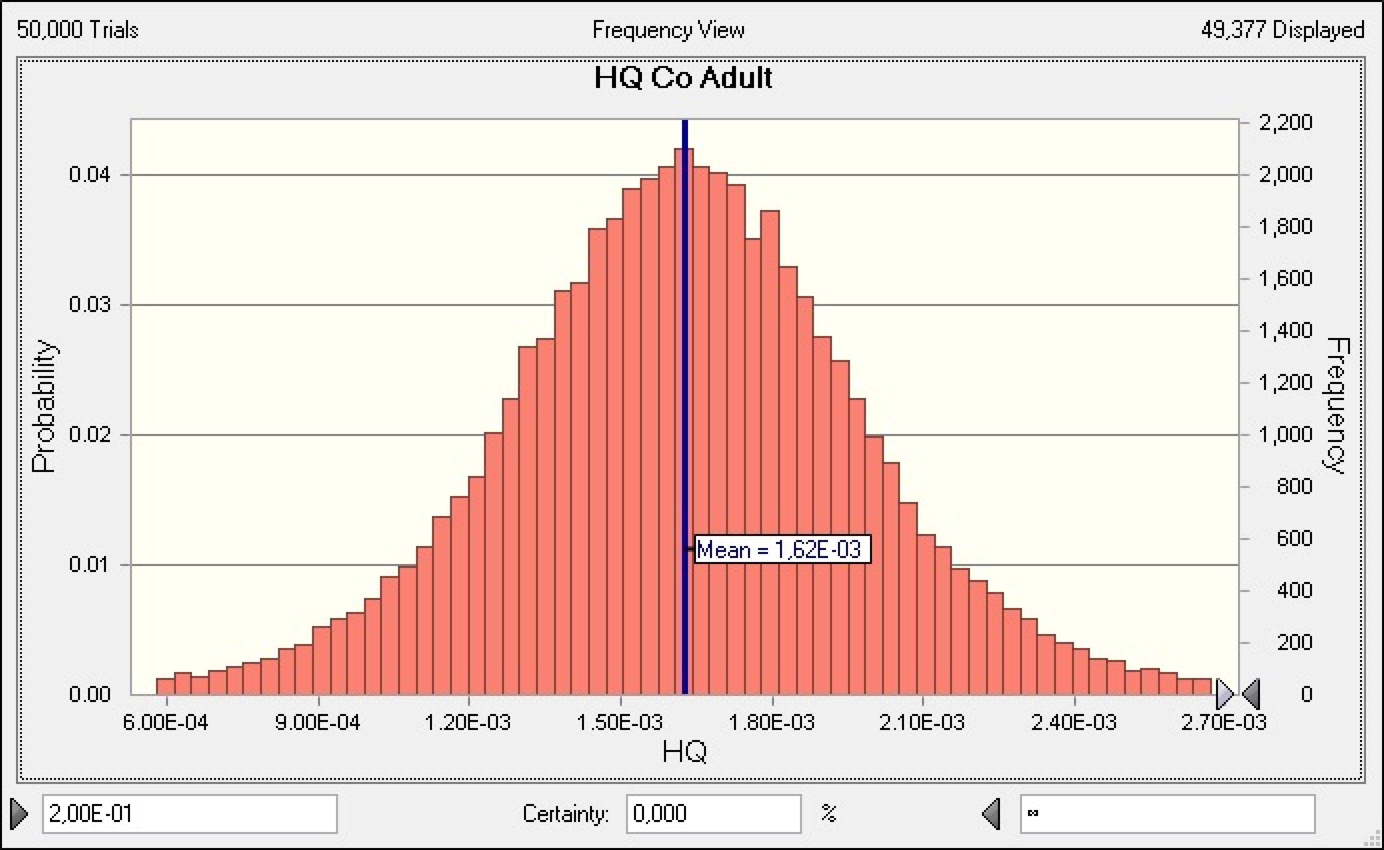
50,000 Trials
Frequency View
49,377 Displayed
HQ Co Adult
Probability
Frequency
0.04
0.03
0.02
0.01
0.00
2,200
2,000
1,800
1,600
1,400
1,200
1,000
800
600
400
200
0
Mean = 1,62E-03
6.00E-04
9.00E-04
1.20E-03
1.50E-03
1.80E-03
2.10E-03
2.40E-03
2.70E-03
HQ
2,00E-01
Certainty:
0,000
%
∞

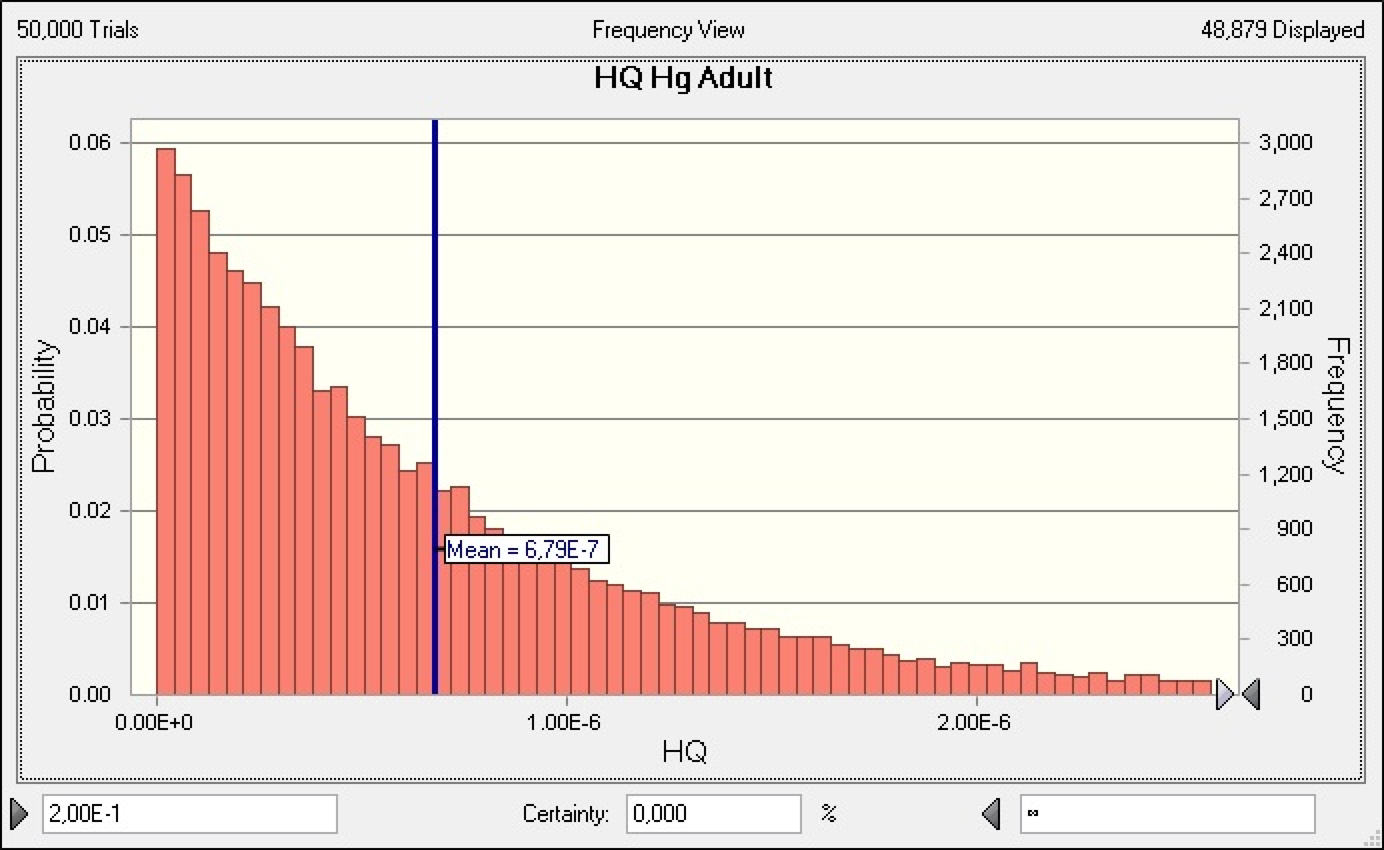
50,000 Trials
Frequency View
48,879 Displayed
HQ Hg Adult
Probability
0.06
0.05
0.04
0.03
0.02
0.01
0.00
Frequency
3,000
2,700
2,400
2,100
1,800
1,500
1,200
900
600
300
0
Mean = 6,79E-7
0.00E+0
1.00E-6
2.00E-6
HQ
2,00E-1
Certainty:
0,000
%
∞

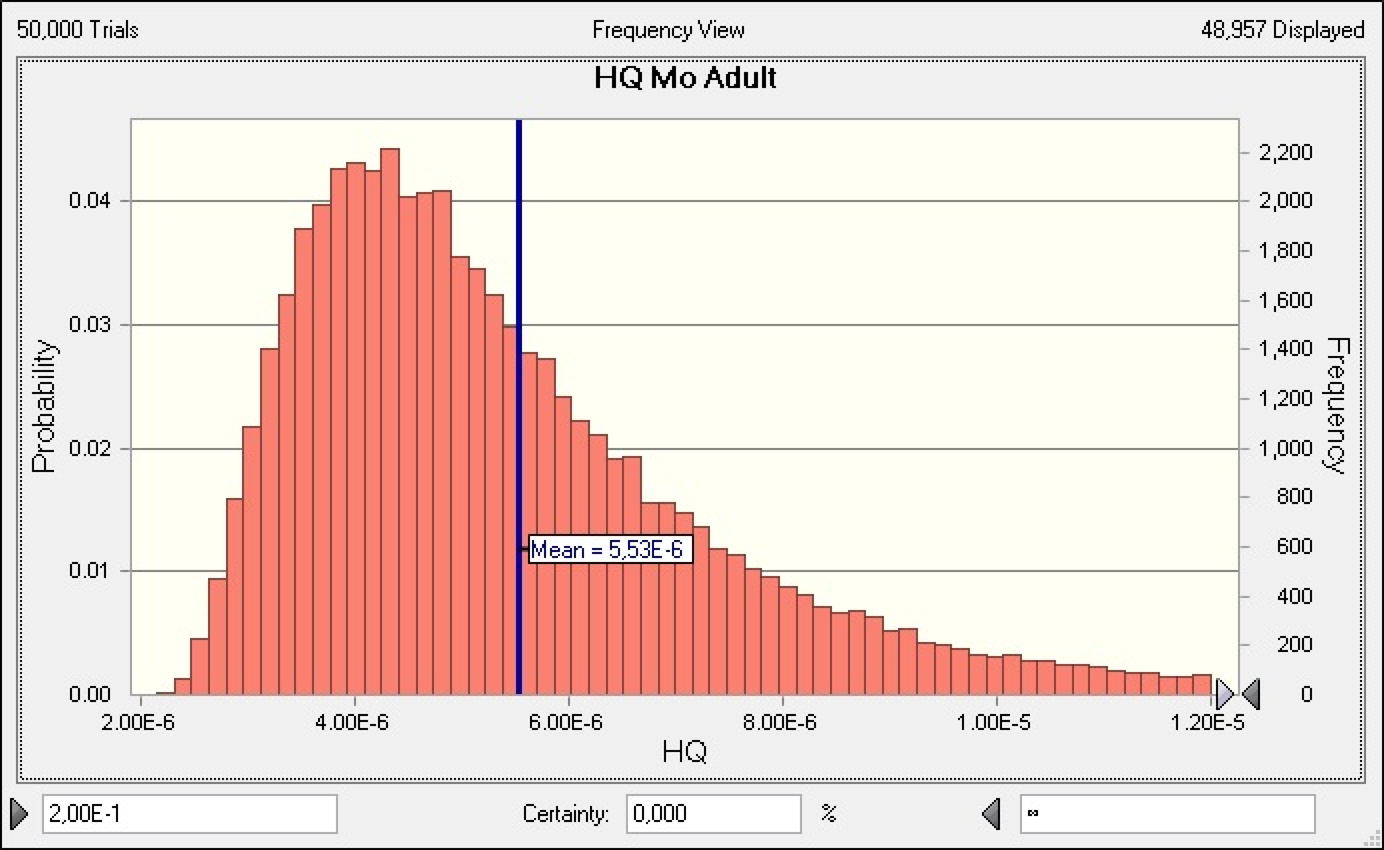
50,000 Trials
Frequency View
48,957 Displayed
HQ Mo Adult
Probability
Frequency
0.04
0.03
0.02
0.01
0.00
2,200
2,000
1,800
1,600
1,400
1,200
1,000
800
600
400
200
0
Mean = 5,53E-6
2.00E-6
4.00E-6
6.00E-6
8.00E-6
1.00E-5
1.20E-5
HQ
2,00E-1
Certainty:
0,000
%
∞

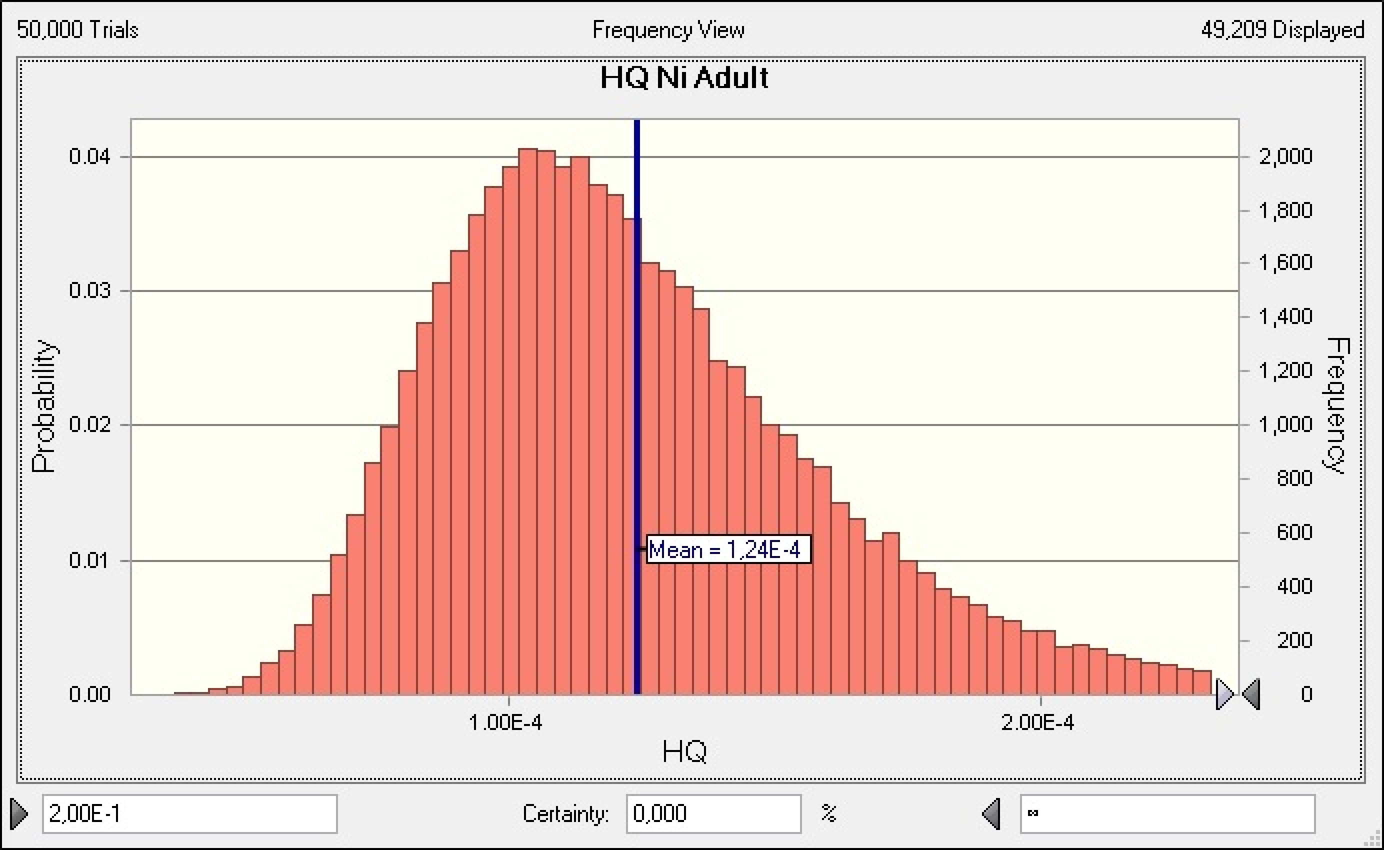
50,000 Trials
Frequency View
49,209 Displayed
HQ Ni Adult
Probability
Frequency
0.04
0.03
0.02
0.01
0.00
2,000
1,800
1,600
1,400
1,200
1,000
800
600
400
200
0
Mean = 1,24E-4
1.00E-4
2.00E-4
HQ
2,00E-1
Certainty:
0,000
%
∞

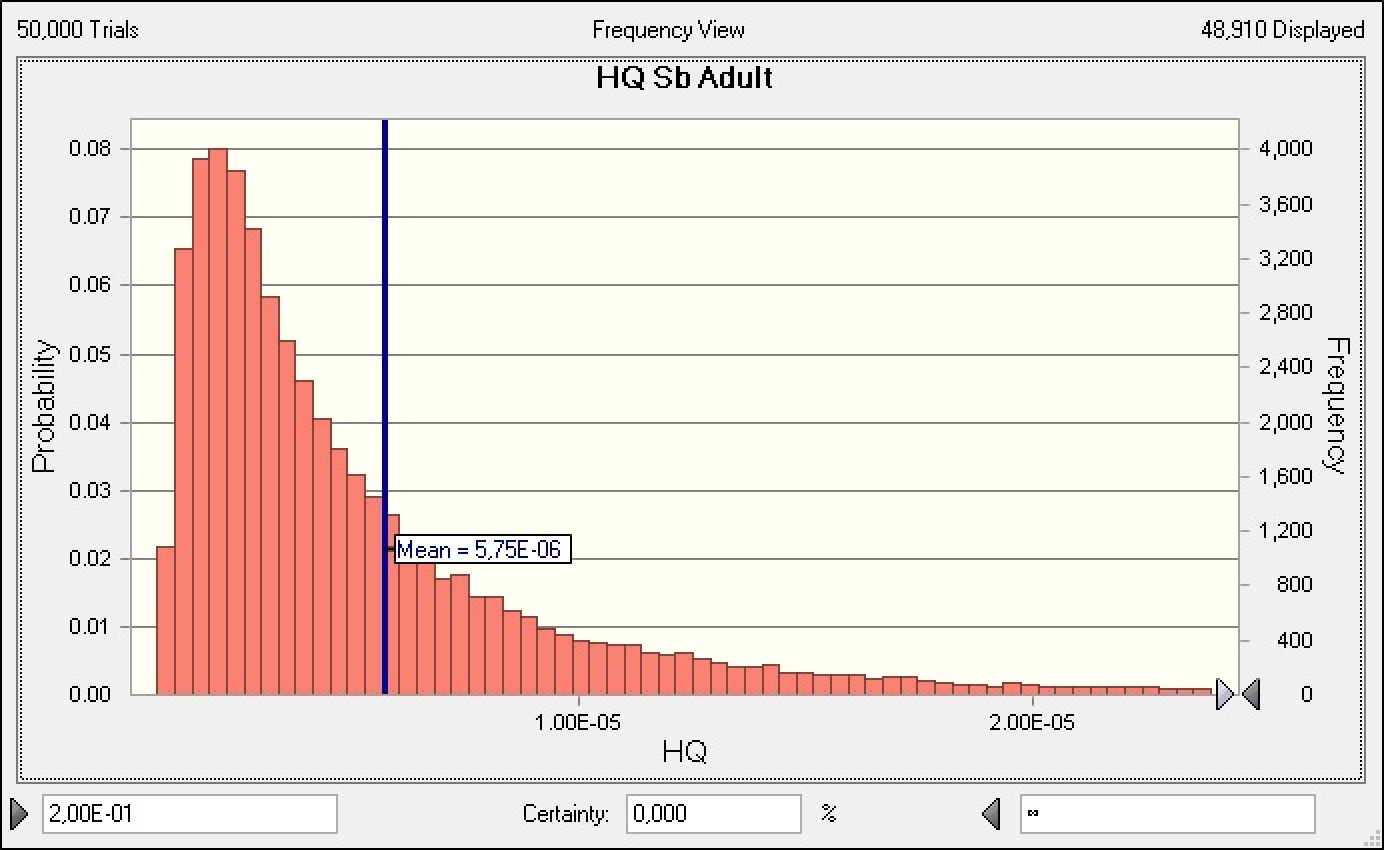

50,000 Trials
Frequency View
48,910 Displayed
HQ Sb Adult
Probability
0.08
0.07
0.06
0.05
0.04
0.03
0.02
0.01
0.00
Frequency
4,000
3,600
3,200
2,800
2,400
2,000
1,600
1,200
800
400
0
Mean = 5,75E-06
1.00E-05
2.00E-05
HQ
2,00E-01
Certainty:
0,000
%
∞

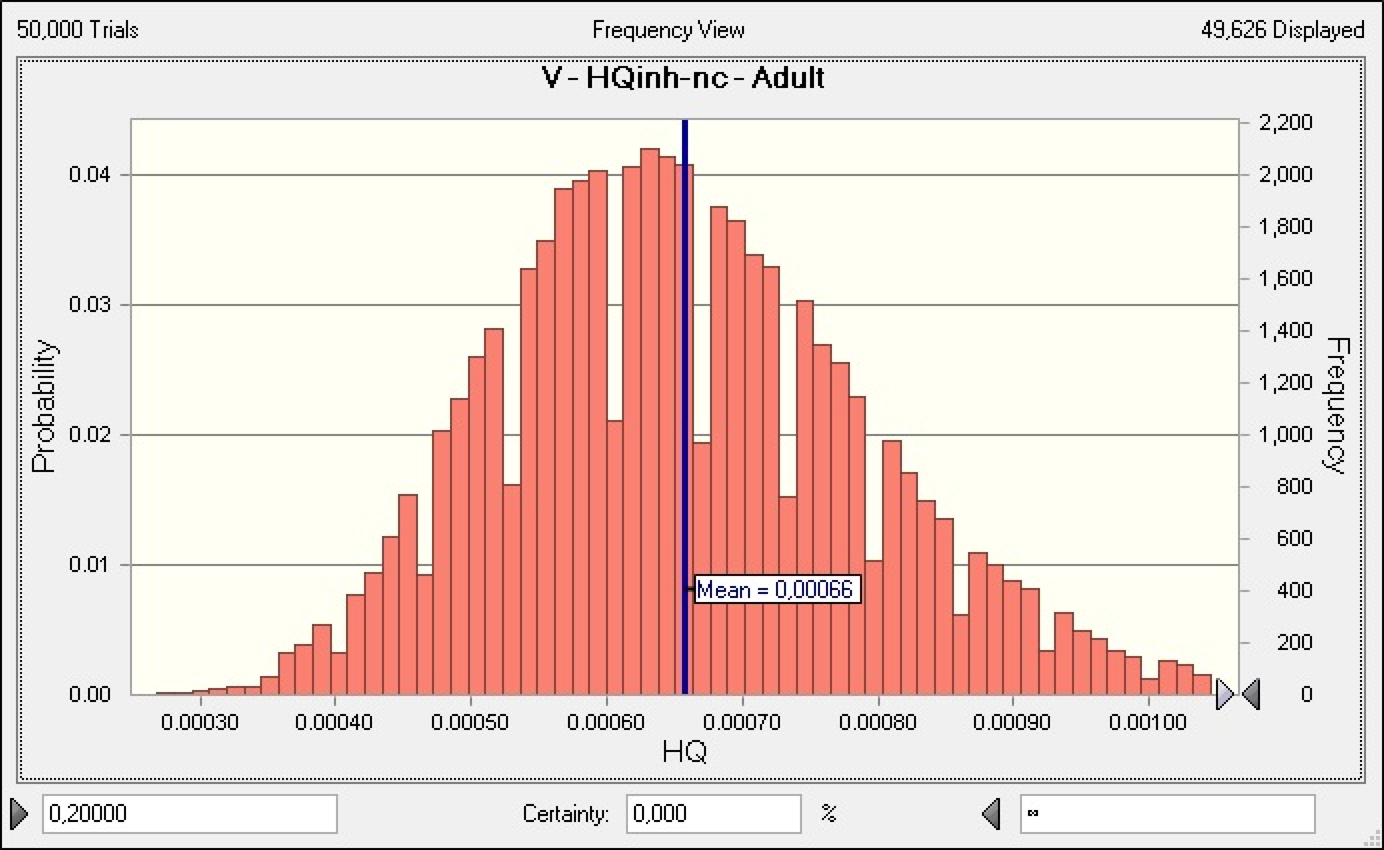

50,000 Trials
Frequency View
49,626 Displayed
V - HQinh-nc - Adult
Probability
Frequency
0.04
0.03
0.02
0.01
0.00
2,200
2,000
1,800
1,600
1,400
1,200
1,000
800
600
400
200
0
Mean = 0,00066
0.00030
0.00040
0.00050
0.00060
0.00070
0.00080
0.00090
0.00100
HQ
0,20000
Certainty:
0,000
%
∞

HIs INGESTION

CHILDREN

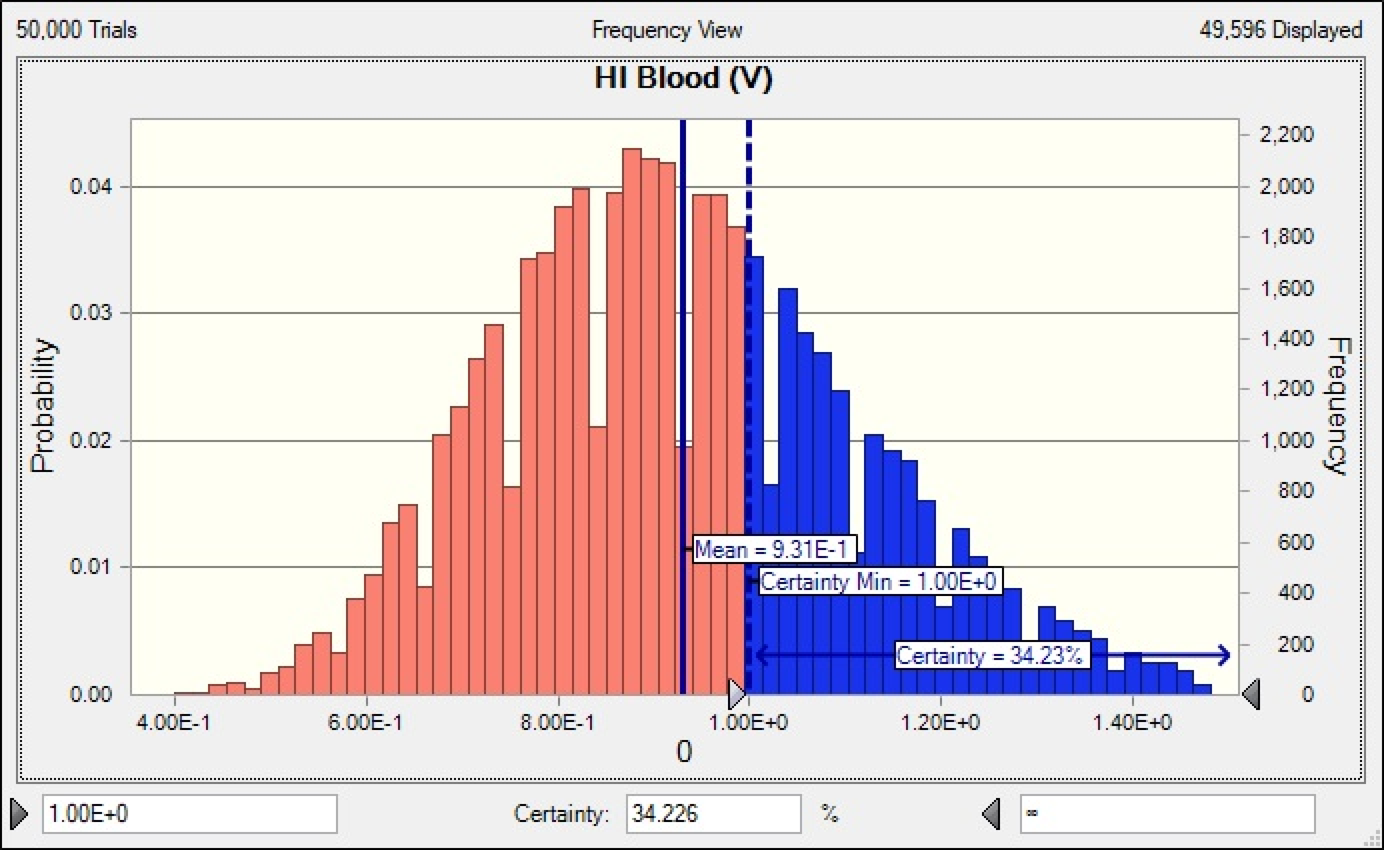

50,000 Trials
Frequency View
49,596 Displayed
HI Blood (V)
Probability
Frequency
0.04
0.03
0.02
0.01
0.00
2,200
2,000
1,800
1,600
1,400
1,200
1,000
800
600
400
200
0
Mean = 9.31E-1
Certainty Min = 1.00E+0
Certainty = 34.23%
4.00E-1
6.00E-1
8.00E-1
1.00E+0
1.20E+0
1.40E+0
0
1.00E+0
Certainty:
34.226
%
∞

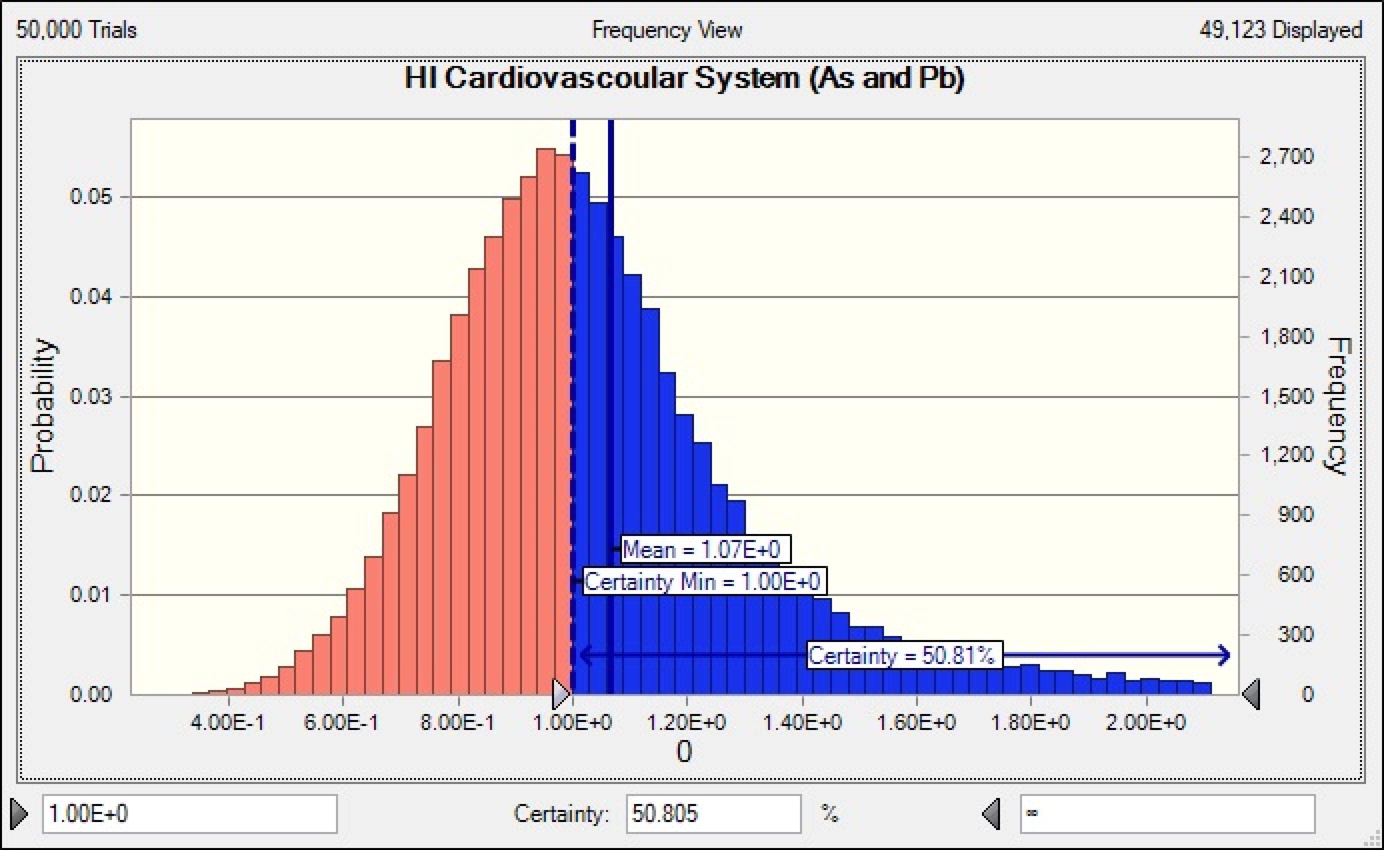
50,000 Trials
Frequency View
49,123 Displayed
HI Cardiovascoular System (As and Pb)
Probability
Frequency
0.05
0.04
0.03
0.02
0.01
0.00
2,700
2,400
2,100
1,800
1,500
1,200
900
600
300
0
Mean = 1.07E+0
Certainty Min = 1.00E+0
Certainty = 50.81%
4.00E-1
6.00E-1
8.00E-1
1.00E+0
1.20E+0
1.40E+0
1.60E+0
1.80E+0
2.00E+0
0
1.00E+0
Certainty:
50.805
%
∞

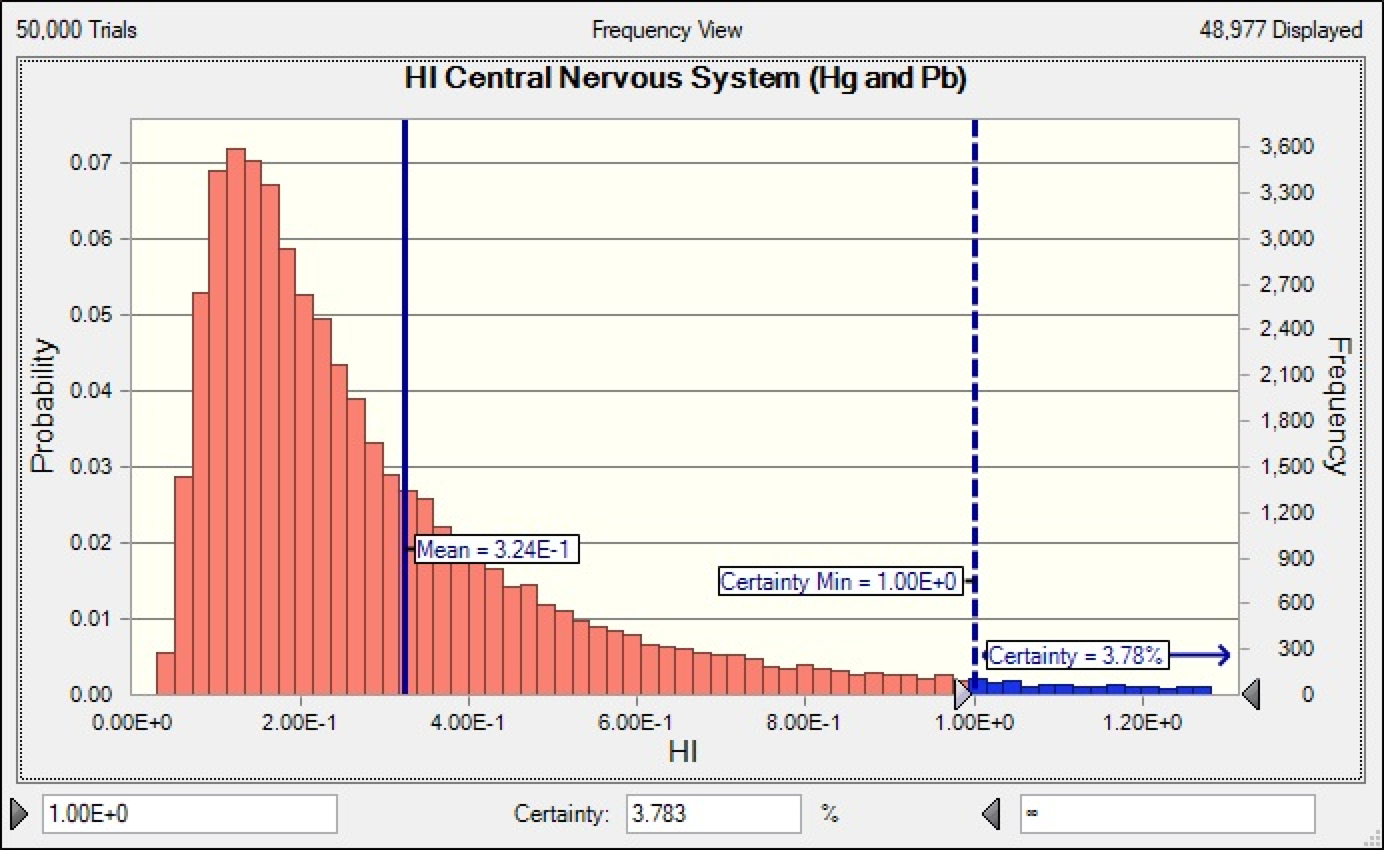
50,000 Trials
Frequency View
48,977 Displayed
HI Central Nervous System (Hg and Pb)
Probability
Frequency
0.07
0.06
0.05
0.04
0.03
0.02
0.01
0.00
3,600
3,300
3,000
2,700
2,400
2,100
1,800
1,500
1,200
900
600
300
0
Mean = 3.24E-1
Certainty Min = 1.00E+0
Certainty = 3.78%
0.00E+0
2.00E-1
4.00E-1
6.00E-1
8.00E-1
1.00E+0
1.20E+0
HI
1.00E+0
Certainty:
3.783
%
∞

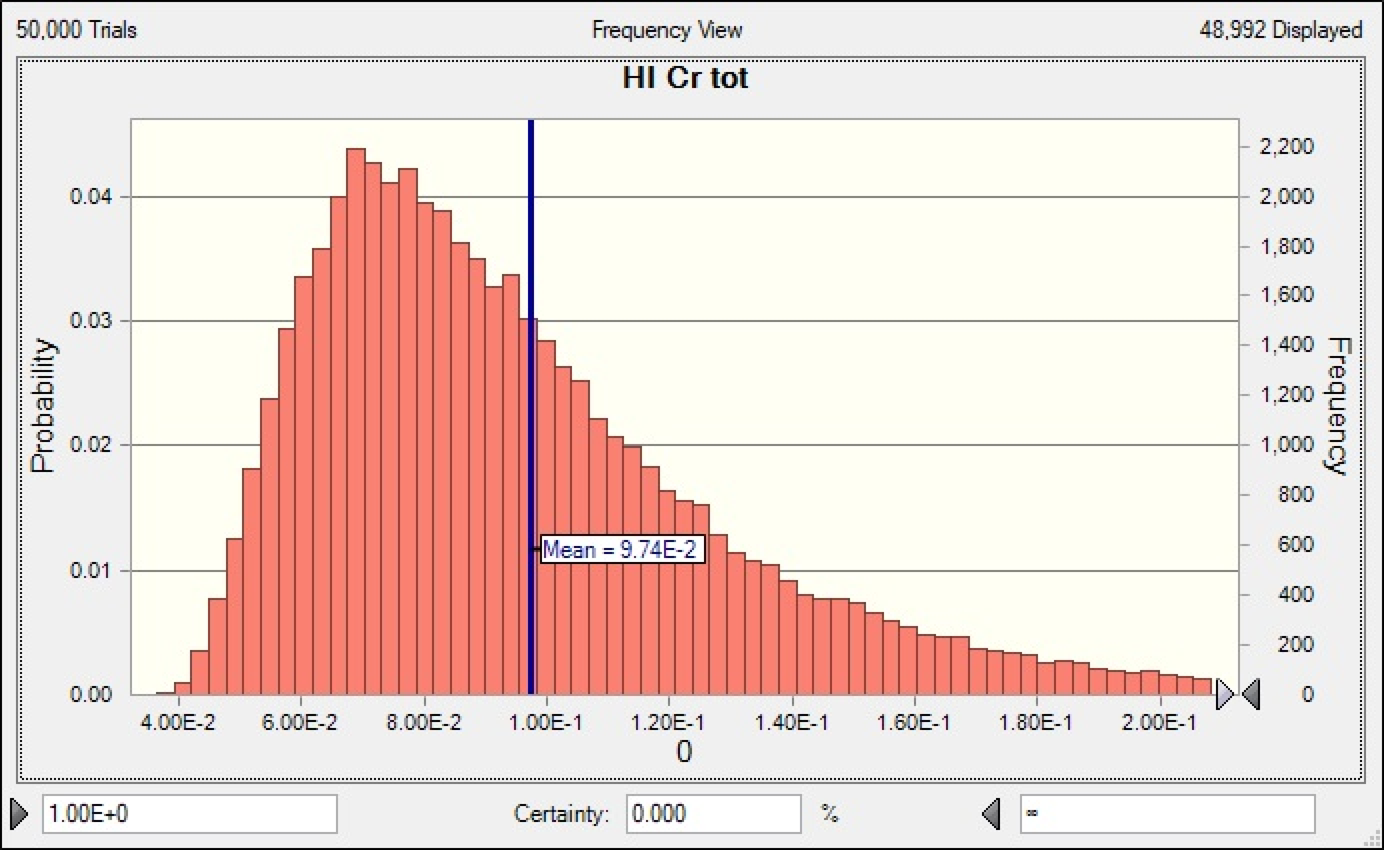
50,000 Trials
Frequency View
48,992 Displayed
HI Cr tot
Probability
Frequency
Mean = 9.74E-2
0.04
0.03
0.02
0.01
0.00
2,200
2,000
1,800
1,600
1,400
1,200
1,000
800
600
400
200
0
4.00E-2
6.00E-2
8.00E-2
1.00E-1
1.20E-1
1.40E-1
1.60E-1
1.80E-1
2.00E-1
0
1.00E+0
Certainty:
0.000
%
∞

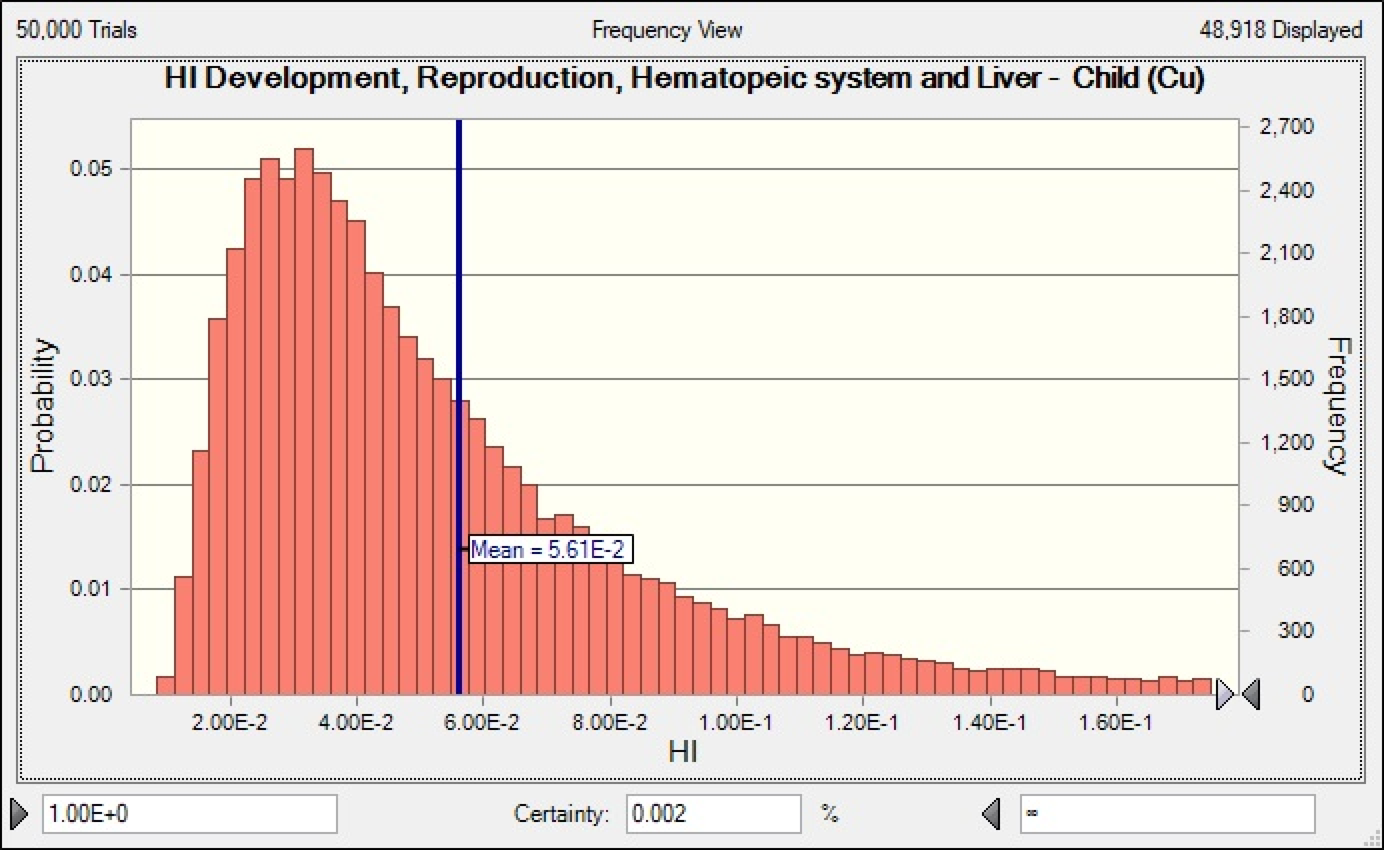

50,000 Trials
Frequency View
48,918 Displayed
HI Development, Reproduction, Hematopeic system and Liver - Child (Cu)
Probability
Frequency
0.05
0.04
0.03
0.02
0.01
0.00
2,700
2,400
2,100
1,800
1,500
1,200
900
600
300
0
Mean = 5.61E-2
2.00E-2
4.00E-2
6.00E-2
8.00E-2
1.00E-1
1.20E-1
1.40E-1
1.60E-1
HI
1.00E+0
Certainty: 0.002 %
∞

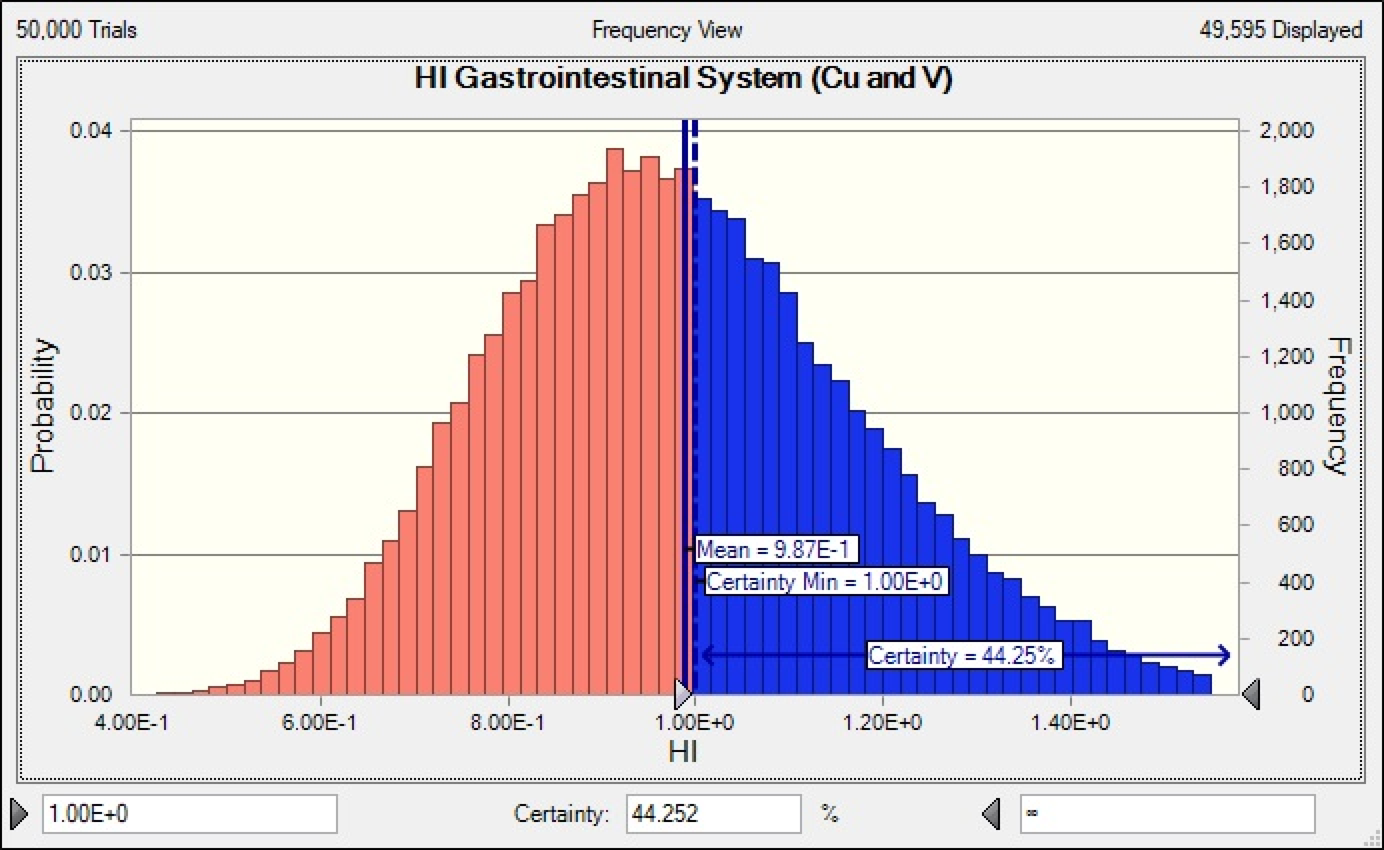

50,000 Trials
Frequency View
49,595 Displayed
HI Gastrointestinal System (Cu and V)
Probability
Frequency
0.04
0.03
0.02
0.01
0.00
2,000
1,800
1,600
1,400
1,200
1,000
800
600
400
200
0
Mean = 9.87E-1
Certainty Min = 1.00E+0
Certainty = 44.25%
4.00E-1
6.00E-1
8.00E-1
1.00E+0
1.20E+0
1.40E+0
HI
1.00E+0
Certainty:
44.252
%
∞

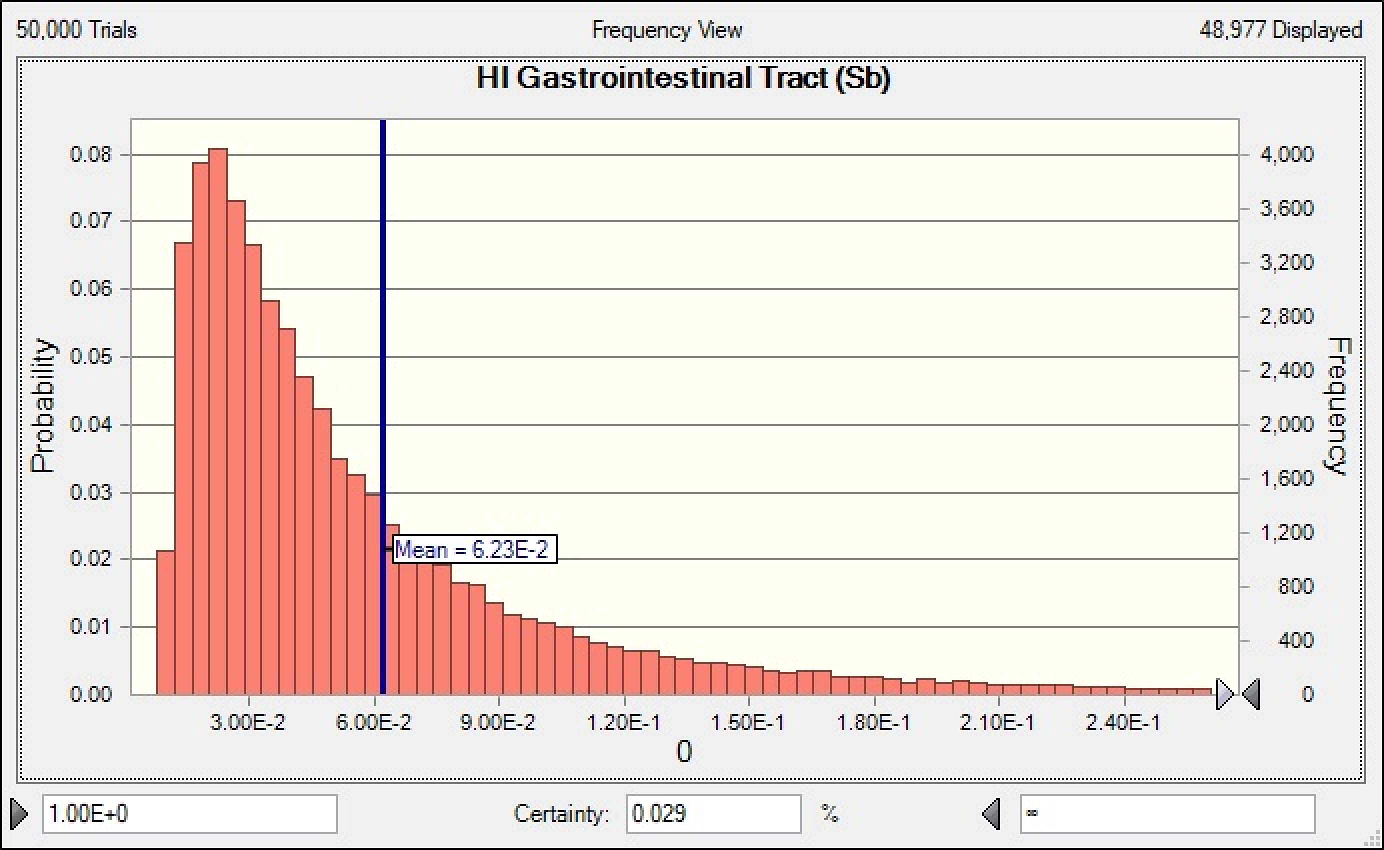

50,000 Trials
Frequency View
48,977 Displayed
HI Gastrointestinal Tract (Sb)
Probability
Frequency
0.08
0.07
0.06
0.05
0.04
0.03
0.02
0.01
0.00
4,000
3,600
3,200
2,800
2,400
2,000
1,600
1,200
800
400
0
Mean = 6.23E-2
3.00E-2
6.00E-2
9.00E-2
1.20E-1
1.50E-1
1.80E-1
2.10E-1
2.40E-1
0
1.00E+0
Certainty:
0.029
%
∞

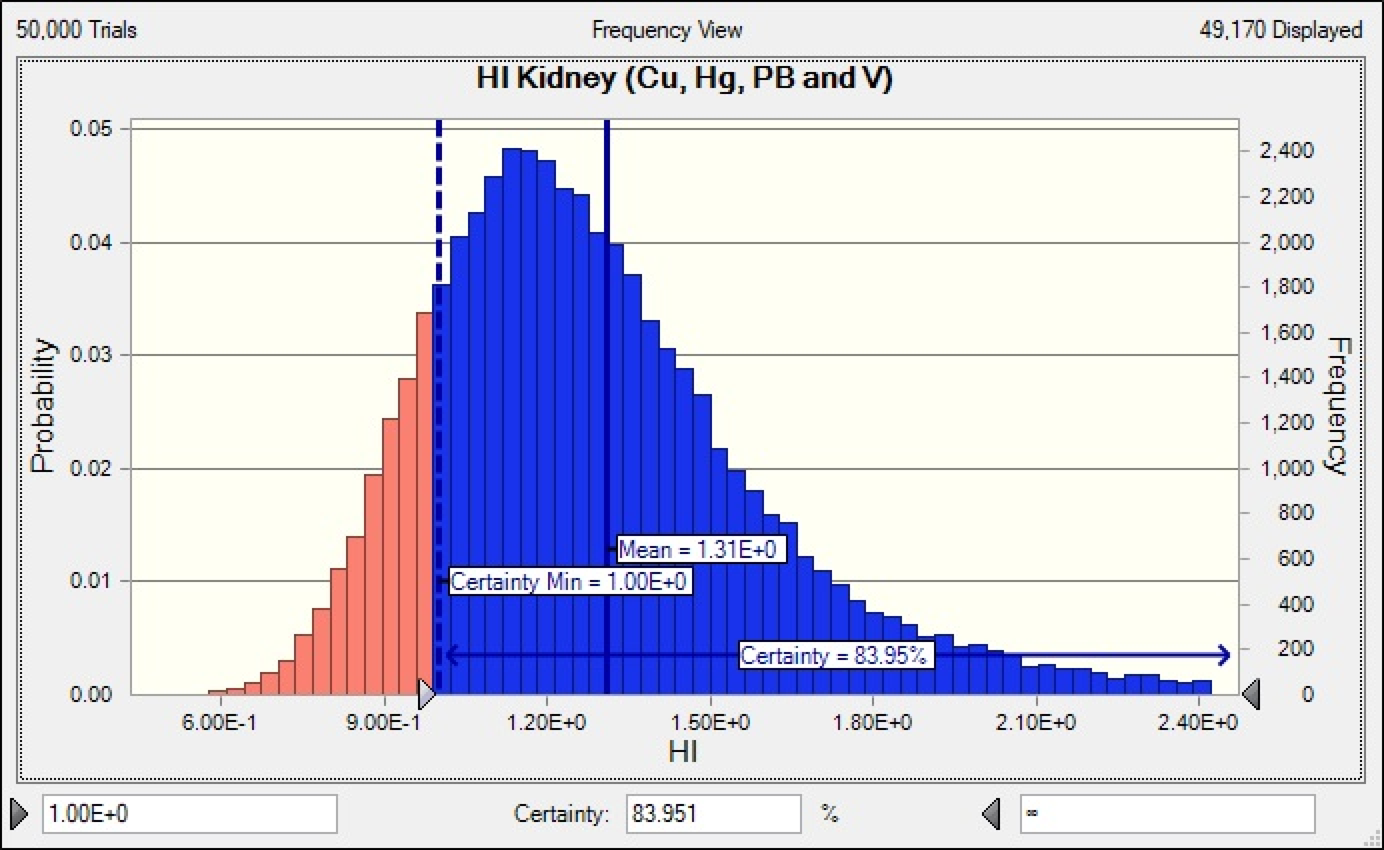

50,000 Trials
Frequency View
49,170 Displayed
HI Kidney (Cu, Hg, PB and V)
Probability
Frequency
0.05
0.04
0.03
0.02
0.01
0.00
2,400
2,200
2,000
1,800
1,600
1,400
1,200
1,000
800
600
400
200
0
Mean = 1.31E+0
Certainty Min = 1.00E+0
Certainty = 83.95%
6.00E-1
9.00E-1
1.20E+0
1.50E+0
1.80E+0
2.10E+0
2.40E+0
HI
1.00E+0
Certainty:
83.951
%
∞

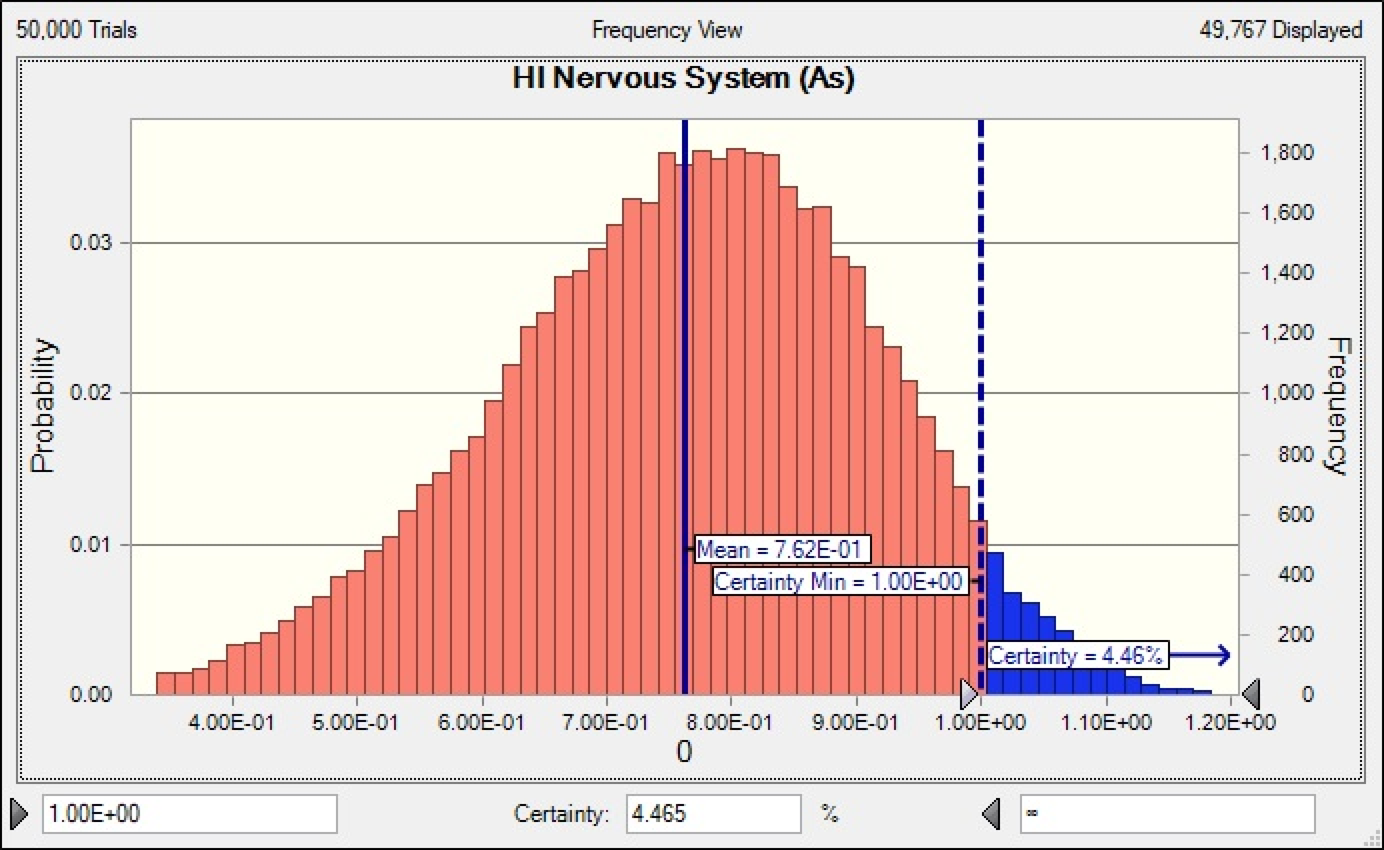

50,000 Trials
Frequency View
49,767 Displayed
HI Nervous System (As)
Probability
Frequency
0.03
0.02
0.01
0.00
1,800
1,600
1,400
1,200
1,000
800
600
400
200
0
Mean = 7.62E-01
Certainty Min = 1.00E+00
Certainty = 4.46%
4.00E-01
5.00E-01
6.00E-01
7.00E-01
8.00E-01
9.00E-01
1.00E+00
1.10E+00
1.20E+00
0
1.00E+00
Certainty:
4.465
%
∞

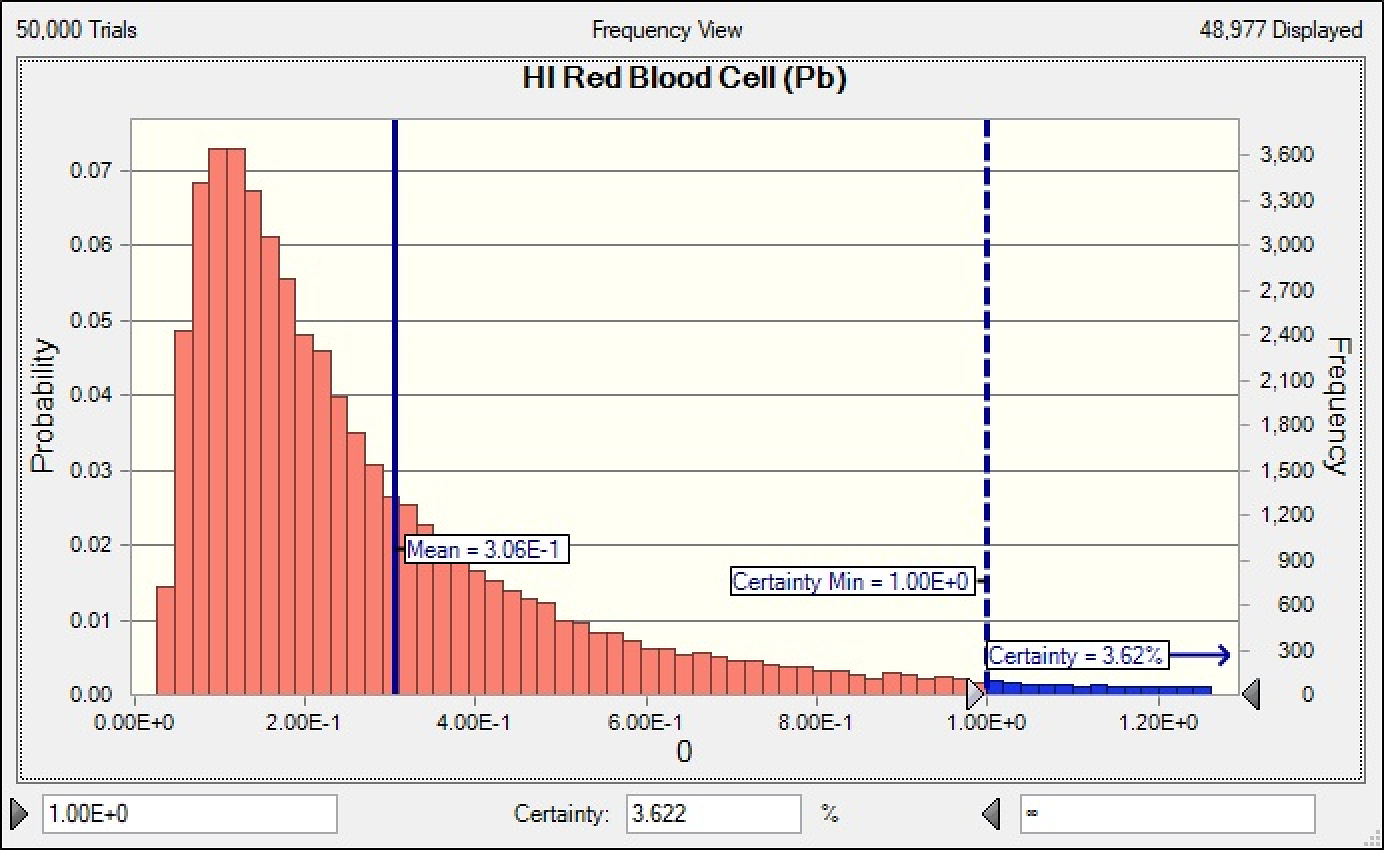

50,000 Trials
Frequency View
48,977 Displayed
HI Red Blood Cell (Pb)
Probability
Frequency
0.00
0.01
0.02
0.03
0.04
0.05
0.06
0.07
0
300
600
900
1,200
1,500
1,800
2,100
2,400
2,700
3,000
3,300
3,600
0.00E+0
2.00E-1
4.00E-1
6.00E-1
8.00E-1
1.00E+0
1.20E+0
0
Mean = 3.06E-1
Certainty Min = 1.00E+0
Certainty = 3.62%
1.00E+0
Certainty:
3.622
%
∞

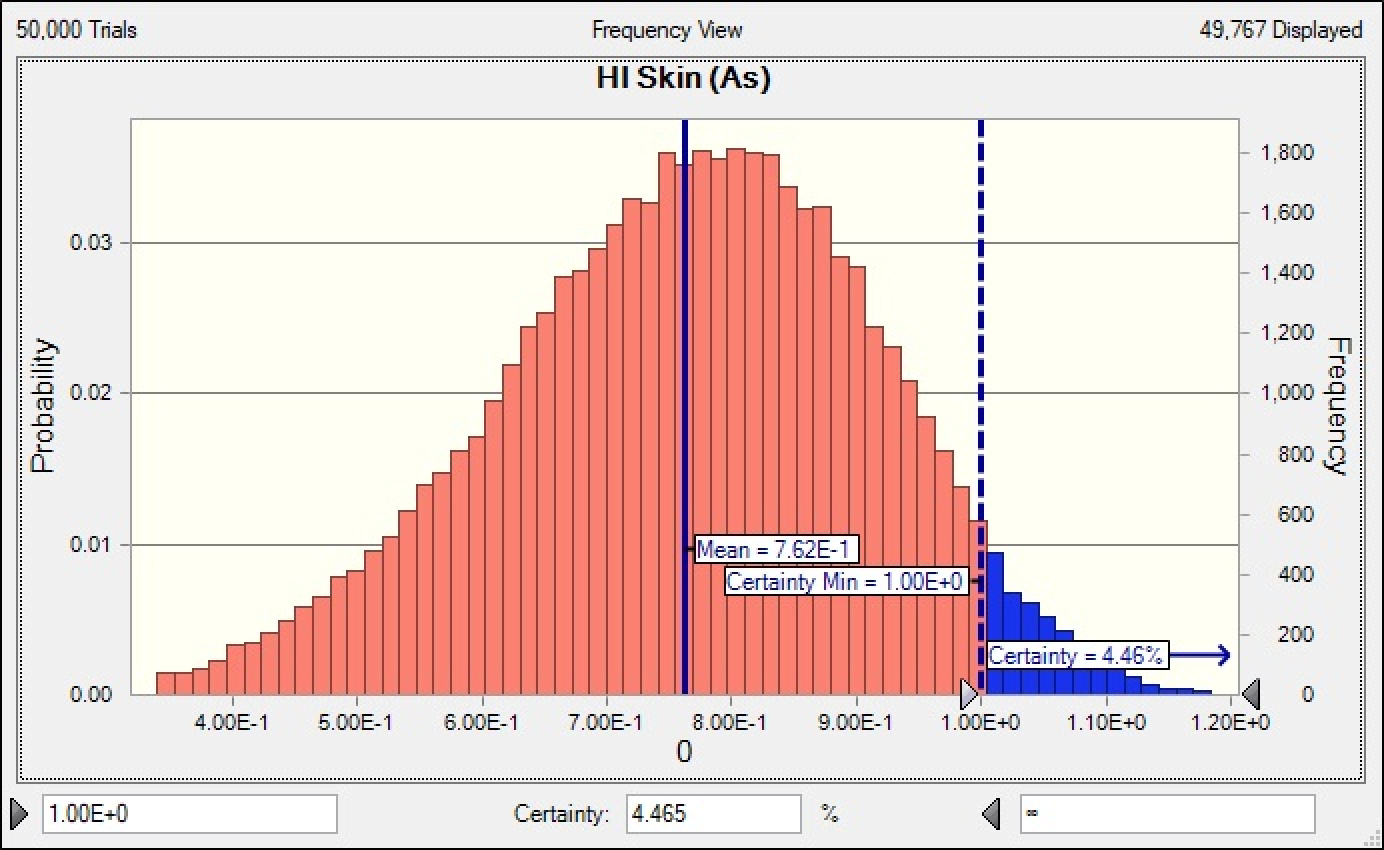
50,000 Trials
Frequency View
49,767 Displayed
HI Skin (As)
Probability
Frequency
0.00
0.01
0.02
0.03
0
200
400
600
800
1,000
1,200
1,400
1,600
1,800
4.00E-1
5.00E-1
6.00E-1
7.00E-1
8.00E-1
9.00E-1
1.00E+0
1.10E+0
1.20E+0
0
Mean = 7.62E-1
Certainty Min = 1.00E+0
Certainty = 4.46%
1.00E+0
Certainty:
4.465
%
∞

ILCRs INHALATION CHILDREN

ILCR inhalation - Child

Element	**As**	**Be**	**Cd**	**Co**	**Cr_{Tot}**	**Ni**	**Pb**
Level of certainty	**0.00%**	**0.00%**	**0.00%**	**0.00%**	**0.00%**	**0.00%**	**9.27%**
Mean	2.80E-10	9.83E-12	1.39E-11	8.15E-11	1.14E-10	3.23E-09	4.70E-07
Median	2.83E-10	9.84E-12	1.13E-11	8.14E-11	1.01E-10	3.07E-09	3.17E-07
Min	5.29E-11	-7.38E+12	2.17E-12	-1.40E-11	4.41E-11	8.79E-10	4.20E-08
Max	4.47E-10	2.63E-11	3.70E-10	1.92E-10	7.09E-10	1.07E-08	1.67E-05
St. Dev.	5.52E-11	3.79E-12	9.64E-12	1.88E-11	5.13E-11	1.00E-09	5.09E-07

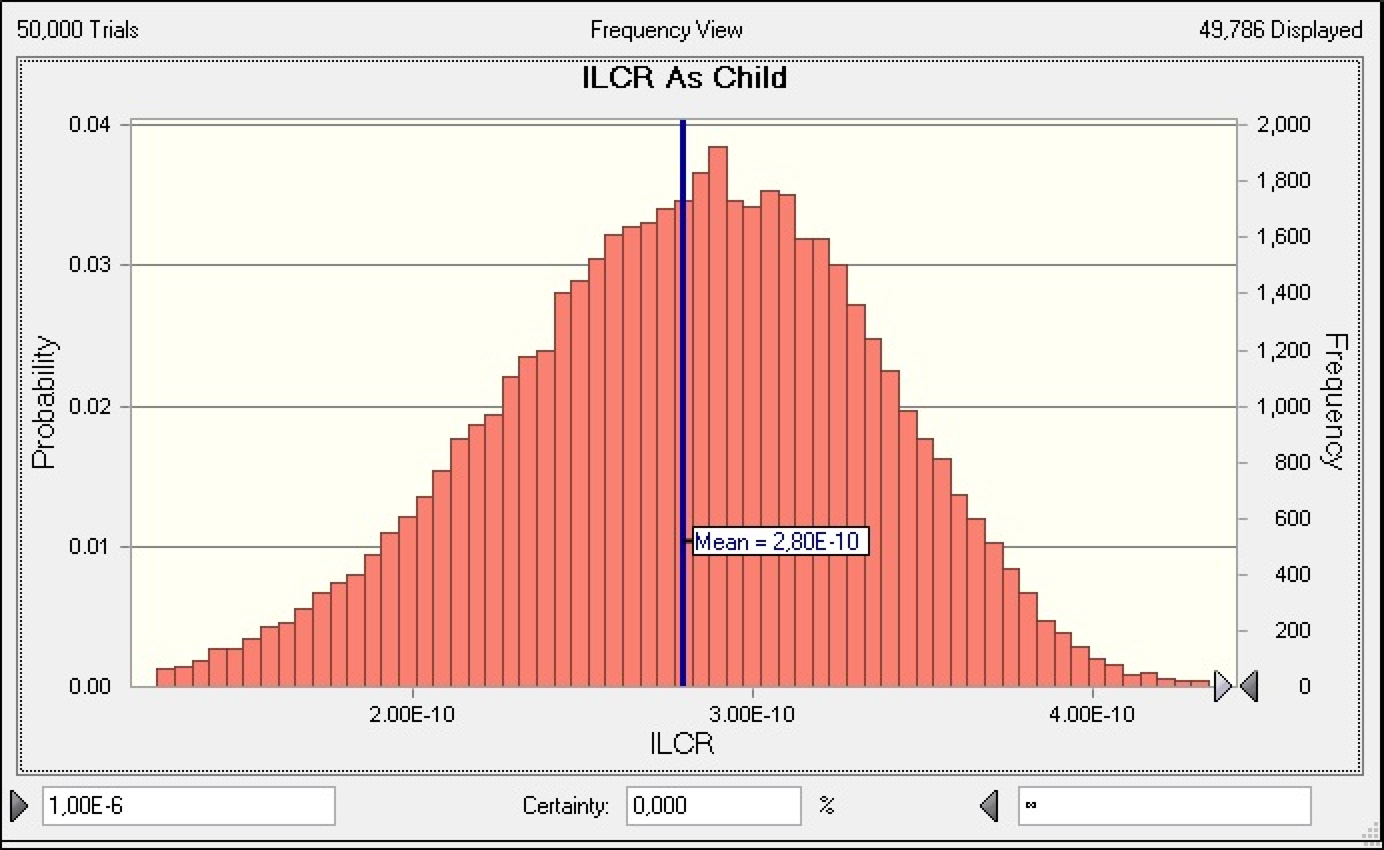
50,000 Trials
Frequency View
49,786 Displayed
ILCR As Child
Probability
Frequency
0.04
0.03
0.02
0.01
0.00
2,000
1,800
1,600
1,400
1,200
1,000
800
600
400
200
0
Mean = 2,80E-10
2.00E-10
3.00E-10
4.00E-10
ILCR
1,00E-6
Certainty:
0,000
%
∞

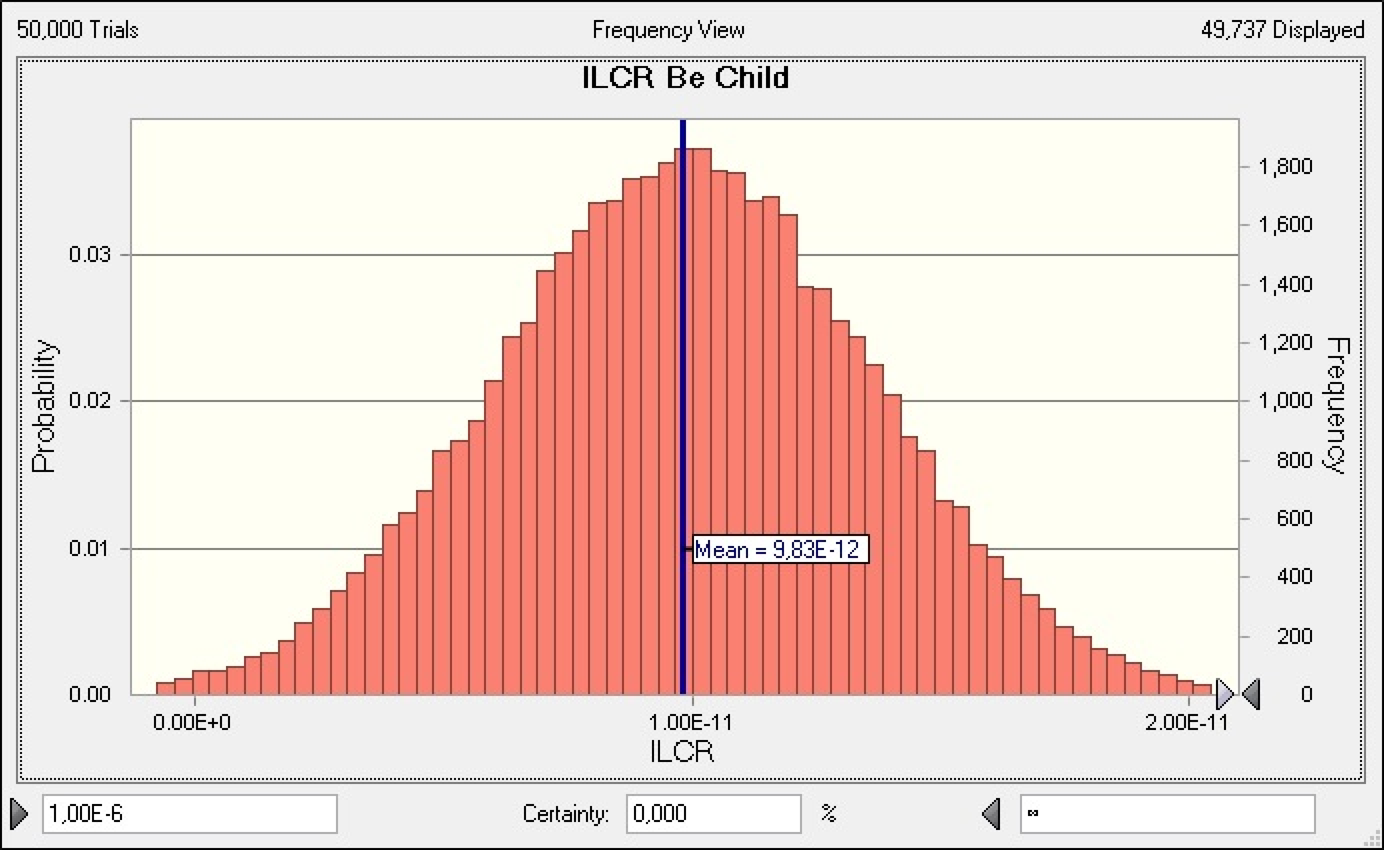

50,000 Trials
Frequency View
49,737 Displayed
ILCR Be Child
Probability
0.03
0.02
0.01
0.00
Frequency
1,800
1,600
1,400
1,200
1,000
800
600
400
200
0
Mean = 9,83E-12
0.00E+0
1.00E-11
2.00E-11
ILCR
1,00E-6
Certainty:
0,000
%
∞

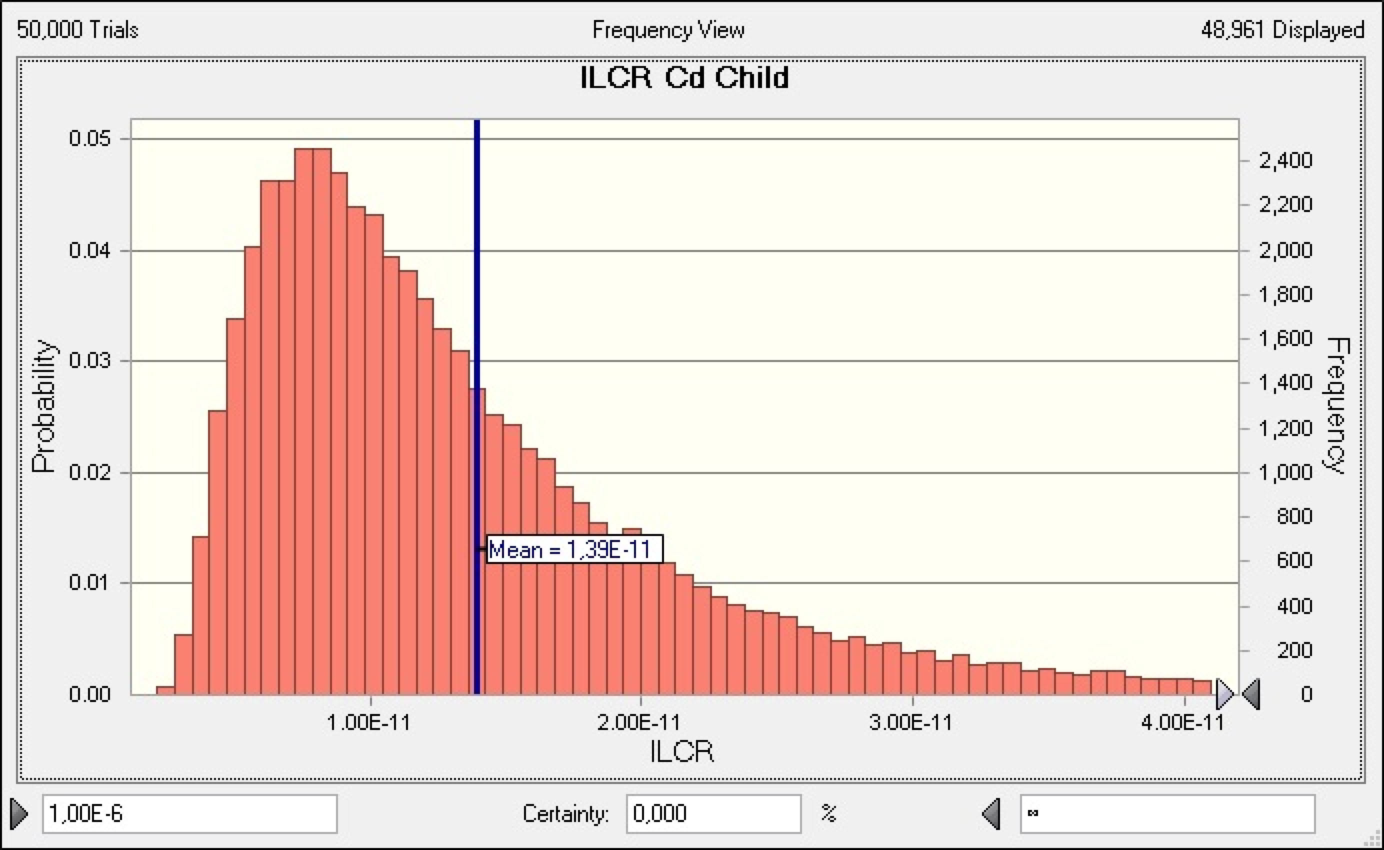
50,000 Trials
Frequency View
48,961 Displayed
ILCR Cd Child
Probability
Frequency
0.05
0.04
0.03
0.02
0.01
0.00
2,400
2,200
2,000
1,800
1,600
1,400
1,200
1,000
800
600
400
200
0
Mean = 1,39E-11
1.00E-11
2.00E-11
3.00E-11
4.00E-11
ILCR
1,00E-6
Certainty:
0,000
%
∞

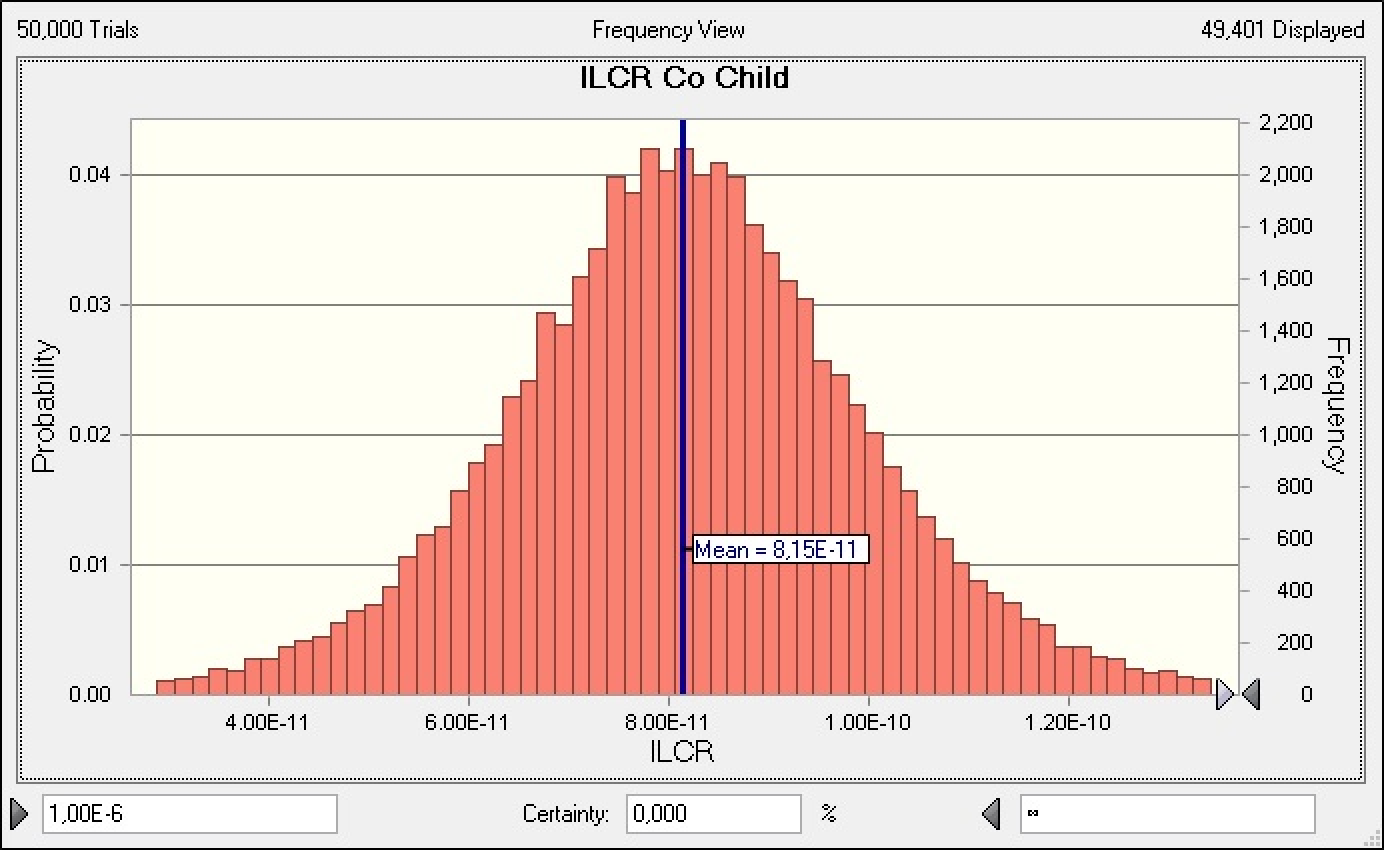

50,000 Trials
Frequency View
49,401 Displayed
ILCR Co Child
Probability
Frequency
0.00
0.01
0.02
0.03
0.04
0
200
400
600
800
1,000
1,200
1,400
1,600
1,800
2,000
2,200
Mean = 8,15E-11
4.00E-11
6.00E-11
8.00E-11
1.00E-10
1.20E-10
ILCR
1,00E-6
Certainty:
0,000
%
∞

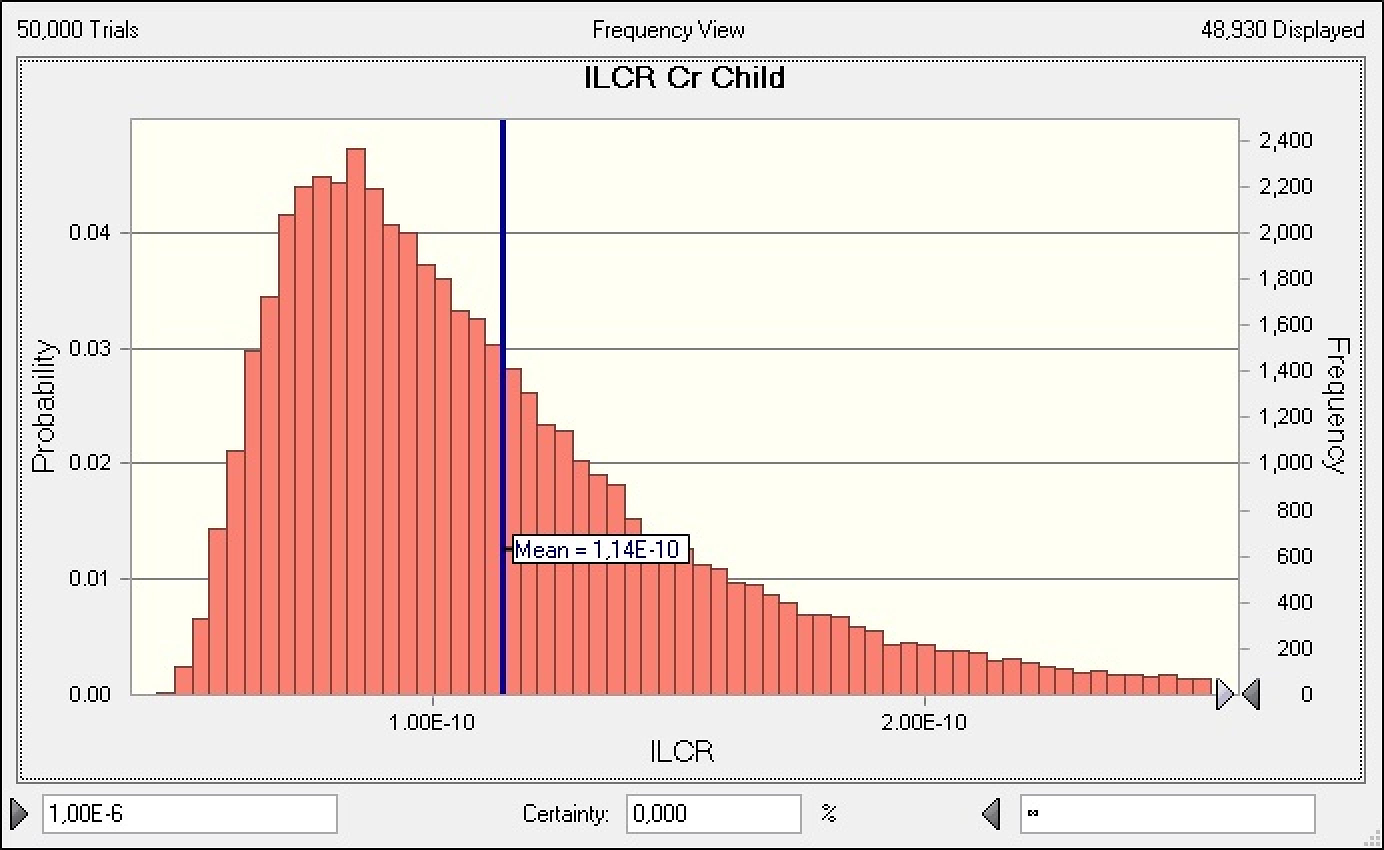

50,000 Trials
Frequency View
48,930 Displayed
ILCR Cr Child
Probability
Frequency
0.00
0.01
0.02
0.03
0.04
0
200
400
600
800
1,000
1,200
1,400
1,600
1,800
2,000
2,200
2,400
Mean = 1,14E-10
1.00E-10
2.00E-10
ILCR
1,00E-6
Certainty:
0,000
%
∞

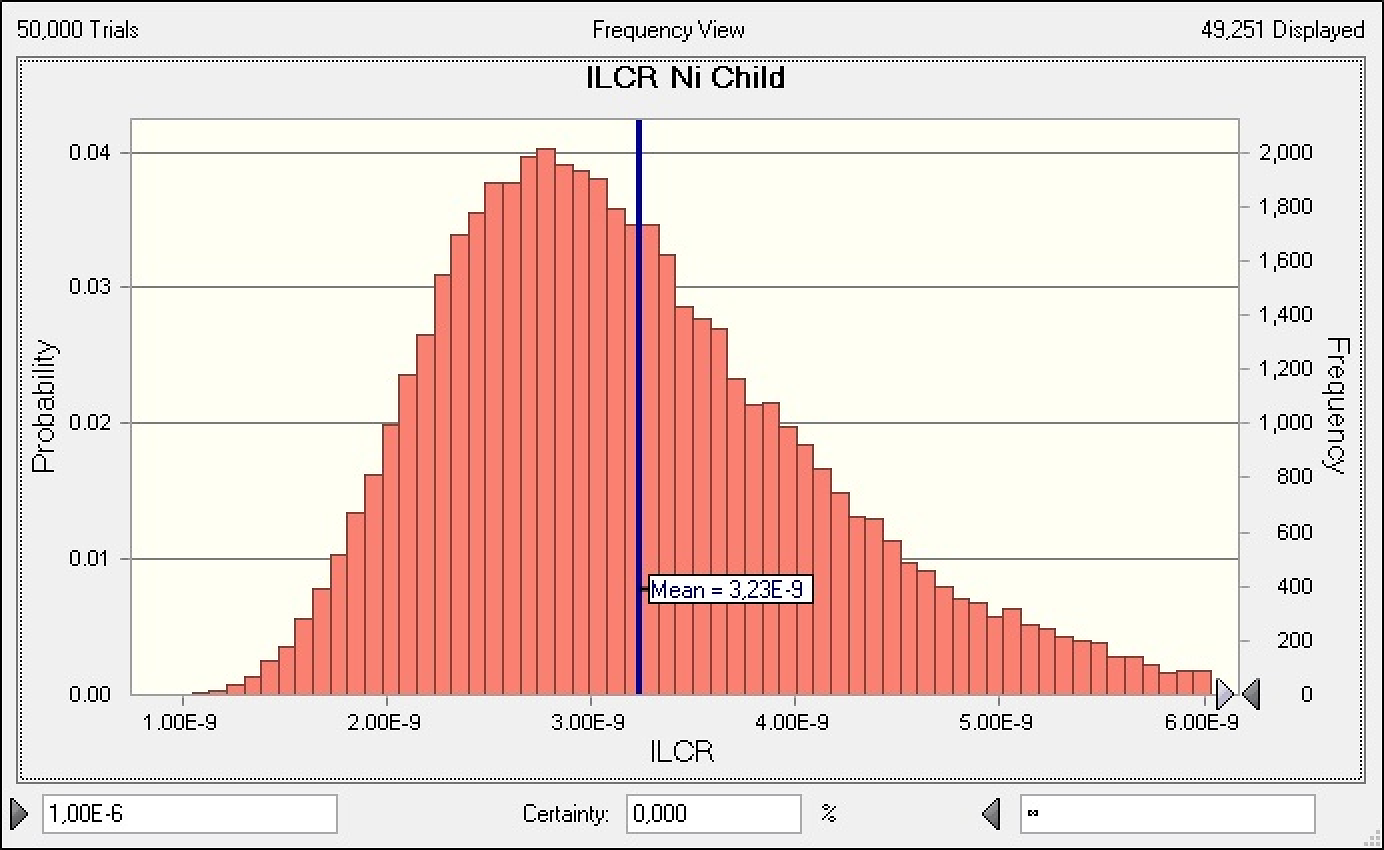
50,000 Trials
Frequency View
49,251 Displayed
ILCR Ni Child
Probability
0.04
0.03
0.02
0.01
0.00
Frequency
2,000
1,800
1,600
1,400
1,200
1,000
800
600
400
200
0
Mean = 3,23E-9
1.00E-9
2.00E-9
3.00E-9
4.00E-9
5.00E-9
6.00E-9
ILCR
1,00E-6
Certainty:
0,000
%
∞

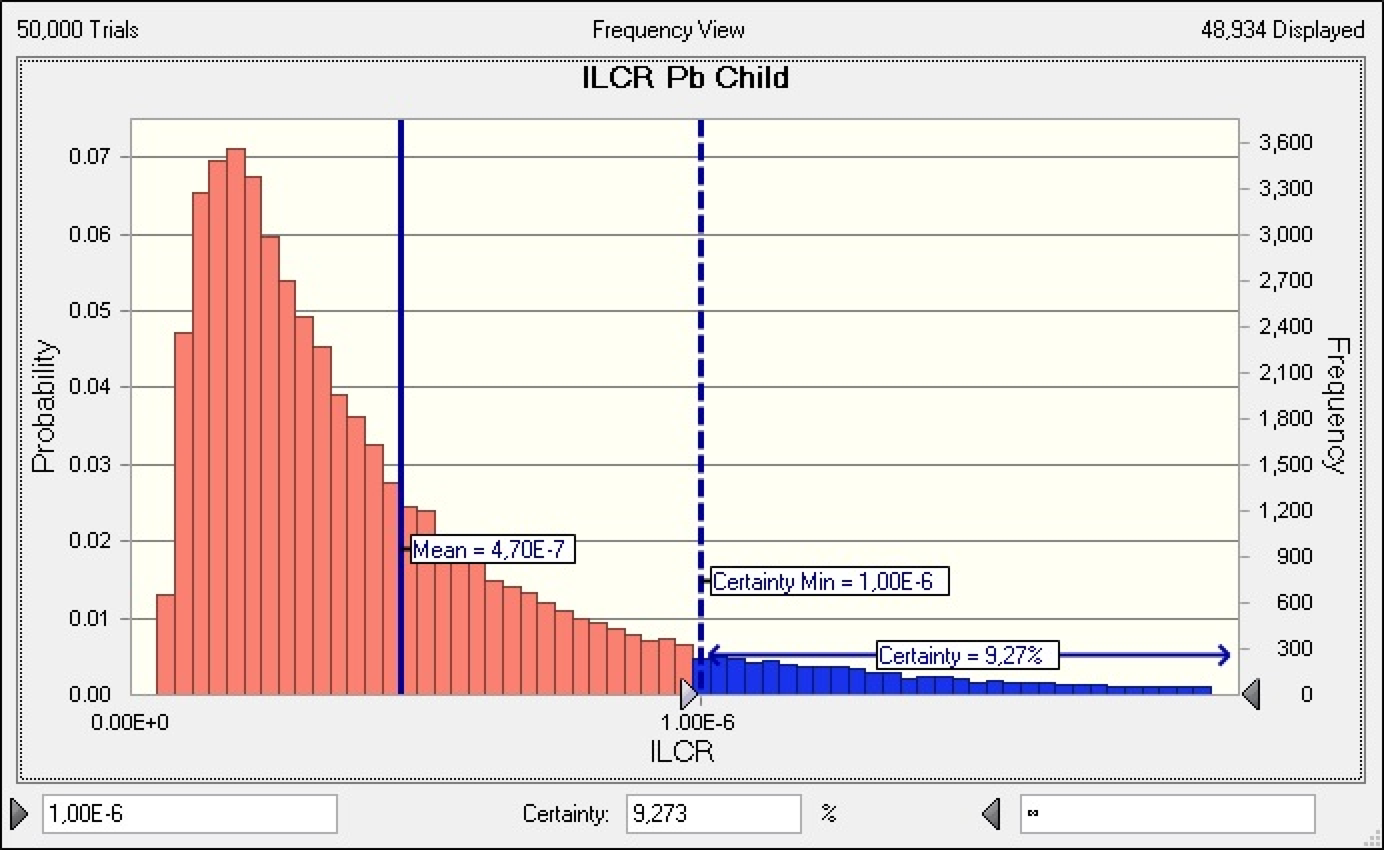
50,000 Trials
Frequency View
48,934 Displayed
ILCR Pb Child
Probability
0.07
0.06
0.05
0.04
0.03
0.02
0.01
0.00
Frequency
3,600
3,300
3,000
2,700
2,400
2,100
1,800
1,500
1,200
900
600
300
0
Mean = 4,70E-7
Certainty Min = 1,00E-6
Certainty = 9,27%
0.00E+0
1.00E-6
ILCR
1,00E-6
Certainty:
9,273
%
∞

ILCRs INHALATION
ADULTS

ILCR inhalation - Adult

Element	**As**	**Be**	**Cd**	**Co**	**Cr_{Tot}**	**Ni**	**Pb**
Level of certainty	**0.00%**	**0.00%**	**0.00%**	**0.00%**	**0.00%**	**0.00%**	**72.03%**
Mean	1.40E-09	4.91E-11	6.99E-11	4.07E-10	5.72E-10	1.61E-08	2.36E-06
Median	1.42E-09	4.91E-11	5.67-11	4.08E-10	5.06E-10	1.53E-08	1.59E-06
Min	3.43E-10	-3.31E-11	1.13E-11	-1.45E-10	2.23E-10	3.89E-09	2.07E-07
Max	2.36E-09	1.30E-10	1.28E-09	9.81E-10	4.02E-09	6.41E-08	6.64E-05
St. Dev.	2.75E-10	1.88E-11	4.48E-11	9.38E-11	2.57E-10	5.00E-09	2.57E-06

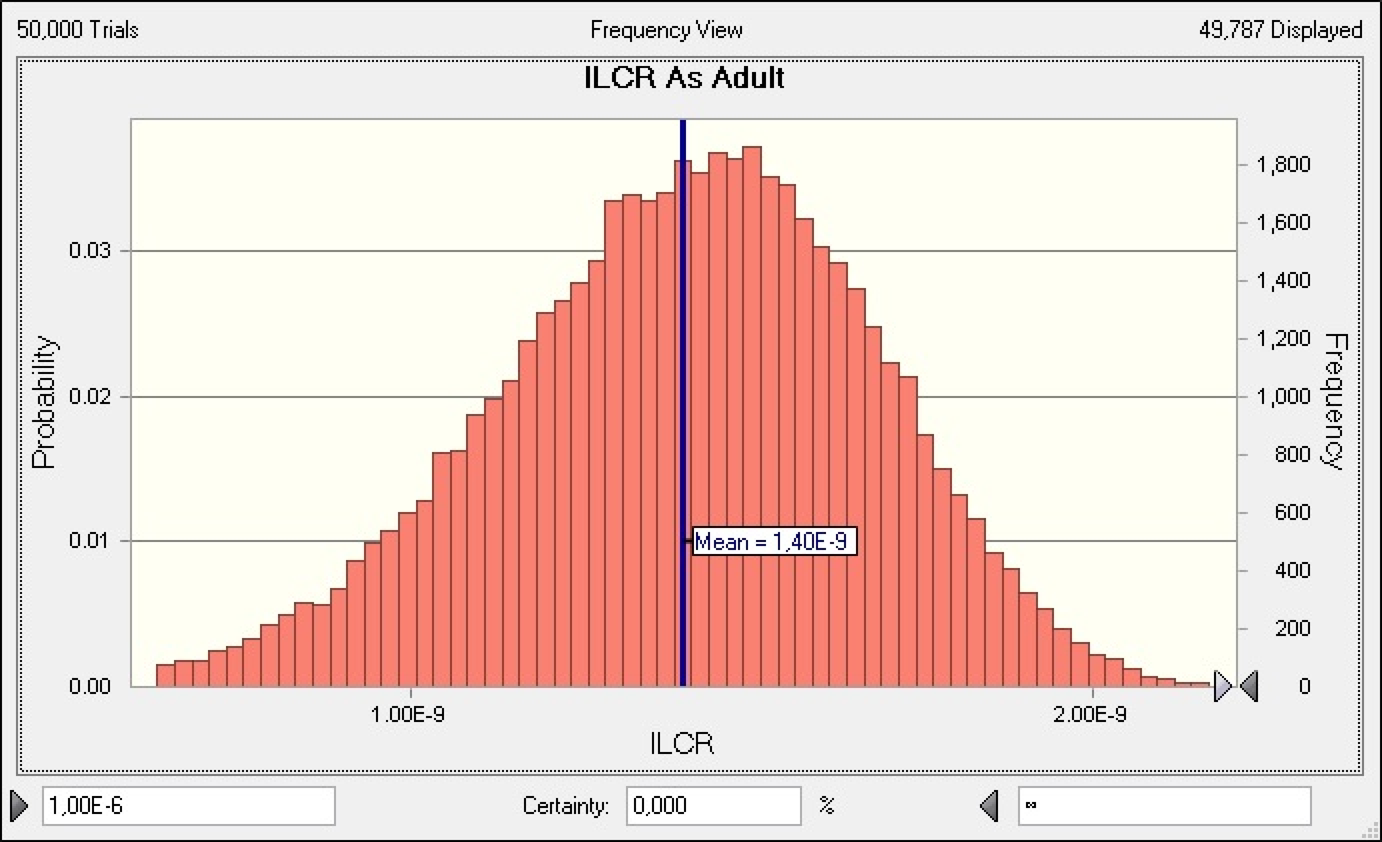

50,000 Trials
Frequency View
49,787 Displayed
ILCR As Adult
Probability
0.03
0.02
0.01
0.00
Frequency
1,800
1,600
1,400
1,200
1,000
800
600
400
200
0
Mean = 1,40E-9
1.00E-9
2.00E-9
ILCR
1,00E-6
Certainty:
0,000
%
∞

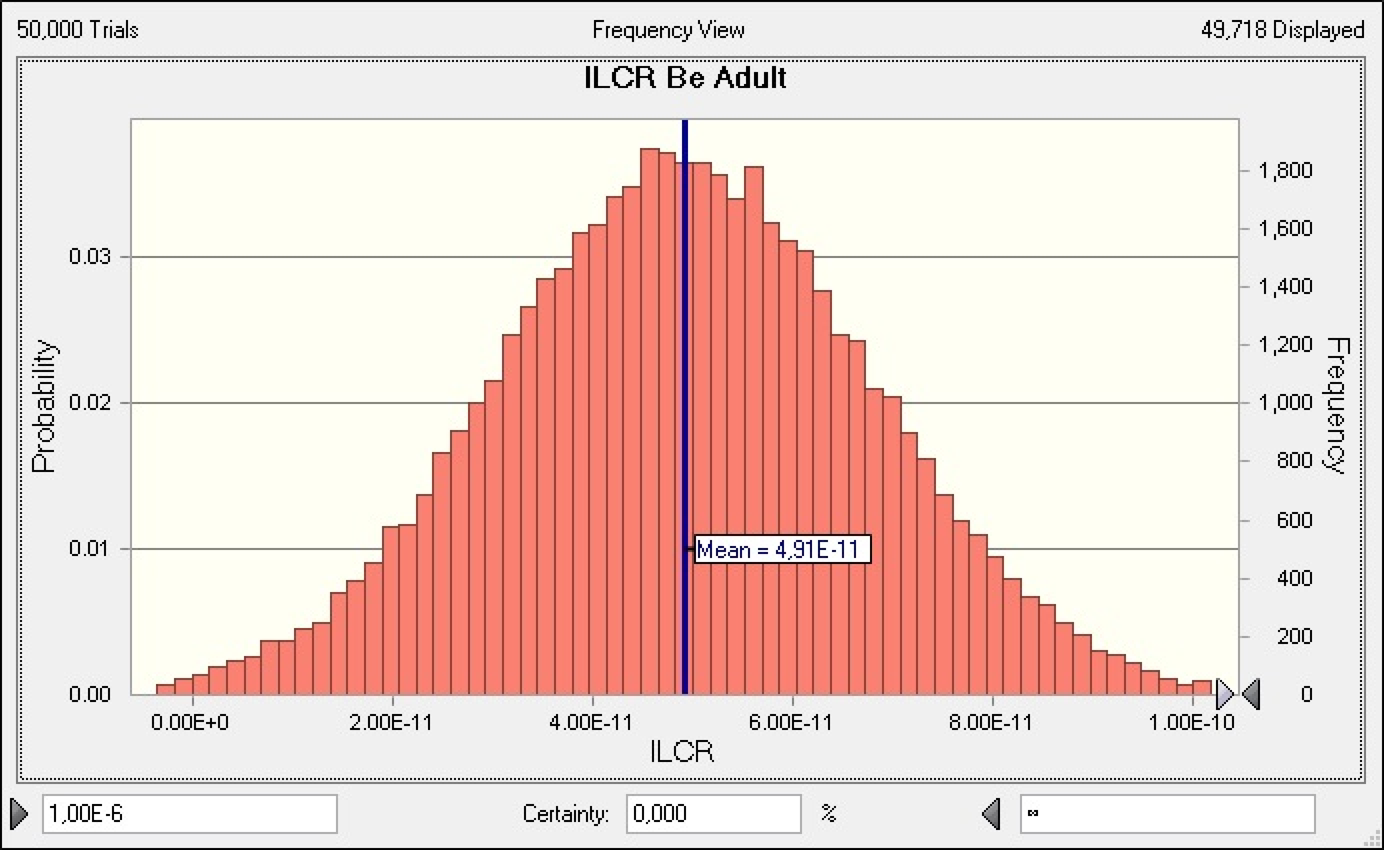
50,000 Trials
Frequency View
49,718 Displayed
ILCR Be Adult
Probability
0.03
0.02
0.01
0.00
Frequency
1,800
1,600
1,400
1,200
1,000
800
600
400
200
0
Mean = 4,91E-11
0.00E+0
2.00E-11
4.00E-11
6.00E-11
8.00E-11
1.00E-10
ILCR
1,00E-6
Certainty:
0,000
%
∞

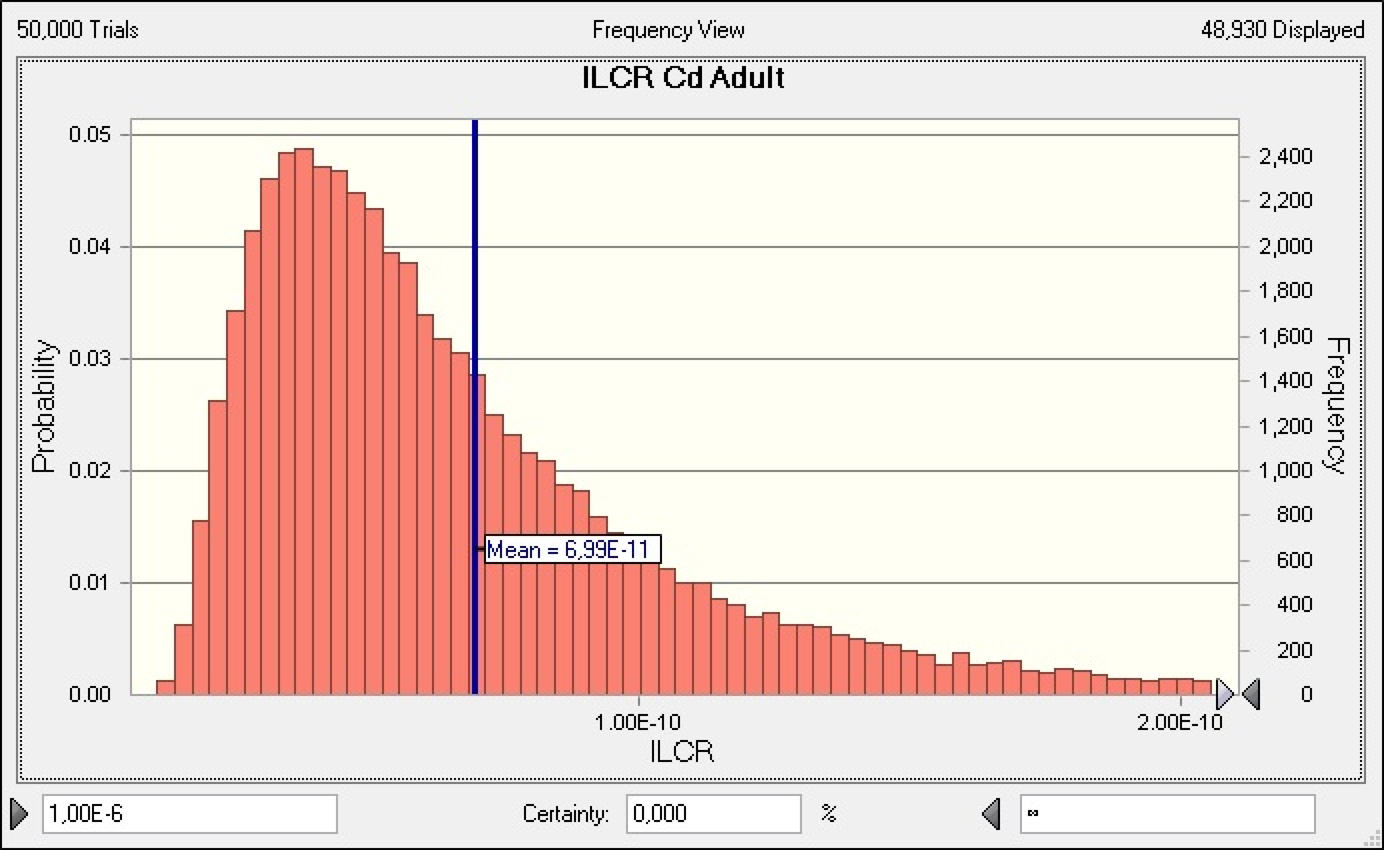
50,000 Trials
Frequency View
48,930 Displayed
ILCR Cd Adult
Probability
0.05
0.04
0.03
0.02
0.01
0.00
Frequency
2,400
2,200
2,000
1,800
1,600
1,400
1,200
1,000
800
600
400
200
0
Mean = 6,99E-11
1.00E-10
2.00E-10
ILCR
1,00E-6
Certainty:
0,000
%
∞

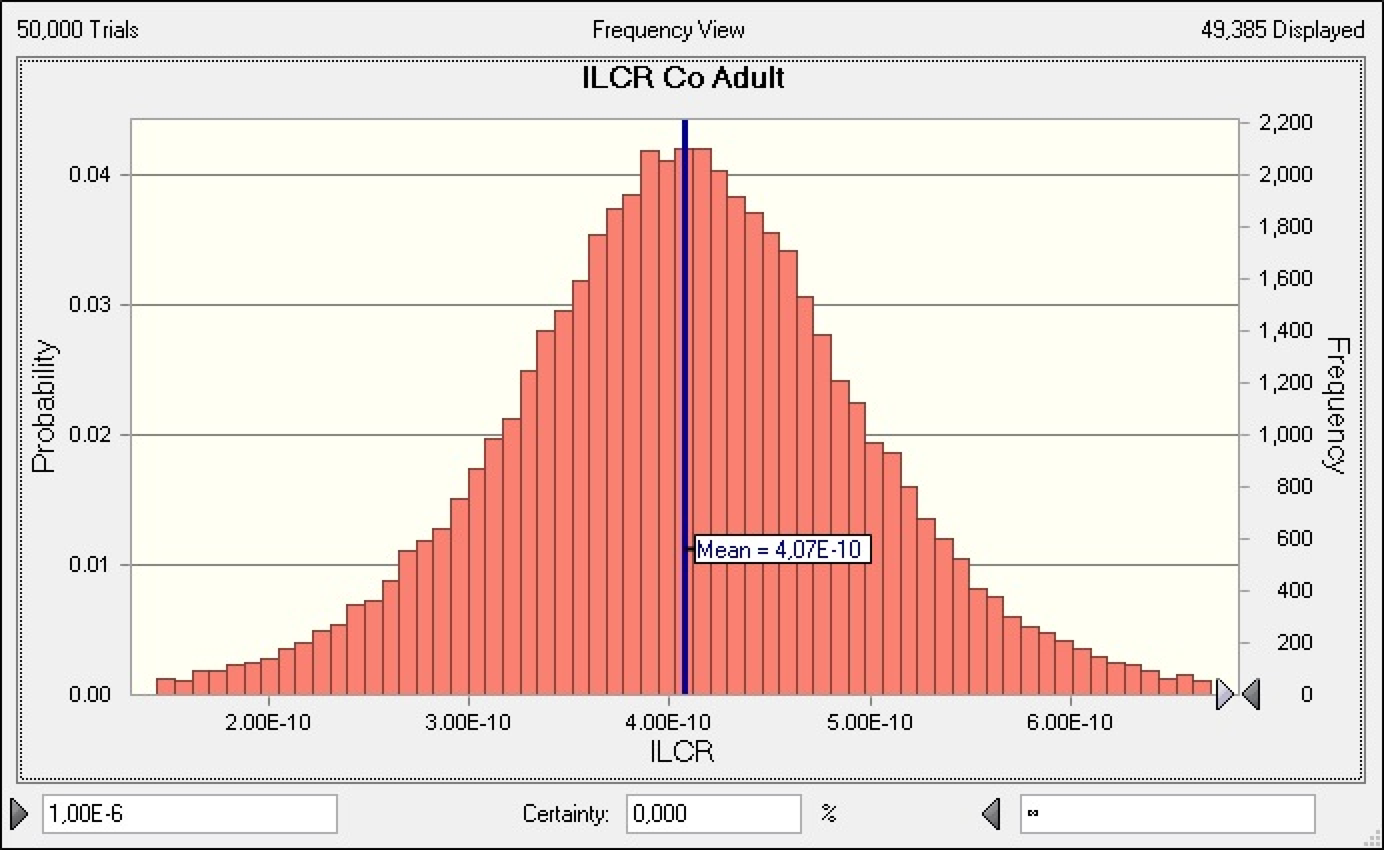

50,000 Trials
Frequency View
49,385 Displayed
ILCR Co Adult
Probability
Frequency
ILCR
Mean = 4,07E-10
0.04
0.03
0.02
0.01
0.00
2,200
2,000
1,800
1,600
1,400
1,200
1,000
800
600
400
200
0
2.00E-10
3.00E-10
4.00E-10
5.00E-10
6.00E-10
1,00E-6
Certainty:
0,000
%
∞

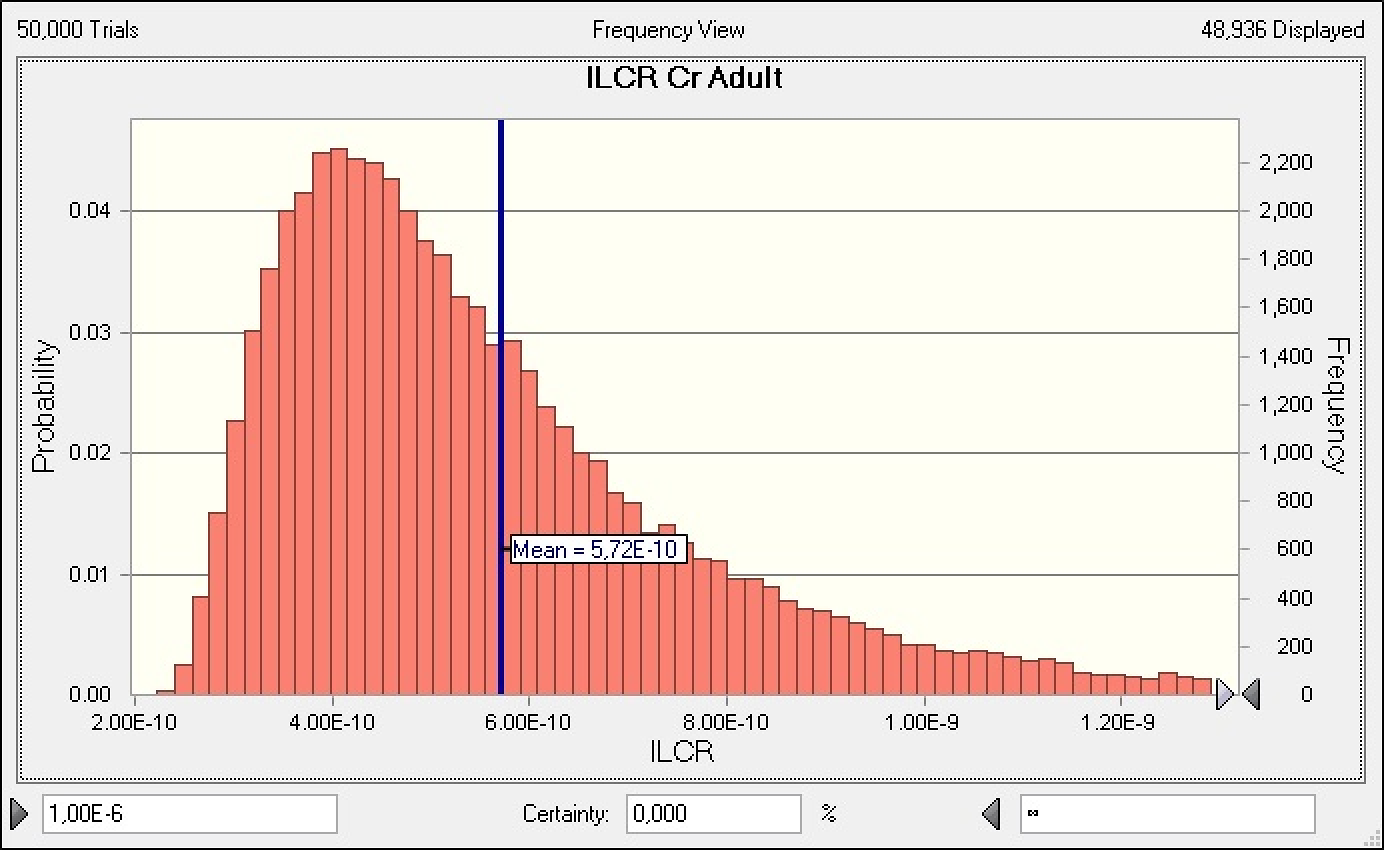

50,000 Trials
Frequency View
48,936 Displayed
ILCR Cr Adult
Probability
Frequency
0.00
0.01
0.02
0.03
0.04
0
200
400
600
800
1,000
1,200
1,400
1,600
1,800
2,000
2,200
Mean = 5,72E-10
2.00E-10
4.00E-10
6.00E-10
8.00E-10
1.00E-9
1.20E-9
ILCR
1,00E-6
Certainty:
0,000
%
∞

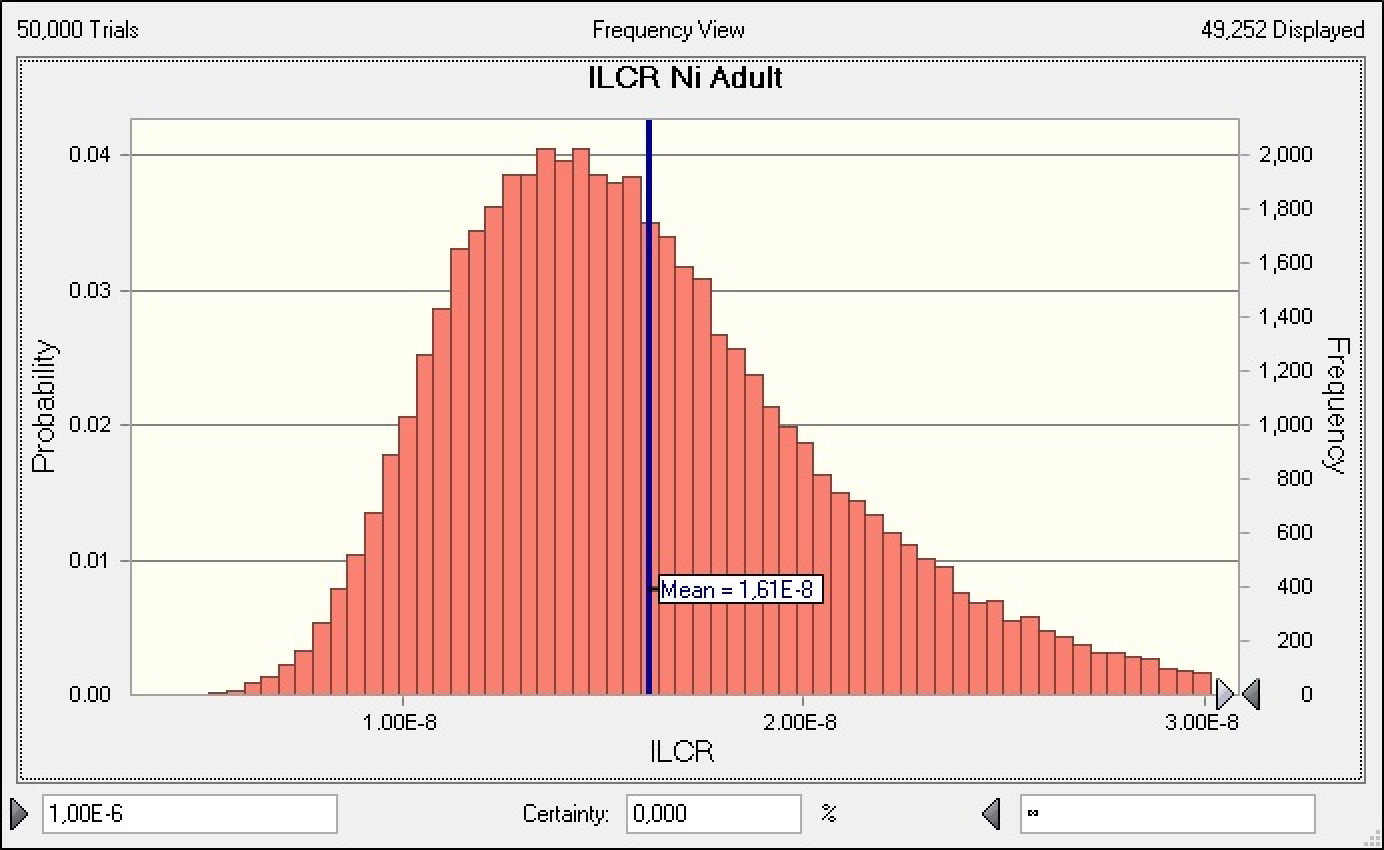
50,000 Trials
Frequency View
49,252 Displayed
ILCR Ni Adult
Probability
Frequency
0.04
0.03
0.02
0.01
0.00
2,000
1,800
1,600
1,400
1,200
1,000
800
600
400
200
0
Mean = 1,61E-8
1.00E-8
2.00E-8
3.00E-8
ILCR
1,00E-6
Certainty:
0,000
%
∞

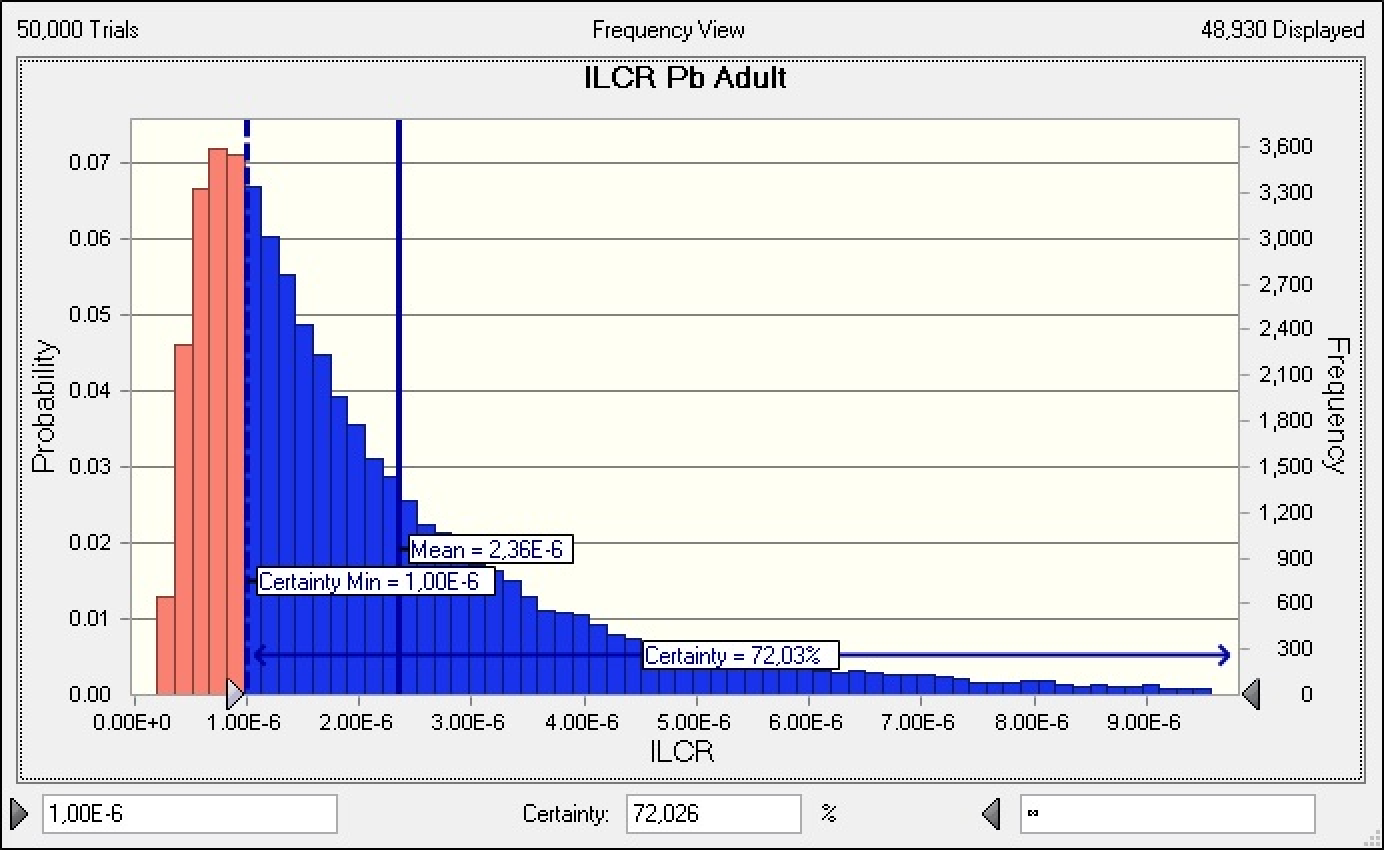

50,000 Trials
Frequency View
48,930 Displayed
ILCR Pb Adult
Probability
Frequency
0.07
0.06
0.05
0.04
0.03
0.02
0.01
0.00
3,600
3,300
3,000
2,700
2,400
2,100
1,800
1,500
1,200
900
600
300
0
Mean = 2,36E-6
Certainty Min = 1,00E-6
Certainty = 72,03%
0.00E+0
1.00E-6
2.00E-6
3.00E-6
4.00E-6
5.00E-6
6.00E-6
7.00E-6
8.00E-6
9.00E-6
ILCR
1,00E-6
Certainty:
72,026
%
∞

ILCRs INGESTION CHILDREN

ILCR ingestion - Child

Element	**As**	**Pb**
Level of certainty	**100.00%**	**18.63%**
Mean	2.58E-05	6.89E-07
Median	2.61E-05	4.65E-07
Min	5.49E-06	6.51E-08
Max	4.27E-05	2.81E-05
St. Dev.	5.12E-06	7.48E-07

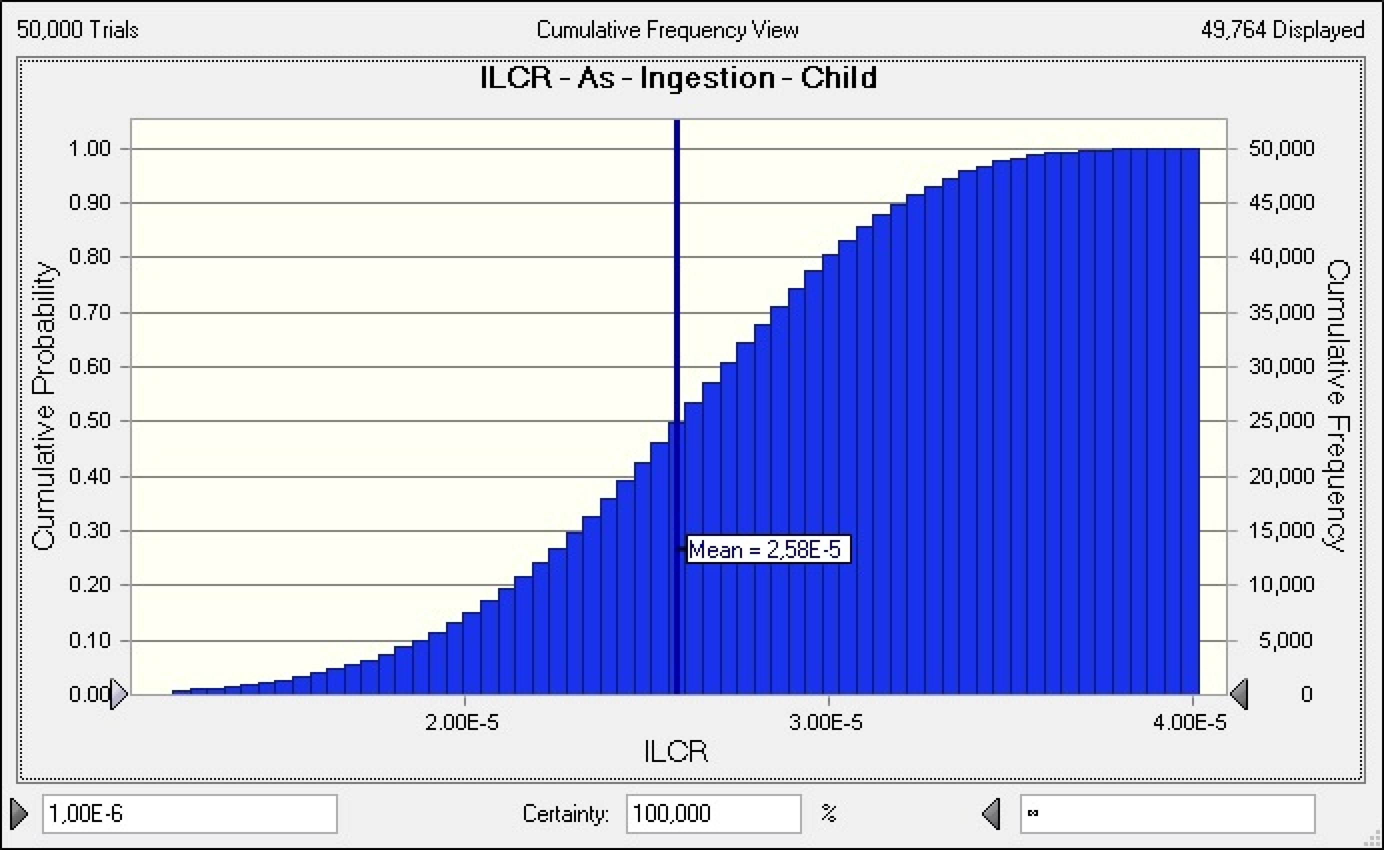

50,000 Trials
Cumulative Frequency View
49,764 Displayed
ILCR - As - Ingestion - Child
Cumulative Probability
1.00
0.90
0.80
0.70
0.60
0.50
0.40
0.30
0.20
0.10
0.00
Cumulative Frequency
50,000
45,000
40,000
35,000
30,000
25,000
20,000
15,000
10,000
5,000
0
Mean = 2,58E-5
2.00E-5
3.00E-5
4.00E-5
ILCR
1,00E-6
Certainty:
100,000
%
∞

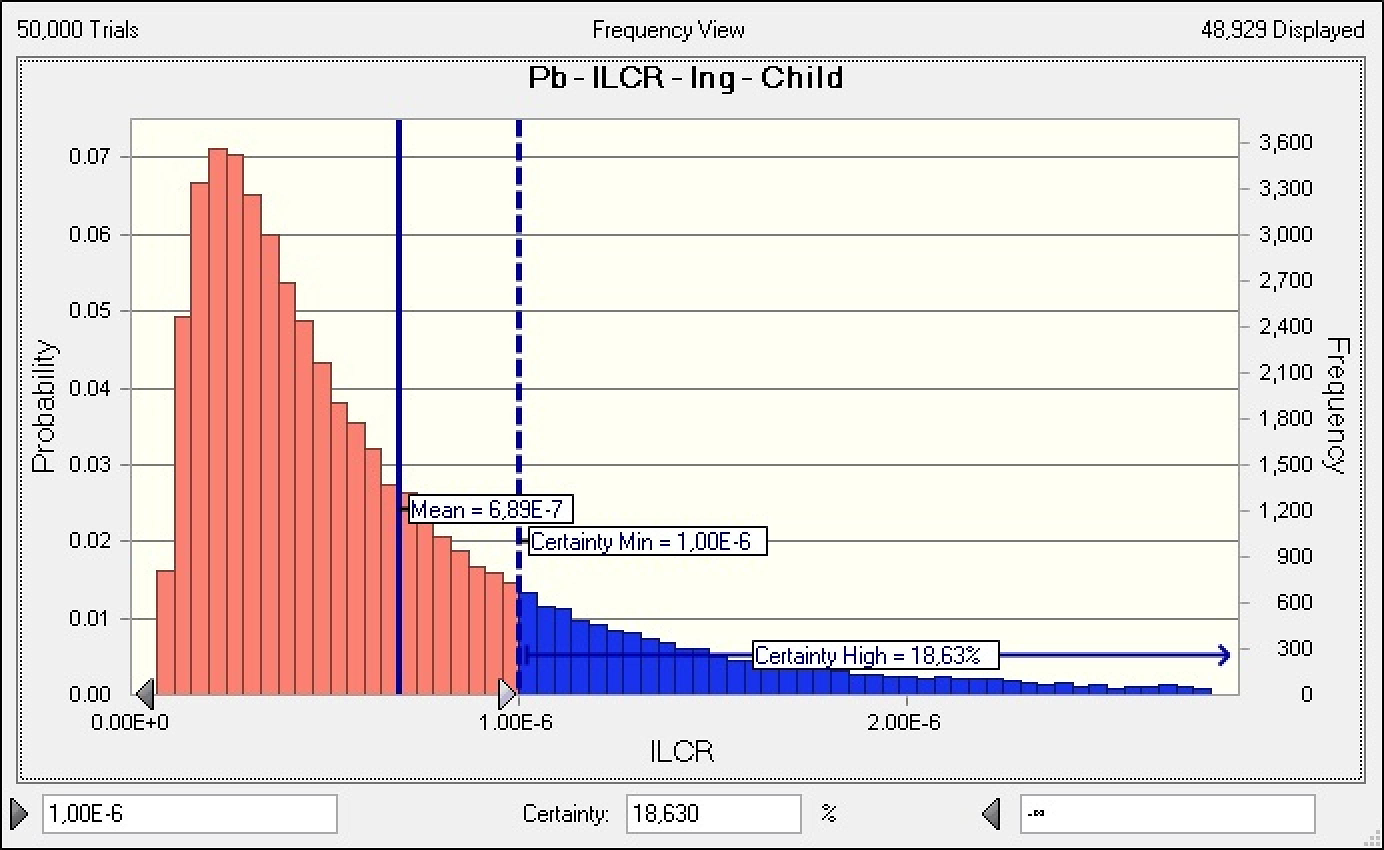

50,000 Trials
Frequency View
48,929 Displayed
Pb - ILCR - Ing - Child
Probability
Frequency
Mean = 6,89E-7
Certainty Min = 1,00E-6
Certainty High = 18,63%
0.07
0.06
0.05
0.04
0.03
0.02
0.01
0.00
3,600
3,300
3,000
2,700
2,400
2,100
1,800
1,500
1,200
900
600
300
0
0.00E+0
1.00E-6
2.00E-6
ILCR
1,00E-6
Certainty:
18,630
%
-∞
